# Graph-Representation of Patient Data: a Systematic Literature Review

**DOI:** 10.1007/s10916-020-1538-4

**Published:** 2020-03-12

**Authors:** Jens Schrodt, Aleksei Dudchenko, Petra Knaup-Gregori, Matthias Ganzinger

**Affiliations:** 1 Institute for Medical Biometry and Informatics, Heidelberg University, Im Neuenheimer Feld 130.3, 69120 Heidelberg, Germany; 2 School of Translational Information Technologies, ITMO University, Kronverksky Pr. 49, 197101 Saint-Petersburg, Russia

**Keywords:** Graph theory, Systematic literature review, Electronic health record, Temporal patient graph

## Abstract

Graph theory is a well-established theory with many methods used in mathematics to study graph structures. In the field of medicine, electronic health records (EHR) are commonly used to store and analyze patient data. Consequently, it seems straight-forward to perform research on modeling EHR data as graphs. This systematic literature review aims to investigate the frontiers of the current research in the field of graphs representing and processing patient data. We want to show, which areas of research in this context need further investigation. The databases MEDLINE, Web of Science, IEEE Xplore and ACM digital library were queried by using the search terms *health record*, *graph* and related terms. Based on the “Preferred Reporting Items for Systematic Reviews and Meta-Analysis” (PRISMA) statement guidelines the articles were screened and evaluated using full-text analysis. Eleven out of 383 articles found in systematic literature review were finally included for analysis in this literature review*.* Most of them use graphs to represent temporal relations, often representing the connection among laboratory data points. Only two papers report that the graph data were further processed by comparing the patient graphs using similarity measurements. Graphs representing individual patients are hardly used in research context, only eleven papers considered such kind of graphs in their investigations. The potential of graph theoretical algorithms, which are already well established, could help increasing this research field, but currently there are too few papers to estimate how this area of research will develop. Altogether, the use of such patient graphs could be a promising technique to develop decision support systems for diagnosis, medication or therapy of patients using similarity measurements or different kinds of analysis.

**Electronic supplementary material:**

The online version of this article (10.1007/s10916-020-1538-4) contains supplementary material, which is available to authorized users.

## Introduction

**Fig. 1:**
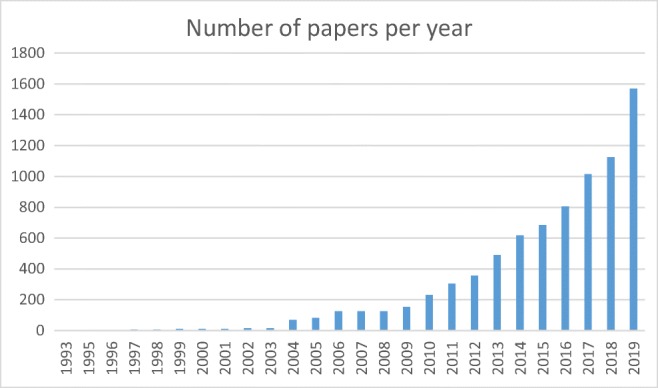
Development of papers per year using the keyword „electronic health record” until 2019

Today, electronic health records (EHR) are the predominant way of documenting health care activities. While the establishment of EHR started decades ago, there are still lively research activities associated with it. Querying PubMed for the term “electronic health record” shows an increasing number of publications year by year until now (cf. Figure 1).

From an information technology perspective, the way how EHR data are persisted has also experienced some development over the years. One of the first approaches that was broadly applied was storing EHR data in relational databases [1]. Today, most hospital information systems are probably based on relational database management software and are accessed with the corresponding Structured Query Language (SQL) [2, 3].

However, the development towards NoSQL database management systems also influenced EHR systems [4, 5]. For instance, document oriented database management systems such as CouchDB [6] or MongoDB [7, 8], no longer require the organization of data in tables. Instead, data are stored as documents written in data formats such as the JavaScript Object Notation (JSON) [9] or Extensible Markup Language (XML) [10]. Another type of NoSQL databases are graph databases [11, 12]. In contrast to document databases, data are stored as properties in structures consisting of nodes and edges. Graph databases have many advantages in comparison to relational databases, for instance, graph databases are much easier to scale, are faster especially at highly connected data, and have a higher level of availability than common relational databases [13].

Graph databases are already in use in various social networks like Facebook and in other Internet companies like Amazon or Google [14]. In social networks, graph databases are very useful because of their property to store the relationships between different members of the social network directly and intuitively (for the user). This direct storage decreases computation time and makes it possible to create queries, which can access these relationships directly. To use these stored relationships of graph databases is also much easier than using the relational data model and its SQL statements for the same purpose. In the relational data model, complex join statements would be necessary to get the same effect and this complicates the creation of queries and increases their computation time compared to graph database queries [14]. And there are also many more fields of applications for graph databases apart from social networks e.g. biomolecular pathways [15], for integration of heterogeneous biological data [16] and for representing disease networks [17].

**Fig. 2:**
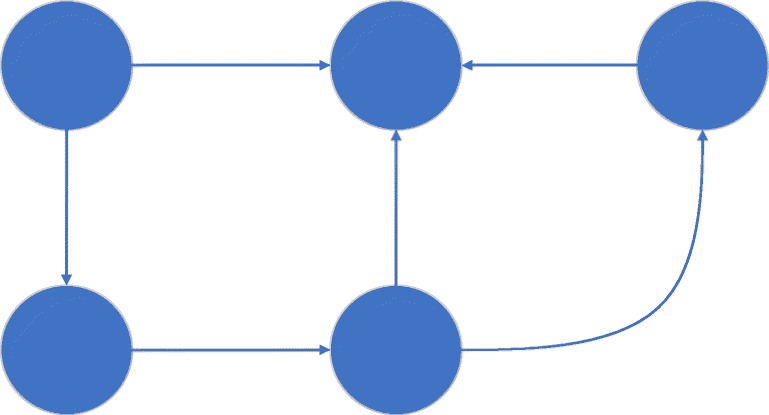
Schematic representation of a directed graph. The dots are called nodes, the connections between the nodes are called edges. The edges are directed, this is shown by the arrow, which points the edge in a direction.

Graphs in context of graph theory are very clearly defined as a set of nodes connected by edges, which represent a relation between the connected nodes [18], this definition is used for the expression *graph* in the following chapters. Figure 2 shows the schematic representation of a graph. The dots are the vertices or nodes, the connections between the nodes represent the edges. Graph theory is a well-established area of mathematics that also covers methods to compare graphs. Apart from graph databases, these methods make the usage of graphs in medical context very interesting, for example to model patient data of EHR systems. With approaches like this, diagnoses, therapies and medications could be suggested on the basis of previous patients and the experiences made at treatment of these patients. Such a system could also be part of a decision support system for physicians in clinical context. For example, graphs are used for spatial description of cerebral anatomy [19] or for clustering of patients and for making a diagnosis [20]. Other approaches are closer related to EHR. Such projects focus for example on visualizing collaborative electronic health record usage with heart failure [11], modeling disease graphs [21] or to predicting knowledge graphs of unknown adverse drug reactions [22].

The aim of this literature review is to investigate the frontiers of the current research in the field of graphs representing and processing patient data. We want to show, which areas of research require further investigation. Before planning research projects in this area we would like to get an overview of already established applications of graphs for individual patients on which, for example, similarity comparisons were performed or the temporal relationships in patient data were used for research projects. To compare patients, it seems to be necessary that patient data are represented by individual graphs or at least sub-graphs. The resulting main questions for the review were:

1. Which kinds of graph-based representations or graph database models of patient data are appropriate established for individual patients?
2. How is the patient data technologically processed after using the graph theoretical framework (e.g. by using graph databases or temporal modeling)?

## Methods

Our systematic literature review is based on the guidelines of the Preferred Reporting Items for Systematic Reviews and Meta-Analyses (PRISMA) approach [23].

### Search strategy

We used the keywords *health records* and *graph* with the synonyms *medical record, patient record* and all plural forms of the keywords for our database search in the databases MEDLINE, Web of Science, IEEE Xplore and ACM digital library. The fields, which were investigated with the search terms, were *title* and *abstract.* The keywords were included in the query syntax of each investigated database. The specific database queries are shown in Suppl. Fig. 1.

### Inclusion criteria

The investigated papers were screened against the following inclusion criteria. The first main criterion was the usage of the term *graph* in the sense of graph theory. This means that the graphs should contain nodes and edges, which is one main definition criterion for graphs of graph theory. Many papers used this word in another context, e.g. as some graphs used the term graph as a synonym for illustration and were therefore excluded. Articles were also excluded if they do not use graphs representing individual patients but for example for a set of patients. Further, only articles written in English or German were included. The database search was done at 20.03.2018 and therefore only articles published and indexed until this date were considered in the review.

### Selection of articles

The articles retrieved from the database queries were screened by four reviewers according to the inclusion criteria based on their title and abstract. If there is no abstract, the full text of the article was used. Initially, all four reviewers tested the inclusion criteria on the same sample of ten articles independently. The results of this test review were discussed afterwards to reach a consensus understanding of the inclusion criteria.

To reduce the workload for the reviewers, the total number of articles was split into two halves, which were assigned to two teams of two reviewers. The members of each team assessed the articles assigned with their partners’ results blinded to ensure that each article received two independent votes. Reviewers marked each article as “included” or “excluded”. For excluded articles, a reason for the exclusion was documented. Articles, which were included by both reviewers, were selected for full-text investigation. Those articles, that were included by one reviewer and excluded by the other reviewer, were assessed by a third reviewer to reach a final decision. The third reviewer decided about inclusion or exclusion of the respective article.

### Data extraction and synthesis

The articles included in the steps before were analyzed in full-text. Some articles still had to be excluded in this phase, because the fulfillment of inclusion criteria, which was recognized in the screening phase, could not be seconded by full-text analysis. To support full-text analysis, the computer-assisted qualitative data analysis software (CAQDAS) MAXQDA was used [24, 25]. In MAXQDA we established a coding system, which was initially created using one article as basis. In a coding system, all central keywords of all investigated and included articles were collected as a hierarchical structure. Each keyword can be assigned to multiple articles and each article can be assigned to multiple keywords. The coding system was iteratively developed by investigating the further articles. Therefore, the papers were loaded in MAXQDA as PDF files to tag the information expressed by the codes in the coding system. Afterwards, cross article occurrences of the different codings were analyzed and main statements were extracted: the kinds of graphs used in the papers, the kinds of data sources, the node and edge contents as well as the processing methods used in the papers.

## Results

### Overall results

By database search, we found 201 hits in MEDLINE, 107 hits in Web of Science, 58 hits in IEEE Xplore and 92 hits in ACM digital library. After eliminating duplicates, the total number of articles was 383. After assessing the inclusion criteria, the reviewers agreed on including 320 of 383 abstracts (84%). For 63 abstracts, they had contradicting opinions, which made the decision of a third reviewer necessary. Finally, 42 abstracts were included by the agreement of the first two reviewers; six further abstracts were added by the third reviewer checking the conflicting articles in abstract (and full-text if necessary). So, in total 48 articles (12.5%) were included, 335 (87.5%) were excluded. The main reasons for exclusion were

- Papers did not use the term graph in a graph theoretical manner
- Papers used graph theory, but the graphs did not represent individual patients (Supplementary Table 3).

After the step of full-text analysis of the 48 articles eleven of these articles finally remained (2.9%) [19, 20, 26–34] for analysis in MAXQDA by using a coding scheme.

### Coding scheme

The coding scheme used in MAXQDA is shown in Suppl. Fig. 3. The main categories after analyzing the eleven remaining articles were *a) data source b) overall purpose / function c) graph properties d) investigated disease e) technical processing of graph.* At first, the methods of representing and saving data in graphs and graph databases were investigated and this investigation can be aligned to the codings *data source* and *graph properties*. Theses codings define how the graphs are generated and what they contain as nodes and edges. The second main question that we investigated was the processing methods of these graphs and their contents. Thus, we investigated the processing methods of these graphs and their contents by using the codings *overall purpose / function* and especially *technical processing of graph.* This second step helps us to understand how currently existing investigations handle the processing of patient graphs and what kinds of goals these investigations would like to reach.

### Graph properties

In Table 1, all different kinds of node contents from the eleven articles included are shown. Six of the papers used laboratory data represented in nodes, five of the papers used medications and diagnosis. Functional nodes were used four times, whereas anatomic nodes and patient problems were used two times each. Procedures, vital signs and patient nodes (a node to identify the patient used in this graph) were used only one time in the papers.

**Table 1:** Overview over all different node contents in the papers. Column 2 shows the number of papers, in which the node content of column 1 was used

Node content	# papers
laboratory data	6
medications	5
diagnoses	5
functional nodes	4
anatomic nodes	2
patient problems	2
procedures	1
vital signs	1
patient nodes	1

In contrast to Table 1, Table 2 shows the content of the edges used in the eleven papers. In two papers, the edges represent causal relations, so the nodes are connected in a causal context. In one paper, the edges represent anatomic-functional relations, whereas in two papers spatial relations were represented by the edges. In detail, the edges show the spatial relations of brain areas. *Taxonomical relations* and *status and date* are two more edge contents used, each in one paper. The edge content most often used by the included articles are *temporal relations*, which were used in six different papers.

**Table 2:** overview over all different edge contents in the papers

Edge content	# papers
Causal relations	**2**
Anatomic-functional relations	**1**
Spatial relations	**2**
Taxonomical relation	**1**
Status and date	**1**
Temporal relations	**6**

### Graph types

Table 3 shows all types of graphs used in the articles to represent electronic health records of a patient. Most of the remaining articles use a representation of electronic health records in a graph representing an individual patient in a temporal manner (temporal event data mining) [20, 27, 28, 30, 32, 33]. In contrast to that, causal networking represents the causal context of patient data and was used in two papers for representing patient data [29, 30]. Heterogeneous data mining was used by one paper and describes the representation of very different kinds of data of the patient in one graph [30] whereas database / data structural approaches were used in two papers. These papers demonstrate possible methods of saving patient data in a graph database or in a graph like structure [26, 34]. Two further papers use the graphs for structural representation of tissue areas in the brain [19, 31].

**Table 3:** Overview over categories of graphs

Graph category	Frequency	Source
Causal networking	2	[29, 30]
Heterogeneous data mining	1	[30]
Database / data structural approach	2	[26, 34]
Structure representation	2	[19, 31]
Temporal event data mining	6	[20, 27, 28, 30, 32, 33]

### Data sources

We also investigated the different data sources for patient data used in the included articles as shown in Table 4. Electronic health records are the biggest part of data sources used in the articles (62.5%). Some articles also use image-based information (12.5%) or data from a healthcare information system (12.5%). One article uses SNOMED CT clinical findings and another one uses a research database (each 6.25%).

**Table 4:** Kinds of data sources used in the included articles

Data sources	Frequency	Percentage	References
Image-based information	2	12,5	[19, 31]
University of Nebraska Medical Center (UNMC) de-identified clinical research database	1	6,25	[26]
SNOMED CT clinical findings	1	6,25	[26]
Electronic Health Record	10	62,5	[20, 26–34]
Healthcare information system	2	12,5	[20, 31]

### Processing of Graphs

Table 5 shows the number of papers, which used the shown kinds of processing methods for patient graphs. Only one paper uses the model for prognosis issues [29]. Five papers investigate the storage of patient graphs in some kind, e.g. in graph databases. In only two papers, the authors are interested in similarity comparisons of the created patient graphs, whereas in nine papers the plain presentation of patient data in a graph plays a central role.

**Table 5:** Overview of all kinds of processing of patient graphs in the included papers

Kind of processing	# papers
Prognostic modelling	1
Storing of graphs	5
Similarity comparison of graphs	2
Presentation of patient data	9

### Goals and Content of the Articles

The research goals described in the different articles differ very much in detail, but an application often mentioned in the articles was personalized medicine, which was named by 6 of the 11 investigated papers. The other goals were quality improvement, information gaining, predictive modeling, disease diagnosis, patient segmentation (each used in 2 papers), population management, data mining, data warehouse and disease pattern (each used in one paper).

To reach these goals the papers follow very different strategies. Atif et al. used image-based information of brains to create a graph-based cerebral description of brain anatomy. This spatial graph is created manually for every patient and afterwards patients could be compared using these graphs [19]. In contrast, Campbell et al. used the SNOMED CT concept model in a graph database architecture because of the ontology character and polyhierarchy of SNOMED CT, which makes it difficult to implement electronic health records in relational databases [26]. The created graphs save SNOMED CT data in a specifically created graph format. Risk prediction is the main goal of Chen et al., so the authors developed a graph-based, semi-supervised learning algorithm to reach this goal [27]. By modelling the clinical evolution of an individual patient with kidney failure Esteban et al. wanted to develop the basis for a future clinical decision support system. This graph-based model contains thousands of events like laboratory results, ordered tests and diagnoses [28] and represents a patient in a graph. Hanzlicek et al. described MUDR EHR, a multimedia distributed health record for decision support. This electronic health record contains multiple medical concepts, which should help describing a patient in a structured way, apart from free text records [34]. Kaur et al. described a model, which combines different data stores of patient data. In this architecture the user creates his request at the interface and the architecture below translates this request into a query to get the data from the most suitable data store for this request [29]. The resulting graph of this paper helps to get the right information from the data stores. Liu et al. used longitudinal patient data to create so called temporal graphs. These graphs were clustered in different phenotypes, so that using these phenotypes helps improving diagnosis performance [20]. The resulting graphs represent a patient and his medical events in temporal context. The focus of Müller et al. was the lack of clinical context in other approaches. The authors solve this problem by creating a graph-grammar approach to design and implement a graph-oriented patient model, which allows the representation of the clinical context [30]. Puentes et al. also used (similar to Atif et al.) graphs to gain information out of image-based brain information to model spatial relationships of brain anatomical singularities of individual patients. This approach is especially used for spatial modelling of cerebral tumors [31]. Zhang et al. [32] created a convolutional neural network on heterogeneous attributes of a patient (e.g. diagnoses, procedures and medications) using a graph, which gains its data from electronic health records [32]. Zhang et al. [33] created a unified graph representation of the electronic health records of an individual patient in a temporal manner. Using this graph, in the second step a modified algorithm was used to create a temporal profile of each patient. This approach was used for risk prediction [33].

## Discussion

### General Findings

Our literature review shows that there are many articles published in context of medical records and graphs, but only a small number of authors investigated graphs in the sense of graph theory or used graphs to represent individual patient data. The initial database query produced a sample size of almost 400 papers. Many of these papers had to be excluded because they used the term graph in a different context than for graph theory. But if the graph mentioned by the paper could be related to graph theory, the second most exclusion criterion often arises: Most of the papers, which use graphs, do not use these graphs to represent data of individual patients. Surprisingly, the exclusion resulted in only eleven papers remaining. From a theoretical point of view, the modeling of patient data as a graph could result in some advantages in analysis of this patient data because of the well-established tools and methods contents of graph theory. The usage of such already established algorithms could facilitate the development of new methods enormously. Research question 1 of this literature review corresponds to possible patient representations in graphs that were used in literature before. Table 1 and Table 2 show the different possibilities of using nodes and edges to represent patient data used in the investigated articles. Especially laboratory data as nodes and temporal relationships between nodes were most commonly used in the investigated papers. The graph type used most often was temporal event data mining, followed by causal networking, structure representation, database structural approaches and heterogeneous data mining. The focus on temporal relationships shows that most data in this field of research was investigated in context of temporal relationships but the low number of papers included in this literature review also shows that there is much more potential for further analysis. With Question 2 we wanted to get an overview of all different processing mechanisms used to investigate the patient graphs. The low number of papers found through the literature review made it very difficult to identify real tendencies, but the main results are shown in Table 5 – only two of the investigated papers do really process the patient data after setting it to a graph. In contrast to that, nine papers use the graphs only to represent the patient data and five papers also use the graphs to store the data in a specific form. This very low number of papers using graphs for representing individual patients and the even lower number of papers processing theses graphs raise different questions for further investigation: Is it reasonable to represent patient data in graphs and process them or is there any reason why this has not been done very often so far? What is the best the way to proof plausibility of such systems?

### Limitations

In this study only the four databases MEDLINE, Web of Science, IEEE Xplore and ACM digital library were used, thus there might be some papers indexed in other databases, which were not found by our review. Apart from the databases, there also might be papers connected to this topic, which would have been captured by using another query. Also publication bias plays a role in literature reviews, especially in this context. The low number of papers using graph theory for representing patient data could also be caused by a high rate of unsuccessful papers in this field of research.

## Conclusion

Our review shows the current state of research in context of patient graphs. The concentration of many of the eleven papers on the recent past might indicate, that this is a rather young research area, which could expand in the next few years, but currently the total number of papers connected to the research field is too low to make a clear statement. Altogether representing a patient in a graph is a very promising technique, which is already used in very different medical areas as shown by the content of the included papers. These different areas (brain tumors, kidney failures, patients in general and so on) show that there is much potential for further studies in this field of research. The possibilities with such systems are very broad and open new opportunities, for example in clinical context. We could imagine a system that helps analyzing patient graphs for finding differential diagnosis, the right medication, or even to get therapy proposals based on experiences made in previous patient cases.

## Electronic supplementary material

ESM 1 (PDF 82 kb)

ESM 2 (PDF 150 kb)

ESM 3 (PDF 89 kb)

ESM 4 (PDF 90.4 kb)

ESM 5 (PDF 1.19 mb)

ESM 6 (PDF 720 kb)

ESM 7 (PDF 1.03 mb)
