# Supplementary material for: Graph-Representation of Patient Data: a Systematic Literature Review
Source: J Med Syst. 2020 Mar 12;44(4):86. doi: 10.1007/s10916-020-1538-4 (PMC7067737; doi:10.1007/s10916-020-1538-4)
Supplement: Supplementary file 1 — (PDF 82 kb) [file 10916_2020_1538_MOESM1_ESM.pdf]

MEDLINE:

((("medical record"[title/abstract]) OR ("patient record"[title/abstract]) OR ("health record"[title/abstract]) OR (health record[MeSH Terms])) AND (graph[title/abstract] OR graphs[title/abstract])) OR ((("medical records"[title/abstract]) OR ("patient records"[title/abstract]) OR ("health records"[title/abstract]) OR (health records[MeSH Terms])) AND (graph[title/abstract] OR graphs[title/abstract]))

Web of Science:

(TS=((("patient record") OR ("health record") OR ("medical record")) AND (graph OR graphs))) OR (TS=((("patient records") OR ("health records") OR ("medical records")) AND (graph OR graphs)))

IEEE Xplore:

((graph OR graphs) AND ("patient record" OR "health record" OR "medical record")) OR ((graph OR graphs) AND ("patient records" OR "health records" OR "medical records"))

ACM digital library:

recordTitle:(+health +record +graph) OR recordAbstract:(+health +record +graph) OR recordTitle:(+medical +record +graph) OR recordAbstract:(+medical +record +graph) OR recordTitle:(+patient +record +graph) OR recordAbstract:(+patient +record +graph) OR recordTitle:(+health +records +graph) OR recordAbstract:(+health +records +graph) OR recordTitle:(+medical +records +graph) OR recordAbstract:(+medical +records +graph) OR recordTitle:(+patient +records +graph) OR recordAbstract:(+patient +records +graph)

Suppl. Fig. 1 queries of database search for the four different databases MEDLINE, Web of Science, IEEE Xplore, ACM digital library.

Title: Graph-Representation of Patient Data
A Systematic Literature Review

Journal: Journal of Medical Systems

Authors:

Jens Schrodt, jens.schrodt@med.uni-heidelberg.de Universitätsklinikum Heidelberg Institut für Medizinische Biometrie und Informatik, Im Neuenheimer Feld 130.3, 69120 Heidelberg, Germany, orcid: 0000-0002-9768-4781

Aleksei Dudchenko, Aleksei.Dudchenko@med.uni-heidelberg.de Universitätsklinikum Heidelberg Institut für Medizinische Biometrie und Informatik, Im Neuenheimer Feld 130.3, 69120 Heidelberg, Germany

Petra Knaup, petra.knaup@med.uni-heidelberg.de Universitätsklinikum Heidelberg Institut für Medizinische Biometrie und Informatik, Im Neuenheimer Feld 130.3, 69120 Heidelberg, Germany

Matthias Ganzinger, matthias.ganzinger@med.uni-heidelberg.de Universitätsklinikum Heidelberg Institut für Medizinische Biometrie und Informatik, Im Neuenheimer Feld 130.3, 69120 Heidelberg, Germany
