# Supplementary material for: Graph-Representation of Patient Data: a Systematic Literature Review
Source: J Med Syst. 2020 Mar 12;44(4):86. doi: 10.1007/s10916-020-1538-4 (PMC7067737; doi:10.1007/s10916-020-1538-4)
Supplement: Supplementary file 3 — (PDF 89 kb) [file 10916_2020_1538_MOESM3_ESM.pdf]

Title: Graph-Representation of Patient Data
A Systematic Literature Review

Journal: Journal of Medical Systems

Authors:

Jens Schrodt, jens.schrodt@med.uni-heidelberg.de Universitätsklinikum Heidelberg Institut für Medizinische Biometrie und Informatik, Im Neuenheimer Feld 130.3, 69120 Heidelberg, Germany, orcid: 0000-0002-9768-4781

Aleksei Dudchenko, Aleksei.Dudchenko@med.uni-heidelberg.de Universitätsklinikum Heidelberg Institut für Medizinische Biometrie und Informatik, Im Neuenheimer Feld 130.3, 69120 Heidelberg, Germany

Petra Knaup, petra.knaup@med.uni-heidelberg.de Universitätsklinikum Heidelberg Institut für Medizinische Biometrie und Informatik, Im Neuenheimer Feld 130.3, 69120 Heidelberg, Germany

Matthias Ganzinger, matthias.ganzinger@med.uni-heidelberg.de Universitätsklinikum Heidelberg Institut für Medizinische Biometrie und Informatik, Im Neuenheimer Feld 130.3, 69120 Heidelberg, Germany

Suppl. Fig. 3: coding system created in MAXQDA

- Codesystem [211]
    - categorization [0]
    - heterogeneous data mining [1]
    - causal networking [2]
    - database/data structure approach, SNOMED [1]
    - structure representation [2]
    - temporal event data mining [6]
- data source [0]
    - free-text records [1]
    - paper health records [1]
    - image-based information [2]
    - UNMC de-identified clinical research database [1]
    - SNOMED CT clinical findings [2]
    - Electronic Health Record (EHR) [11]
    - healthcare information system [2]
- overall purpose / function [0]
    - drug investigation [1]
    - information gaining [3]
    - quality improvement [2]
    - population management [1]
    - data mining [1]
    - data warehouse [1]
    - predictive modeling [2]
    - disease diagnosis [2]
    - disease pattern [1]
    - patient segmentation [2]
    - personalized medicine [6]
- graph [1]
    - patient graph [4]
    - node content [0]
        - chemical structure of drug [1]
        - words [1]
        - observation [1]

        - emotion [1]
        - procedures [2]
        - functional vertices [4]
        - matter [1]
        - anatomic parts [3]
        - patient [2]
        - diagnosis [7]
        - Electronic Health Records [1]
        - clinical notes [1]
        - problems [2]
        - medications [5]
        - vital signs [1]
        - laboratory data [7]
    - edge content [0]
        - causal relation [2]
        - properties [1]
        - word link [1]
        - patient drug prior associations [1]
        - drug similarity [1]
        - patient similarity [1]
        - number of combinations between nodes [0]
        - Anatomo-Functional relations [1]
        - matter relation [1]
        - spatial relations [2]
        - taxonomical relation [1]
        - status and date [1]
        - temporal relationships [6]
    - graph properties [0]
        - undirected [0]
        - directed [8]
        - unweighted graph [0]
        - weighted graph [5]
            - average duration [2]
        - content of subgraphs [0]
            - detected phenotypes [1]
    - supervision of information [0]
        - supervised information [2]
        - semi-supervised information [2]
        - unsupervised information [1]
- investigated diseases [0]
    - (childhood) leukemia [1]
    - coronary heart diseases (CHD) [1]
    - COPD [1]
    - hypercholesterolemia [1]
    - diabetes [1]
    - kardiovascular disease [1]
    - kidney failure [1]
    - geriatric diseases [1]
    - brain tumors [1]
    - pleural effusion [1]
    - Congestive Heart Failure (CHF) [2]
    - CHF with COPD precondition [2]
    - pneumonia due to an influenza [1]
    - parainfluenza virus [1]
    - breast cancer [2]
- investigated diseases ICD [0]
    - C71.* | D33.* | D43.* brain tumors [1]
    - C50.* breast cancer [2]

- C95.* (childhood) leukemia [1]
- I25.* coronary heart diseases (CHD) [1]
- I25.* | I51.9 kardiovascular disease [1]
- I50.9 Congestive Heart Failure (CHF) [2]
- I50.9 | J44.* CHF with COPD precondition [2]
- J44.* COPD [1]
- J90.* pleural effusion [1]
- J10.0 pneumonia due to an influenza [1]
- J12.2 parainfluenza virus [1]
- E78.0 hypercholesterolemia [1]
- E11.* diabetes [1]
- N19.* kidney failure [1]
- geriatric diseases [1]

investigated diseases zusammengefasst [0]

- cancer [4]
- heart disease [6]
- lung disease [6]
- E78.0 hypercholesterolemia [1]
- E11.* diabetes [1]
- N19.* kidney failure [1]
- geriatric diseases [1]

processing of graph (technical) [0]

- predictive modeling [1]
- kind of storage [0]
  - GraphML [2]
  - graph database [1]
    - MongoDB [1]
    - neo4j [2]
  - SNOMED [2]
  - RDF [1]
- conversion [0]
  - SNOMED to neo4j [1]
- clustering [2]
- similarity comparison of graphs [3]

Sets [0]
