# Supplementary material for: Graph-Representation of Patient Data: a Systematic Literature Review
Source: J Med Syst. 2020 Mar 12;44(4):86. doi: 10.1007/s10916-020-1538-4 (PMC7067737; doi:10.1007/s10916-020-1538-4)
Supplement: Supplementary file 4 — (PDF 90.4 kb) [file 10916_2020_1538_MOESM4_ESM.pdf]

Suppl. Table 1 chapters of ICD-10 code scheme and the number of diseases investigated in the chapters by the investigated articles.

Chapter	Blocks	Title	number
I	A00–B99	Certain infectious and parasitic diseases	
II	C00–D48	Neoplasms	4
III	D50–D89	Diseases of the blood and blood-forming organs and certain disorders involving the immune mechanism	
IV	E00–E90	Endocrine, nutritional and metabolic diseases	2
V	F00–F99	Mental and behavioural disorders	
VI	G00–G99	Diseases of the nervous system	
VII	H00–H59	Diseases of the eye and adnexa	
VIII	H60–H95	Diseases of the ear and mastoid process	
IX	I00–I99	Diseases of the circulatory system	7
X	J00–J99	Diseases of the respiratory system	6
XI	K00–K93	Diseases of the digestive system	
XII	L00–L99	Diseases of the skin and subcutaneous tissue	

XIII	M00–M99	Diseases of the musculoskeletal system and connective tissue	
XIV	N00–N99	Diseases of the genitourinary system	1
XV	O00–O99	Pregnancy, childbirth and the puerperium	
XVI	P00–P96	Certain conditions originating in the perinatal period	
XVII	Q00–Q99	Congenital malformations, deformations and chromosomal abnormalities	
XVIII	R00–R99	Symptoms, signs and abnormal clinical and laboratory findings, not elsewhere classified	
XIX	S00–T98	Injury, poisoning and certain other consequences of external causes	
XX	V01–Y98	External causes of morbidity and mortality	
XXI	Z00–Z99	Factors influencing health status and contact with health services	
XXII	U00–U99	Codes for special purposes	

Suppl. Table 2 Agreement of reviewers in the first step

1st reviewer / 2nd reviewer	Include	Exclude	Sum
**Include**	43	32	**75**
**Exclude**	31	277	**308**
**Sum**	**74**	**309**	**383**
