# Supplementary material for: Graph-Representation of Patient Data: a Systematic Literature Review
Source: J Med Syst. 2020 Mar 12;44(4):86. doi: 10.1007/s10916-020-1538-4 (PMC7067737; doi:10.1007/s10916-020-1538-4)
Supplement: Supplementary file 5 — (PDF 1.19 mb) [file 10916_2020_1538_MOESM5_ESM.pdf]

Title: Graph-Representation of Patient Data
A Systematic Literature Review

Journal: Journal of Medical Systems

Authors:

Jens Schrodt, jens.schrodt@med.uni-heidelberg.de Universitätsklinikum Heidelberg Institut für Medizinische Biometrie und Informatik, Im Neuenheimer Feld 130.3, 69120 Heidelberg, Germany, orcid: 0000-0002-9768-4781

Aleksei Dudchenko, Aleksei.Dudchenko@med.uni-heidelberg.de Universitätsklinikum Heidelberg Institut für Medizinische Biometrie und Informatik, Im Neuenheimer Feld 130.3, 69120 Heidelberg, Germany

Petra Knaup, petra.knaup@med.uni-heidelberg.de Universitätsklinikum Heidelberg Institut für Medizinische Biometrie und Informatik, Im Neuenheimer Feld 130.3, 69120 Heidelberg, Germany

Matthias Ganzinger, matthias.ganzinger@med.uni-heidelberg.de Universitätsklinikum Heidelberg Institut für Medizinische Biometrie und Informatik, Im Neuenheimer Feld 130.3, 69120 Heidelberg, Germany

Suppl. Table 3:

Author, editor or organization	Title	Year	Ref.	Reviewer 1 reason	Reviewer 2 reason	Reviewer 3 reason
	Abbs v. Sullivan	1992	[1]	graph is not associated to an individual patient	term “graph” is used for something different than for patient graphs	
**Adin, C. A.; Gregory, C. R.; Kyles, A. E.; Cowgill, L.**	Diagnostic predictors of complications and survival after renal transplantation in cats	2001	[2]	term “graph” is used for something different than for patient graphs	No graph mentioned	
**Agogo, George O.; van der Voet, Hilko; van't Veer, Pieter; Ferrari, Pietro; Leenders, Max; Muller, David C.; Sanchez-Cantalejo, Emilio; Bamia, Christina; Braaten, Tonje; Knuppel, Sven; Johansson, Ingegerd; van Eeuwijk, Fred A.; Boshuizen, Hendriek**	Use of two-part regression calibration model to correct for measurement error in episodically consumed foods in a single-replicate study design: EPIC case study	2014	[3]	term “graph” is used for something different than for patient graphs	No graph mentioned	
**Amit, Moran; Yen, Tzu Chen; Liao, Chun Ta; Chaturvedi, Pankaj; Agarwal, Jai Prakash; Kowalski, Luiz Paulo; Kohler, Hugo F.; Ebrahimi, Ardalan; Clark, Jonathan R.; Cernea, Claudio Roberto;**	The origin of regional failure in oral cavity squamous cell carcinoma with pathologically negative neck metastases	2014	[4]	term “graph” is used for something different than for patient graphs	No graph mentioned	

**Brandao, Jose S.; Kreppel, Matthias; Zoller, Joachim E.; Leider-Trejo, Leonor; Bachar, Gideon; Shpitzer, Thomas; Bolzoni, Andrea Villaret; Patel, Raj P.; Jonnalagadda, Sashikanth; Robbins, Thomas Kevin; Shah, Jatin P.; Patel, Snehal G.; Gil, Ziv**						
**Andreano, Anita; Rebora, Paola; Valsecchi, Maria Grazia; Russo, Antonio Giampiero**	Adherence to guidelines and breast cancer patients survival: a population-based cohort study analyzed with a causal inference approach	2017	[5]	term “graph” is used for something different than for patient graphs	No graph theory	
**Anindya, Imrul Chowdhury; Roy, Harichandan; Kantarcioglu, Murat; Malin, Bradley**	Building a Dossier on the Cheap: Integrating Distributed Personal Data Resources Under Cost Constraints	2017	[6]	term “graph” is used for something different than for patient graphs	No EHR data	
**Antani, S.**	Integrating image and text information for biomedical information retrieval	2010	[7]	term “graph” is used for something different than for patient graphs	Graph as image	
**Anwar, Muhammad Naveed; Oakes, Michael Philip**	Data Mining of Audiology Patient Records: Factors Influencing the Choice of Hearing Aid Type	2011	[8]	term “graph” is used for something different than for patient graphs	Graph used for audiogram	
**Anwar, Muhammad N.; Oakes, Michael P.**	Data mining of audiology patient records: factors influencing the choice of hearing aid type	2012	[9]	term “graph” is used for something different than for patient graphs	Graph used for audiogram	
**Arcilla, Mary Jane B.; Ang, Viana Celina T.; Ochoa, Mikaela Nazarene; Padua, Christian Paulo L.; Payawal, Riezl E.**	Kidnew: The Kidney Transplant Patient Personal Health Buddy	2015	[10]	term “graph” is used for something different than for patient graphs	Graph as image	
**Atreja, Ashish; Khan, Sameer; Rogers, Jason D.; Otobo, Emamuzo; Patel, Nishant P.; Ullman, Thomas; Colombel, Jean Fred; Moore, Shirley; Sands, Bruce E.**	Impact of the Mobile HealthPROMISE Platform on the Quality of Care and Quality of Life in Patients With Inflammatory Bowel Disease: Study Protocol of a Pragmatic Randomized Controlled Trial	2015	[11]	term “graph” is used for something different than for patient graphs	Graph as image	
**Bakel, Leigh Anne; Wilson, Karen; Tyler, Amy; Tham, Eric; Reese, Jennifer; Bothner, Joan; Kaplan, David W.**	A quality improvement study to improve inpatient problem list use	2014	[12]	term “graph” is used for something different than for patient graphs	Graph as image	
**Baptista Macaroff, W. M.; Castroman Espasandin, P.**	Analgesic quality in a postoperative pain service: continuous assessment with the cumulative sum (cusum) method	2007	[13]	term “graph” is used for something different than for patient graphs	Hospital performance modelled	
**Barsky, Marina; Stege, Ulrike; Thomo, Alex; Upton, Chris**	A Graph Approach to the Threshold All-against-all Substring Matching Problem	2008	[14]	term “graph” is used for something different than for patient graphs	no patient data	
**Bassily, Mena N.; Wilson, Richard; Pompei, Francesco; Burmistrov, Dimitriy**	Cancer survival as a function of age at diagnosis: a study of the Surveillance, Epidemiology and End Results database	2010	[15]	term “graph” is used for something different than for patient graphs	Graph as image	

**Baud, R. H.; Am RASSINOUX; SCHERRER, [JR]**	NATURAL-LANGUAGE PROCESSING AND SEMANTICAL REPRESENTATION OF MEDICAL TEXTS	1992	[16]	graph is not associated to an individual patient	no patient data	
**Baud, R. H.; Am RASSINOUX; WAGNER, J. C.; Lovis, C.; JUGE, C.; ALPAY, L. L.; Michel, P. A.; Degoulet, P.; SCHERRER, [JR]**	REPRESENTING CLINICAL NARRATIVES USING CONCEPTUAL GRAPHS	1995	[17]	graph is not associated to an individual patient	focus on laguage processing	
**Baud, R. H.; Rassinoux, A. M.; Scherrer, J. R.**	Natural language processing and semantical representation of medical texts	1992	[18]	Dublette	focus on laguage processing	
**Bean, Daniel M.; Wu, Honghan; Iqbal, Ehtesham; Dzahini, Olubanke; Ibrahim, Zina M.; Broadbent, Matthew; Stewart, Robert; Dobson, Richard J. B.**	Knowledge graph prediction of unknown adverse drug reactions and validation in electronic health records	2017	[19]	graph is not associated to an individual patient	no patient data modelled	
**Bean, Daniel M.; Wu, Honghan; Iqbal, Ehtesham; Dzahini, Olubanke; Ibrahim, Zina M.; Broadbent, Matthew; Stewart, Robert; Dobson, Richard J. B.**	Author Correction: Knowledge graph prediction of unknown adverse drug reactions and validation in electronic health records	2018	[20]	Dublette	no patient data modelled	
**Beilin, L. J.; Bulpitt, C. J.; Coles, E. C.; Dollery, C. T.; Johnson, B. F.; Mearns, C.; Munro-Faure, A. D.; Turner, S. C.**	Computer-based hypertension clinic records: a co-operative study	1974	[21]	term “graph” is used for something different than for patient graphs	Graph as image	
**Bellin, Eran; Kalkut, Gary**	Is time-slice analysis superior to total hospital length of stay in demonstrating the effectiveness of a month-long intensive effort on a medicine service?	2004	[22]	term “graph” is used for something different than for patient graphs	graph as visualization	
**Benedik, Peter; Rajkovic, Uros; Sustersic, Olga**	Toward the design of a nursing ontology system	2014	[23]	graph is not associated to an individual patient	no patient data modelled	
**Bereg, Sergey; Zhang, Yuanyi**	Phylogenetic Networks Based on the Molecular Clock Hypothesis	2007	[24]	term “graph” is used for something different than for patient graphs	No graph theory mentioned	
**Bernauer, J.; Franz, M.; Schoop, D.; Schoop, M.; Pretschner, D. P.**	The compositional approach for representing medical concept systems	1995	[25]	graph is not associated to an individual patient	focus on laguage processing	
**Bertrand, Denis; Gascuel, Olivier**	Topological Rearrangements and Local Search Method for Tandem Duplication Trees	2005	[26]	term “graph” is used for something different than for patient graphs	no patient data modelled	
**Bingham, A.**	Computerized patient records benefit physician offices	1997	[27]	term “graph” is used for something different than for patient graphs	Graph as image	
**Bono, B. de; Helvensteijn, M.; Kokash, N.; Martorelli, I.; Sarwar, D.; Islam, S.; Grenon, P.; Hunter, P.**	Requirements for the formal representation of pathophysiology mechanisms by clinicians	2016	[28]	graph is not associated to an individual patient	No graph mentioned	
**Bordewich, Magnus; Gascuel, Olivier; Huber, Katharina T.; Moulton, Vincent**	Consistency of Topological Moves Based on the Balanced Minimum Evolution Principle of Phylogenetic Inference	2009	[29]	term “graph” is used for something different than for patient graphs	no patient data modelled	

**Brewer, Noel T.; Gilkey, Melissa B.; Lillie, Sarah E.; Hesse, Bradford W.; Sheridan, Stacey L.**	Tables or bar graphs? Presenting test results in electronic medical records	2012	[30]	term “graph” is used for something different than for patient graphs	graph as visualization	
**Britto, Maria T.; Jimison, Holly B.; Munafo, Jennifer Knopf; Wissman, Jennifer; Rogers, Michelle L.; Hersh, William**	Usability testing finds problems for novice users of pediatric portals	2009	[31]	term “graph” is used for something different than for patient graphs	graph as visualization	
**Brown, A. L.**	Constructing Chromosome Scale Suffix Trees	2004	[32]	term “graph” is used for something different than for patient graphs	No graph theory	
**Buchtela, David; Anger, Zdenek; Peleska, Jan; Vesely, Arnost; Zvarova, Jana**	Presentation of medical guidelines on a computer	2004	[33]	term “graph” is used for something different than for patient graphs	no patient data but medcial guidelines modelled	
**Bui, Aleex A. T.; Taira, Ricky K.; Churchill, Bernard; Kangarloo, Hooshang**	Integrated visualization of problemcentric urologic patient records	2002	[34]	term “graph” is used for something different than for patient graphs	graph as visualization	
**Burgess, H. A.**	Use of the labor graph in Malawi	1986	[35]	term “graph” is used for something different than for patient graphs	graph as visualization	
**Butch, S. H.**	Practical use of computerized hospital information systems to improve blood transfusion	1997	[36]	term “graph” is used for something different than for patient graphs	graph as visualization	
**Cates, W. [JR]**	Legal abortion: the public health record	1982	[37]	term “graph” is used for something different than for patient graphs	No graph theory	
**Cen, Songxiang; Han, Li; Ma, Jian**	Ranking Weblogs by Analyzing Reading and Commenting Activities	2009	[38]	graph is not associated to an individual patient	no patient data modelled	
**Chazelle, Bernard**	Technical Perspective: Finding a Good Neighbor, Near and Fast	2008	[39]	term “graph” is used for something different than for patient graphs	no patient data	
**Chen, Peng-Lai; Zhao, Ting; Feng, Rui; Chai, Jing; Tong, Gui-Xian; Wang, De-Bin**	Patterns and trends with cancer incidence and mortality rates reported by the China National Cancer Registry	2014	[40]	term “graph” is used for something different than for patient graphs	graph as visualization	
**Chennamsetty, H.; Chalasani, S.; Riley, D.**	Predictive analytics on Electronic Health Records (EHRs) using Hadoop and Hive	2015	[41]	term “graph” is used for something different than for patient graphs	Graph as image	
**Christinat, Yann; Moret, Bernard M. E.**	A Transcript Perspective on Evolution	2013	[42]	term “graph” is used for something different than for patient graphs	no patient data	
**Christofidis, Melany J.; Hill, Andrew; Horswill, Mark S.; Watson, Marcus O.**	Observation charts with overlapping blood pressure and heart rate graphs do not yield the performance advantage that health professionals assume: an experimental study	2014	[43]	term “graph” is used for something different than for patient graphs	graph as visualization	

**Chung, Hyun Hoon; Kim, Jae Weon; Kang, Keon Wook; Park, Noh-Hyun; Song, Yong-Sang; Chung, June-Key; Kang, Soon-Beom**	Post-treatment (1)(8)FFDG maximum standardized uptake value as a prognostic marker of recurrence in endometrial carcinoma	2011	[44]	term “graph” is used for something different than for patient graphs	No graph theory	
**Cimino, J. J.; Clayton, P. D.; Hripcsak, G.; Johnson, S. B.**	Knowledge-based approaches to the maintenance of a large controlled medical terminology	1994	[45]	graph is not associated to an individual patient	no patient data but medical terminology modelled	
**Collier, Timothy; Steenkamp, Retha; Tomson, Charlie; Caskey, Fergus; Ansell, David; Roderick, Paul; Nitsch, Dorothea**	Patterns and effects of missing comorbidity data for patients starting renal replacement therapy in England, Wales and Northern Ireland	2011	[46]	term “graph” is used for something different than for patient graphs	graph as visualization	
**Cota, Erika; Ribeiro, Leila; Bezerra, Jonas Santos; Costa, Andrei; da Silva, Rosiana Estefane; Cota, Glaucia**	Using formal methods for content validation of medical procedure documents	2017	[47]	graph is not associated to an individual patient	no patient data	
**Cruz, J. P.; Libatique, N. J.; Tangonan, G.**	Steganography and data hiding in flash video (FLV)	2012	[48]	term “graph” is used for something different than for patient graphs	no patient data	
**Cypko, M. A.; Wojdziak, J.; Stoehr, M.; Kirchner, B.; Preim, B.; Dietz, A.; Lemke, H. U.; Oeltze-Jafra, S.**	Visual Verification of Cancer Staging for Therapy Decision Support	2017	[49]	graph is not associated to an individual patient	graph as visualization	
**Dabek, F.; Caban, J. J.**	A grammar-based approach to model the patient’s clinical trajectory after a mild traumatic brain injury	2015	[50]	graph is not associated to an individual patient	no data of individual patients	
**Dabek, Filip; Chen, Jian; Garbarino, Alexander; Caban, Jesus J.**	Visualization of Longitudinal Clinical Trajectories Using a Graph-based Approach	2015	[51]	graph is not associated to an individual patient	no data of individual patients	
**Dahl, Mads Ronald; Vedsted, Peter**	Personal data and confidentiality on the Internet	2008	[52]	term “graph” is used for something different than for patient graphs	no data of individual patients	
**Danaei, Goodarz; Garcia Rodriguez, Luis A.; Fernandez Cantero, Oscar; Logan, Roger; Hernan, Miguel A.**	Observational data for comparative effectiveness research: An emulation of randomised trials of statins and primary prevention of coronary heart disease	2013	[53]	term “graph” is used for something different than for patient graphs	No graph theory mentioned	
**Davidson, Shmuel; Natan, Dafna; Novikov, Ilya; Sokolover, Nir; Erlich, Avi; Shamir, Raanan**	Body mass index and weight-for-length ratio references for infants born at 33-42 weeks gestation: a new tool for anthropometric assessment	2011	[54]	term “graph” is used for something different than for patient graphs	Graph as image	
**Degnan, James H.; Rosenberg, Noah A.; Stadler, Tanja**	A Characterization of the Set of Species Trees That Produce Anomalous Ranked Gene Trees	2012	[55]	term “graph” is used for something different than for patient graphs	graph as visualization	
**Delman, B. S.**	A problem-oriented approach to journal selection for hospital libraries	1982	[56]	term “graph” is used for something different than for patient graphs	no patient data	

**Dent, R. M.; Penwarden, R. M.; Harris, N.; Hotz, S. B.**	Development and evaluation of patient-centered software for a weight-management clinic	2002	[57]	term “graph” is used for something different than for patient graphs	graph as visualization	
**Dietz, Kelly R.; Zhang, Lei; Seidel, Frank G.**	The transverse diameter of the chest on routine radiographs reliably estimates gestational age and weight in premature infants	2015	[58]	term “graph” is used for something different than for patient graphs	Graph as image	
**Diprose, G. K.; Evans, D. H.; Levene, M. I.**	A microcomputer monitoring and data-acquisition system for intensive care units	1985	[59]	term “graph” is used for something different than for patient graphs	graph as visualization	
**Elliott, Doug; Allen, Emily; McKinley, Sharon; Perry, Lin; Duffield, Christine; Fry, Margaret; Gallagher, Robyn; Iedema, Rick; Roche, Michael**	User acceptance of observation and response charts with a track and trigger system: a multisite staff survey	2016	[60]	term “graph” is used for something different than for patient graphs	graph as visualization	
**Engholm, Gerda; Ferlay, Jacques; Christensen, Niels; Bray, Freddie; Gjerstorff, Marianne L.; Klint, Asa; Kotlum, Joanis E.; Olafsdottir, Elinborg; Pukkala, Eero; Storm, Hans H.**	NORDCAN--a Nordic tool for cancer information, planning, quality control and research	2010	[61]	term “graph” is used for something different than for patient graphs	graph as visualization	
**Erkal, Sibel; Gerberich, Susan Goodwin; Ryan, Andrew D.; Renier, Colleen M.; Alexander, Bruce H.**	Animal-related injuries: a population-based study of a five-state region in the upper Midwest: Regional Rural Injury Study II	2008	[62]	graph is not associated to an individual patient	no data of individual patients	
**Evandt, Jorunn; Oftedal, Bente; Krog, Norun Hjertager; Skurtveit, Svetlana; Nafstad, Per; Schwarze, Per E.; Skovlund, Eva; Houthuijs, Danny; Aasvang, Gunn Marit**	Road traffic noise and registry based use of sleep medication	2017	[63]	term “graph” is used for something different than for patient graphs	No graph theory	
**Evans, J. M.; MacDonald, T. M.**	Misclassification and selection bias in case-control studies using an automated database	1997	[64]	term “graph” is used for something different than for patient graphs	graph as visualization	
**Faeder, James R.; Blinov, Michael L.; Hlavacek, William S.**	Graphical Rule-based Representation of Signal-transduction Networks	2005	[65]	graph is not associated to an individual patient	graph as visualization	
**Faloutsos, Christos**	Future Directions in Data Mining: Streams, Networks, Self-similarity and Power Laws	2002	[66]	graph is not associated to an individual patient	no patient data	
**Faloutsos, Christos; Kamel, Ibrahim**	Beyond Uniformity and Independence: Analysis of R-trees Using the Concept of Fractal Dimension	1994	[67]	term “graph” is used for something different than for patient graphs	no patient data	
**Fan, Kai; Eisenberg, Marisa; Walsh, Alison; Aiello, Allison; Heller, Katherine**	Hierarchical Graph-Coupled HMMs for Heterogeneous Personalized Health Data	2015	[68]	term “graph” is used for something different than for patient graphs	graph not used for modelling patient data	
**Farfan, F.; Hristidis, V.; Ranganathan, A.; Burke, R. P.**	Ontology-Aware Search on XML-based Electronic Medical Records	2008	[69]	graph is not associated to an individual patient	No graph theory	

**Fazeli Dehkordy, Soudabeh; Carlos, Ruth C.; Hall, Kelli S.; Dalton, Vanessa K.**	Novel data sources for women's health research: mapping breast screening online information seeking through Google trends	2014	[70]	term "graph" is used for something different than for patient graphs	graph as visualization	
**Feng, Jianxing; Zhu, Daming**	Faster Algorithms for Sorting by Transpositions and Sorting by Block Interchanges	2007	[71]	term "graph" is used for something different than for patient graphs	no patient data	
**Ferreira, Tania S.; Luiz de Souza-Braga, Andre; Soraia Cavalcanti-Valente, Geilsa; Ferreira de Souza, Deise; Moreira Carvalho-Alves, Enilda**	Nursing audit: The impact of nursing annotation in the context of hospital gloss	2009	[72]	wrong language	graph as visualization	
**Finlayson, Samuel G.; LePendu, Paea; Shah, Nigam H.**	Building the graph of medicine from millions of clinical narratives	2014	[73]	term "graph" is used for something different than for patient graphs	no data of individual patients	
**Flack, J. R.**	Seven years experience with a computerized diabetes clinic database	1995	[74]	term "graph" is used for something different than for patient graphs	graph as visualization	
**Florit, A. M.; Mori, A. R.; Simone, M. de; d'Annunzio, V.; Ricci, F. L.; Lalle, C.**	Context trees: representing co-operative healthcare activities in IREP	1995	[75]	graph is not associated to an individual patient	graph as visualization	
**Folio, Les R.; Machado, Laura B.; Dwyer, Andrew J.**	Multimedia-enhanced Radiology Reports: Concept, Components, and Challenges	2018	[76]	term "graph" is used for something different than for patient graphs	Graph as image	
**Forister, Julie F. Y.; Sun, Amy; Weissman, Barry A.**	Progress report on a post-radial keratotomy patient 20 years after surgery	2007	[77]	term "graph" is used for something different than for patient graphs	graph as visualization	
**Fors, U. G.; Sandberg, H. C.**	Computer-aided risk management--a software tool for the Hidep model	2001	[78]	term "graph" is used for something different than for patient graphs	graph as visualization	
**Fraccaro, Paolo; Vigo, Markel; Balatsoukas, Panagiotis; van der Veer, Sabine N.; Hassan, Lamiece; Williams, Richard; Wood, Grahame; Sinha, Smeeta; Buchan, Iain; Peek, Niels**	Presentation of laboratory test results in patient portals: influence of interface design on risk interpretation and visual search behaviour	2018	[79]	term "graph" is used for something different than for patient graphs	Graph as image	
**Friesen, Marcia R.; Hamel, Carole; McLeod, Robert D.**	A mHealth application for chronic wound care: findings of a user trial	2013	[80]	term "graph" is used for something different than for patient graphs	Graph as image	
**Gail, Mitchell H.; Costantino, Joseph P.; Pee, David; Bondy, Melissa; Newman, Lisa; Selvan, Mano; Anderson, Garnet L.; Malone, Kathleen E.; Marchbanks, Polly A.; McCaskill-Stevens, Worta; Norman, Sandra A.; Simon, Michael S.; Spirtas, Robert; Ursin, Giske; Bernstein, Leslie**	Projecting individualized absolute invasive breast cancer risk in African American women	2007	[81]	term "graph" is used for something different than for patient graphs	Graph as image	

**Galopin, Alexandre; Bouaud, Jacques; Pereira, Suzanne; Seroussi, Brigitte**	Using an ontological modeling to evaluate the consistency of clinical practice guidelines: application to the comparison of three guidelines on the management of adult hypertension	2014	[82]	term “graph” is used for something different than for patient graphs	no patient data but medcial guidelines modelled	
**Galopin, Alexandre; Bouaud, Jacques; Pereira, Suzanne; Seroussi, Brigitte**	An Ontology-Based Clinical Decision Support System for the Management of Patients with Multiple Chronic Disorders	2015	[83]	term “graph” is used for something different than for patient graphs	no patient data but medcial guidelines modelled	
**Garla, Vijay; Taylor, Caroline; Brandt, Cynthia**	Semi-supervised clinical text classification with Laplacian SVMs: an application to cancer case management	2013	[84]	term “graph” is used for something different than for patient graphs	No graph theory	
**Garrett, William F.; Fuchs, Henry; Whitton, Mary C.; State, Andrei**	Real-time Incremental Visualization of Dynamic Ultrasound Volumes Using Parallel BSP Trees	1996	[85]	term “graph” is used for something different than for patient graphs	No graph theory mentioned	
**Gevins, A. S.; Yeager, C. L.; Diamond, S. L.**	Interactive Analysis and Display of the Electroencephalogram (EEG) in Real Time	1974	[86]	term “graph” is used for something different than for patient graphs	No graph theory but signal processing	
**Gibson, Todd A.; Goldberg, Debra S.**	Evaluating Theoretical Models of Protein Interaction Network Evolution Without Seed Graphs	2013	[87]	term “graph” is used for something different than for patient graphs	no data of individual patients	
**Glinkowski, Wojciech**	Web-Based Support for Fracture Healing Evaluation and Monitoring	2011	[88]	term “graph” is used for something different than for patient graphs	Graph as image	
**Goodwin, Travis; Harabagiu, Sanda**	Embedding Open-domain Common-sense Knowledge from Text	2016	[89]	graph is not associated to an individual patient	no data of individual patients	
**Grinspan, Zachary M.; Abramson, Erika L.; Banerjee, Samprit; Kern, Lisa M.; Kaushal, Rainu; Shapiro, Jason S.**	Potential value of health information exchange for people with epilepsy: crossover patterns and missing clinical data	2013	[90]	term “graph” is used for something different than for patient graphs	No graph theory mentioned	
**Gunter, Carl**	Detecting Roles and Anomalies in Hospital Access Audit Logs	2014	[91]	term “graph” is used for something different than for patient graphs	no data of individual patients but audit logs	
**Guzzi, Pietro Hiram; Mina, Marco**	Towards the Assessment of Semantic Similarity Analysis of Protein Data: Main Approaches and Issues	2012	[92]	term “graph” is used for something different than for patient graphs	no data of individual patients	
**Hackney, David N.; Knudtson, Eric J.; Rossi, Karen Q.; Krugh, Dave; O'Shaughnessy, Richard W.**	Management of pregnancies complicated by anti-c isoimmunization	2004	[93]	term “graph” is used for something different than for patient graphs	graph as visualization	
**Halachev, Mihail; Shiri, Nematollaah; Thamildurai, Anand**	Exact Match Search in Sequence Data Using Suffix Trees	2005	[94]	graph is not associated to an individual patient	no data of individual patients	
**Harsola, A.; Thale, S.; Panse, M. S.**	Low Cost Digital Stethoscope for Heart Sounds	2011	[95]	term “graph” is used for something different than for patient graphs	Graph as image	

**Hartzler, Andrea L.; Izard, Jason P.; Dalkin, Bruce L.; Mikles, Sean P.; Gore, John L.**	Design and feasibility of integrating personalized PRO dashboards into prostate cancer care	2016	[96]	term "graph" is used for something different than for patient graphs	graph as visualization	
**Hausler, R.; Washburn, D.; Rey, P.; Stefanoff, F.**	Acquiring otoneurological results by computer. Evaluation after 2 years of experience	1982	[97]	term "graph" is used for something different than for patient graphs	French	
**Henry, S. B.; Mead, C. N.**	Nursing classification systems: necessary but not sufficient for representing "what nurses do" for inclusion in computer-based patient record systems	1997	[98]	term "graph" is used for something different than for patient graphs	nursing activities, not patient data	
**Henzinger, Monika Rauch; King, Valerie; Warnow, Tandy**	Constructing a Tree from Homeomorphic Subtrees, with Applications to Computational Evolutionary Biology	1996	[99]	term "graph" is used for something different than for patient graphs	no data of individual patients	
**Herman, Ted; Pemmaraju, Sriram V.; Segre, Alberto M.; Polgreen, Philip M.; Curtis, Donald E.; Fries, Jason; Hlady, Chris; Severson, Monica**	Wireless Applications for Hospital Epidemiology	2009	[100]	term "graph" is used for something different than for patient graphs	No graph theory	
**Hettne, Kristina; Soiland-Reyes, Stian; Klyne, Graham; Belhajjame, Khalid; Gamble, Matthew; Bechhofer, Sean; Roos, Marco; Corcho, Oscar**	Workflow Forever: Semantic Web Semantic Models and Tools for Preserving and Digitally Publishing Computational Experiments	2012	[101]	term "graph" is used for something different than for patient graphs	no data of individual patients but process graphs	
**Hofer, Christine; Kiechl, Stefan; Lang, Wilfried**	The Austrian Stroke-Unit-Registry	2008	[102]	term "graph" is used for something different than for patient graphs	graph as visualization	
**Hoimyr, Hilde; Christensen, Thomas D.; Emmertsen, Kristian; Johnsen, Soren P.; Riis, Anders; Hansen, Ole Kromann; Hjortdal, Vibeke E.**	Surgical repair of coarctation of the aorta: up to 40 years of follow-up	2006	[103]	term "graph" is used for something different than for patient graphs	graph as visualization	
**Hripcsak, George; Soulakis, Nicholas D.; Li, Li; Morrison, Frances P.; Lai, Albert M.; Friedman, Carol; Calman, Neil S.; Mostashari, Farzad**	Syndromic surveillance using ambulatory electronic health records	2009	[104]	term "graph" is used for something different than for patient graphs	graph as visualization	
**Huang, H.; Gong, T.; Ye, N.; Wang, R.; Dou, Y.**	Private and Secured Medical Data Transmission and Analysis for Wireless Sensing Healthcare System	2017	[105]	term "graph" is used for something different than for patient graphs	No graph theory mentioned	
**Huesch, Marco D.; Schetter, Susann; Segel, Joel; Chetlen, Alison**	Evaluation of the "Angelina Jolie Effect" on Screening Mammography Utilization in an Academic Center	2017	[106]	term "graph" is used for something different than for patient graphs	no data of individual patients	
**Islam, S. K. Hafizul; Khan, Muhammad Khurram; Li, Xiong**	Security Analysis and Improvement of 'a More Secure Anonymous User Authentication Scheme for the Integrated EPR Information System'	2015	[107]	term "graph" is used for something different than for patient graphs	No graph mentioned	

	Italian cancer figures, report 2012: Cancer in children and adolescents	2013	[108]	term “graph” is used for something different than for patient graphs	term “graph” is used for something different than for patient graphs	
**Jacky, Jonathan P.; Kalet, Ira J.**	An Object-oriented Programming Discipline for Standard Pascal	1987	[109]	term “graph” is used for something different than for patient graphs	no medical context	
**Jamakovic, Mesud; Baljic, Rusmir**	Significance of copper level in serum and routine laboratory parameters in estimation of outspreading of Hodgkin's lymphoma	2013	[110]	term “graph” is used for something different than for patient graphs	graph as visualization	
**Jamil, Hasan M.; Modica, Giovanni A.; Teran, Maria A.**	Towards a Visual Query Interface for Phylogenetic Databases	2001	[111]	term “graph” is used for something different than for patient graphs	no data of individual patients	
**Jamison, Robert N.; Jurcik, Dylan C.; Edwards, Robert R.; Huang, Chuan-Chin; Ross, Edgar L.**	A Pilot Comparison of a Smartphone App With or Without 2-Way Messaging Among Chronic Pain Patients: Who Benefits From a Pain App?	2017	[112]	term “graph” is used for something different than for patient graphs	graph as visualization	
**Kawada, T.; Aoki, S.; Suzuki, S.**	Management of personal health examination data for a population by use of a portable computer	1992	[113]	term “graph” is used for something different than for patient graphs	graph as visualization	
**Kay, Matthew; Choe, Eun Kyoung; Shepherd, Jesse; Greenstein, Benjamin; Watson, Nathaniel; Consolvo, Sunny; Kientz, Julie A.**	Lullaby: A Capture & Access System for Understanding the Sleep Environment	2012	[114]	term “graph” is used for something different than for patient graphs	graph as visualization	
**Kazmierczak, Steven C.; Leen, Todd K.; Erdogmus, Deniz; Carreira-Perpinan, Miguel A.**	Reduction of multi-dimensional laboratory data to a two-dimensional plot: a novel technique for the identification of laboratory error	2007	[115]	term “graph” is used for something different than for patient graphs	graph as visualization	
**Keenan, Gail M.; Lopez, Karen Dunn; Yao, Yingwei; Sousa, Vanessa E. C.; Stifter, Janet; Febretti, Alessandro; Johnson, Andrew; Wilkie, Diana J.**	Toward Meaningful Care Plan Clinical Decision Support: Feasibility and Effects of a Simulated Pilot Study	2017	[116]	term “graph” is used for something different than for patient graphs	graph as visualization	
**Kennedy, I.; Ritter, H.**	Antenatal records: do they help us? A new record for watching fetal growth	1984	[117]	term “graph” is used for something different than for patient graphs	graph as visualization	
**Kennedy, I.; Stephens, B.**	A novel antenatal record to help midwives	1979	[118]	term “graph” is used for something different than for patient graphs	graph as visualization	
**Khan, I. A.**	Personalized Electronic Health Record System for Monitoring Patients with Chronic Disease	2013	[119]	term “graph” is used for something different than for patient graphs	graph as visualization	
**Khan, Arijit; Bhowmick, Sourav S.; Bonchi, Francesco**	Summarizing Static and Dynamic Big Graphs	2017	[120]	graph is not associated to an individual patient	no graph representation of data but graph reduction	

**Khan, Arijit; Elnikety, Sameh**	Systems for Big-graphs	2014	[121]	graph is not associated to an individual patient	no data of individual patients	
**Klann, Jeffrey G.; McCoy, Allison B.; Wright, Adam; Wattanasin, Nich; Sittig, Dean F.; Murphy, Shawn N.**	Health care transformation through collaboration on open-source informatics projects: integrating a medical applications platform, research data repository, and patient summarization	2013	[122]	term "graph" is used for something different than for patient graphs	graph as visualization	
**Klann, Jeffrey G.; Szolovits, Peter; Downs, Stephen M.; Schadow, Gunther**	Decision support from local data: creating adaptive order menus from past clinician behavior	2014	[123]	graph is not associated to an individual patient	No graph theory	
**Kofler, J.; Pesenhofer, R.; Landl, G.; Sommerfeld-Stur, I.; Peham, C.**	Monitoring of dairy cow claw health status in 15 herds using the computerised documentation program Claw Manager and digital parameters	2013	[124]	term "graph" is used for something different than for patient graphs	graph as visualization	
**Kourtis, Georgios; Caiaffa, Maria-Filomena; Forte, Cesarea; Scarlato, Marisa I.; Macchia, Luigi**	Retrospective monitoring in the management of persistent asthma	2011	[125]	term "graph" is used for something different than for patient graphs	graph as visualization	
**Kraus, Stefan; Castellanos, Ixchel; Albermann, Matthias; Schuettler, Christina; Prokosch, Hans-Ulrich; Staudigel, Martin; Toddenroth, Dennis**	Using Arden Syntax for the Generation of Intelligent Intensive Care Discharge Letters	2016	[126]	term "graph" is used for something different than for patient graphs	Graph as image	
**Kushniruk, Andre; Borycki, Elizabeth; Anderson, James G.; Anderson, Marilyn M.**	Combining Two Forms of Simulation to Predict the Potential Impact of Interface Design on Technology-induced Error in Healthcare	2008	[127]	graph is not associated to an individual patient	graph as visualization	
**Kwon, Yongjin; Kang, Kyuchang; Bae, Changseok; Chung, Hee-Joon; Kim, Ju Han**	Lifelog agent for human activity pattern analysis on health avatar platform	2014	[128]	term "graph" is used for something different than for patient graphs	no medical data but activity graphs	
**Labarre, Anthony; Verwer, Sicco**	Merging Partially Labelled Trees: Hardness and a Declarative Programming Solution	2014	[129]	graph is not associated to an individual patient	no data of individual patients	
**Lancia, Giuseppe; Carr, Robert; Walenz, Brian; Istrail, Sorin**	101 Optimal PDB Structure Alignments: A Branch-and-cut Algorithm for the Maximum Contact Map Overlap Problem	2001	[130]	graph is not associated to an individual patient	no data of individual patients	
**Landes, Sara J.; Carlson, Eve B.; Ruzek, Josef I.; Wang, Dan; Hugo, Emily; DeGaetano, Noah; Chambers, Justin G.; Lindley, Steven E.**	Provider-Driven Development of a Measurement Feedback System to Enhance Measurement-Based Care in VA Mental Health	2015	[131]	term "graph" is used for something different than for patient graphs	graph as visualization	
**Lee, Hee Jun; Kim, Ju Yeong; Kim, Seul Ki; Lee, Jung Ryeol; Suh, Chang Suk; Kim, Seok Hyun**	Learning Curve Analysis and Surgical Outcomes of Single-port Laparoscopic Myomectomy	2015	[132]	term "graph" is used for something different than for patient graphs	graph as visualization	
**Levin, Roger J.**	Incidence of thyroid cancer in residents surrounding the Three Mile Island nuclear facility	2008	[133]	term "graph" is used for something different than for patient graphs	graph as visualization	

**Lezcano, Leonardo; Sanchez-Alonso, Salvador; Sicilia, Miguel-Angel**	Associating clinical archetypes through UMLS Metathesaurus term clusters	2012	[134]	graph is not associated to an individual patient	no data of individual patients	
**Liabsuetrakul, Tippawan; Prappre, Tagoon; Pairot, Pakamas; Oumudee, Nurlisa; Islam, Monir**	Development of a web-based epidemiological surveillance system with health system response for improving maternal and newborn health: Field-testing in Thailand	2017	[135]	term “graph” is used for something different than for patient graphs	Graph as image	
**Lin, Ying Ling; Guerguerian, Anne-Marie; Tomasi, Jessica; Laussen, Peter; Trbovich, Patricia**	Usability of data integration and visualization software for multidisciplinary pediatric intensive care: a human factors approach to assessing technology	2017	[136]	term “graph” is used for something different than for patient graphs	graph as visualization	
**Lin, Hsiu-Wen; Wang, Yu-Jen; Jing, Ling-Fang; Chang, Polun**	Mockup design of personal health diary app for patients with chronic kidney disease	2014	[137]	term “graph” is used for something different than for patient graphs	graph as visualization	
**Liu, Wei; Chung, Bo Chuen; Wang, Rui; Ng, Jonathon; Morlet, Nigel**	A genetic algorithm enabled ensemble for unsupervised medical term extraction from clinical letters	2015	[138]	graph is not associated to an individual patient	term “graph” is used for something different than for patient graphs	
**Liu, H.; Dou, D.; Jin, R.; Lependu, P.; Shah, N.**	Mining Biomedical Ontologies and Data Using RDF Hypergraphs	2013	[139]	term “graph” is used for something different than for patient graphs	term “graph” is used for something different than for patient graphs	
**Liu, Yifeng; Guo, Zhaochen; Ke, Xiaodi; Za\"ıane, Osmar R.**	Protein Subcellular Localization Prediction with Associative Classification and Multi-class SVM	2011	[140]	term “graph” is used for something different than for patient graphs	term “graph” is used for something different than for patient graphs	
**Liu, J. M.; Wu, H. W.; Chen, W. S.; Lin, W. C.; Chao, Y.; Lui, W. Y.; Whang-Peng, J.**	Integration of computer-assembled digital images and text data as evidence for the oncological record	2000	[141]	term “graph” is used for something different than for patient graphs	term “graph” is used for something different than for patient graphs	
**Loorak, Mona Hosseinkhani; Perin, Charles; Kamal, Noreen; Hill, Michael; Carpendale, Sheelagh**	TimeSpan: Using Visualization to Explore Temporal Multi-dimensional Data of Stroke Patients	2016	[142]	graph is not associated to an individual patient	term “graph” is used for something different than for patient graphs	
**Lopez, Karen Dunn; Wilkie, Diana J.; Yao, Yingwei; Sousa, Vanessa; Febretti, Alessandro; Stifter, Janet; Johnson, Andrew; Keenan, Gail M.**	Nurses' Numeracy and Graphical Literacy: Informing Studies of Clinical Decision Support Interfaces	2016	[143]	term “graph” is used for something different than for patient graphs	term “graph” is used for something different than for patient graphs	
**Lovis, C.; Baud, R. H.; Revillard, C.; Pult, L.; Borst, F.; Geissbuhler, A.**	Paragraph-oriented structure for narratives in medical documentation	2001	[144]	term “graph” is used for something different than for patient graphs	term “graph” is used for something different than for patient graphs	
**Luo, Yuan; Xin, Yu; Hochberg, Ephraim; Joshi, Rohit; Uzuner, Ozlem; Szolovits, Peter**	Subgraph augmented non-negative tensor factorization (SANTF) for modeling clinical narrative text	2015	[145]	term “graph” is used for something different than for patient graphs	term “graph” is used for something different than for patient graphs	
**Luque-Fernandez, Miguel Angel; Zoega, Helga; Valdimarsdottir, Unnur; Williams, Michelle A.**	Deconstructing the smoking-preeclampsia paradox through a counterfactual framework	2016	[146]	term “graph” is used for something different than for patient graphs	term “graph” is used for something different than for patient graphs	

**Madkour, M.; Song, Hsing-yi; Du Jingcheng; Tao, C.**	A representational analysis of a temporal indeterminancy display in clinical events	2016	[147]	term “graph” is used for something different than for patient graphs	term “graph” is used for something different than for patient graphs	
**Mandal, A. K.; Sarkar, A.**	Formal representation of service interactions for SaaS based applications	2014	[148]	term “graph” is used for something different than for patient graphs	term “graph” is used for something different than for patient graphs	
**Margusino-Framinan, Luis; Cid-Silva, Purificacion; Mena-de-Cea, Alvaro; Sanclaudio-Luhia, Ana Isabel; Castro-Castro, Jose Antonio; Vazquez-Gonzalez, Guillermo; Martin-Herranz, Isabel**	Intelligent MONitoring System for antiviral pharmacotherapy in patients with chronic hepatitis C (SiMON-VC)	2017	[149]	term “graph” is used for something different than for patient graphs	term “graph” is used for something different than for patient graphs	
**Markazi-Moghaddam, Nader; Arab, Mohammad; Ravaghi, Hamid; Rashidian, Arash; Khatibi, Toktam; Zargar Balaye Jame, Sanaz**	A Knowledge Map for Hospital Performance Concept: Extraction and Analysis: A Narrative Review Article	2016	[150]	term “graph” is used for something different than for patient graphs	term “graph” is used for something different than for patient graphs	
**Martin, Gina S.; Tapsell, Linda C.; Batterham, Marijka J.; Russell, Kenneth G.**	Relative bias in diet history measurements: a quality control technique for dietary intervention trials	2002	[151]	graph is not associated to an individual patient	term “graph” is used for something different than for patient graphs	
**Martinez, David; Otegi, Arantxa; Soroa, Aitor; Agirre, Eneko**	Improving search over Electronic Health Records using UMLS-based query expansion through random walks	2014	[152]	term “graph” is used for something different than for patient graphs	term “graph” is used for something different than for patient graphs	
**Martinez-Costa, Catalina; Schulz, Stefan**	Validating EHR clinical models using ontology patterns	2017	[153]	term “graph” is used for something different than for patient graphs	term “graph” is used for something different than for patient graphs	
**Matthews, Suzanne J.**	Heterogeneous Compression of Large Collections of Evolutionary Trees	2015	[154]	term “graph” is used for something different than for patient graphs	term “graph” is used for something different than for patient graphs	
**Mechkour, M.; Mulhem, P.; Fourel, F.; Berrut, E. F. C.**	PRIME-GC. A medical information retrieval prototype on the Web	1997	[155]	term “graph” is used for something different than for patient graphs	term “graph” is used for something different than for patient graphs	
**Mendonça, Eneida A.; Johnson, Stephen B.; Seol, Yoon-Ho; Cimino, James J.**	Analyzing the Semantics of Patient Data to Rank Records of Literature Retrieval	2002	[156]	term “graph” is used for something different than for patient graphs	term “graph” is used for something different than for patient graphs	
**Meystre, Stephane M.; Lee, Sanghoon; Jung, Chai Young; Chevrier, Raphael D.**	Common data model for natural language processing based on two existing standard information models: CDA+GrAF	2012	[157]	term “graph” is used for something different than for patient graphs	term “graph” is used for something different than for patient graphs	
**Milicchio, Franco; Tradigo, Giuseppe; Veltri, Pierangelo; Prosperi, Mattia**	High-performance Data Structures for De Novo Assembly of Genomes: Cache Oblivious Generic Programming	2016	[158]	term “graph” is used for something different than for patient graphs	term “graph” is used for something different than for patient graphs	
**Mirshahi, A.; Kohnen, T.**	Scientific evaluation and quality assurance in refractive surgical interventions. Evaluation of the Datagraph med computer program	2002	[159]	term “graph” is used for something different than for patient graphs	term “graph” is used for something different than for patient graphs	

**Mirzaei, Masoud; Truswell, A. Stewart; Arnett, Kathryn; Page, Andrew; Taylor, Richard; Leeder, Stephen R.**	Cerebrovascular disease in 48 countries: secular trends in mortality 1950-2005	2012	[160]	term "graph" is used for something different than for patient graphs	term "graph" is used for something different than for patient graphs	
**Morin, Patricia; Herrmann, Francois; Ammann, Patrick; Uebelhart, Brigitte; Rizzoli, Rene**	A rapid self-administered food frequency questionnaire for the evaluation of dietary protein intake	2005	[161]	graph is not associated to an individual patient	term "graph" is used for something different than for patient graphs	
**Moruzzi, Mauro**	The new culture of dematerialized health	2014	[162]	term "graph" is used for something different than for patient graphs	term "graph" is used for something different than for patient graphs	
**Mossel, Elchanan; Roch, Sebastien; Steel, Mike**	Shrinkage Effect in Ancestral Maximum Likelihood	2009	[163]	term "graph" is used for something different than for patient graphs	term "graph" is used for something different than for patient graphs	
**Mozhayskiy, Vadim; Miller, Bob; Ma, Kwan-Liu; Tagkopoulos, Ilias**	A Scalable Multi-scale Framework for Parallel Simulation and Visualization of Microbial Evolution	2011	[164]	graph is not associated to an individual patient	graph is not associated to an individual patient	
**Muller, R.**	The CliniCon framework for context representation in electronic patient records	1997	[165]	term "graph" is used for something different than for patient graphs	term "graph" is used for something different than for patient graphs	
**Murphy, Cynthia A.; Carstens, Kimberly; Villamayor, Precy**	Electronic growth charts: watching our patients grow	2005	[166]	term "graph" is used for something different than for patient graphs	term "graph" is used for something different than for patient graphs	
**Mwaniki, Paul; Ayieko, Philip; Todd, Jim; English, Mike**	Assessment of paediatric inpatient care during a multifaceted quality improvement intervention in Kenyan district hospitals--use of prospectively collected case record data	2014	[167]	term "graph" is used for something different than for patient graphs	term "graph" is used for something different than for patient graphs	
**Myleus, Anna; Stenlund, Hans; Hernell, Olle; Gothefors, Leif; Hammarstrom, Marie-Louise; Persson, Lars-Ake; Ivarsson, Anneli**	Early vaccinations are not risk factors for celiac disease	2012	[168]	graph is not associated to an individual patient	graph is not associated to an individual patient	
**Nagar, Anurag; Al-Mubaid, Hisham; Bettayeb, Said**	Computing Gene Functional Similarity Using Combined Graphs	2012	[169]	term "graph" is used for something different than for patient graphs	term "graph" is used for something different than for patient graphs	
**Newell, S. D. [JR]; Englert, J.; Box-Taylor, A.; Davis, K. M.; Koch, K. E.**	Clinical efficiency tools improve stroke management in a rural southern health system	1998	[170]	graph is not associated to an individual patient	term "graph" is used for something different than for patient graphs	
**Ng, Kenney; Ghoting, Amol; Steinhubl, Steven R.; Stewart, Walter F.; Malin, Bradley; Sun, Jimeng**	PARAMO: a PARAllel predictive MOdeling platform for healthcare analytic research using electronic health records	2014	[171]	graph is not associated to an individual patient	term "graph" is used for something different than for patient graphs	
**Nielsen, Finn \AArup**	A Fielded Wiki for Personality Genetics	2010	[172]	term "graph" is used for something different than for patient graphs	term "graph" is used for something different than for patient graphs	

**Nikfarjam, Azadeh; Emadzadeh, Ehsan; Gonzalez, Graciela**	Towards generating a patient's timeline: extracting temporal relationships from clinical notes	2013	[173]	term “graph” is used for something different than for patient graphs	term “graph” is used for something different than for patient graphs	
**Nori, Nozomi; Kashima, Hisashi; Yamashita, Kazuto; Ikai, Hiroshi; Imanaka, Yuichi**	Simultaneous Modeling of Multiple Diseases for Mortality Prediction in Acute Hospital Care	2015	[174]	term “graph” is used for something different than for patient graphs	term “graph” is used for something different than for patient graphs	
**Okamoto, K.; Tanaka, H.; Takemura, T.; Kume, N.; Kuroda, T.; Yoshihara, H.**	A hypothesis-generating support system using medical records for clinical knowledge acquisition	2012	[175]	term “graph” is used for something different than for patient graphs	term “graph” is used for something different than for patient graphs	
**Orban, Kristina; Edberg, Anna-Karin; Erlandsson, Lena-Karin**	Using a time-geographical diary method in order to facilitate reflections on changes in patterns of daily occupations	2012	[176]	term “graph” is used for something different than for patient graphs	term “graph” is used for something different than for patient graphs	
**O'Sullivan, E. M.**	International variation in the incidence of oral and pharyngeal cancer	2008	[177]	term “graph” is used for something different than for patient graphs	term “graph” is used for something different than for patient graphs	
**Pabón, M. C.; Montoya, G. A.; Millán, M.**	Mediation and graph data models for medical data integration	2013	[178]	wrong language	no Eng	
**Parkin, D. Max; Ferlay, Jacques; Curado, Maria-Paula; Bray, Freddie; Edwards, Brenda; Shin, Hai-Rim; Forman, David**	Fifty years of cancer incidence: CI5 I-IX	2010	[179]	term “graph” is used for something different than for patient graphs	term “graph” is used for something different than for patient graphs	
**Patil, R.; Karandikar, R. G.**	Digital signal preservation approaches of archived biomedical paper records #x2014; A review	2016	[180]	Dublette	Dublette	
**Pawloski, Pamala; Cusick, Dona; Amborn, Lori**	Development of clinical pharmacy productivity metrics	2012	[181]	term “graph” is used for something different than for patient graphs	term “graph” is used for something different than for patient graphs	
**Payne, T. H.; Andrews, R. D.; Breeling, J.; Ben Davoren, J.; Smith, R. M.; Volpp, B.**	Graphing clinical events in the VA Computerized Patient Record System	1999	[182]	term “graph” is used for something different than for patient graphs	term “graph” is used for something different than for patient graphs	
**Pedreschi, Dino**	Social Network Analytics, Data Science Ethics & Privacy-preserving Analytics	2017	[183]	term “graph” is used for something different than for patient graphs	term “graph” is used for something different than for patient graphs	
**Penfield, W.**	Physicians take to the field	1992	[184]	term “graph” is used for something different than for patient graphs	term “graph” is used for something different than for patient graphs	
**Pfeiffer, Karl P.**	Actual state and perspectives of e-health in Austria and international--an overview	2011	[185]	term “graph” is used for something different than for patient graphs	term “graph” is used for something different than for patient graphs	
**Pfundner, Alexander; Schnoeberg, Tobias; Horn, John; Boyce, Richard D.; Samwald, Matthias**	Utilizing the Wikidata System to Improve the Quality of Medical Content in Wikipedia in Diverse Languages: A Pilot Study	2015	[186]	term “graph” is used for something different than for patient graphs	term “graph” is used for something different than for patient graphs	

**Poon-Chue, A.; Menendez, L.; Gerstner, M. M.; Colletti, P.; Terk, M.**	MRI evaluation of post-operative seromas in extremity soft tissue sarcomas	1999	[187]	term “graph” is used for something different than for patient graphs	term “graph” is used for something different than for patient graphs	
**Pourriat, J. L.; Huet, B.; Gabry, A. L.; Rolland, C.; Cupa, M.**	Case summaries in real-time. Accomplishments with a microcomputer	1982	[188]	term “graph” is used for something different than for patient graphs	term “graph” is used for something different than for patient graphs	
**Poursadegh, Mehdi; Poursadegh, Farid; Esmaeili, Majid; Bakhshaee, Mehdi**	Epidemiological Survey of Sinonasal Malignancy in North-East Iran	2015	[189]	graph is not associated to an individual patient	graph is not associated to an individual patient	
**Powsner, S. M.; Tufte, E. R.**	Summarizing clinical psychiatric data	1997	[190]	term “graph” is used for something different than for patient graphs	term “graph” is used for something different than for patient graphs	
**Preece, Megan H. W.; Hill, Andrew; Horswill, Mark S.; Karamatic, Rozemary; Watson, Marcus O.**	Designing observation charts to optimize the detection of patient deterioriation: reliance on the subjective preferences of healthcare professionals is not enough	2012	[191]	term “graph” is used for something different than for patient graphs	term “graph” is used for something different than for patient graphs	
**Pryor, Julie**	A snapshot of rehabilitation referrals in rural New South Wales	2010	[192]	term “graph” is used for something different than for patient graphs	term “graph” is used for something different than for patient graphs	
**Rajagopalan, M. R.; Vellaipandiyan, S.**	Big data framework for national E-governance plan	2013	[193]	term “graph” is used for something different than for patient graphs	term “graph” is used for something different than for patient graphs	
**Rakic, N.**	Acute sensorineural hearing loss at the Otorhinolaryngology Department of the General Hospital in Subotica 1991-1996	1999	[194]	graph is not associated to an individual patient	term “graph” is used for something different than for patient graphs	
**RAMAYYA, G. P.**	AXAUDIT - ANESTHETIC AUDIT SYSTEM	1992	[195]	term “graph” is used for something different than for patient graphs	term “graph” is used for something different than for patient graphs	
**Rassinoux, A. M.; Michel, P. A.; Wagner, J.; Baud, R.**	Current trends with natural language processing	1995	[196]	term “graph” is used for something different than for patient graphs	term “graph” is used for something different than for patient graphs	
**Ratib, O.**	From multimodality digital imaging to multimedia patient record	1994	[197]	wrong language	term “graph” is used for something different than for patient graphs	
**Reiz, Beata; Csato, Lehel**	Bayesian Network Classifier for Medical Data Analysis	2009	[198]	term “graph” is used for something different than for patient graphs	term “graph” is used for something different than for patient graphs	
**Rhea, Sarah; Weber, David J.; Poole, Charles; Cairns, Charles**	Risk factors for hospitalization after dog bite injury: a case-cohort study of emergency department visits	2014	[199]	term “graph” is used for something different than for patient graphs	term “graph” is used for something different than for patient graphs	
**Rizvi, Syed Zain R.; Fong, Philip W.L.**	Interoperability of Relationship- and Role-Based Access Control	2016	[200]	term “graph” is used for something different than for patient graphs	term “graph” is used for something different than for patient graphs	

**Rizvi, Syed Zain R.; Fong, Philip W.L.; Crampton, Jason; Sellwood, James**	Relationship-Based Access Control for an Open-Source Medical Records System	2015	[201]	term “graph” is used for something different than for patient graphs	term “graph” is used for something different than for patient graphs	
**Rocha, J.**	Graph Comparison by Log-Odds Score Matrices with Application to Protein Topology Analysis	2011	[202]	term “graph” is used for something different than for patient graphs	term “graph” is used for something different than for patient graphs	
**Ruiz, Jorge G.; Andrade, Allen D.; Hogue, Christie; Karanam, Chandana; Akkineni, Sisir; Cevallos, David; Anam, Ramanakumar; Sharit, Joseph**	The Association of Graph Literacy With Use of and Skills Using an Online Personal Health Record in Outpatient Veterans	2016	[203]	graph is not associated to an individual patient	graph is not associated to an individual patient	
**Ryu, Ini; Siio, Itiro**	TongueDx: A Tongue Diagnosis for Health Care on Smartphones	2014	[204]	graph is not associated to an individual patient	term “graph” is used for something different than for patient graphs	
**Salinas, Oscar; Cosio, Gerardo de; Clavel-Arcas, Carme; Montoya, Jeannette; Serpas, Mario; Moran de Garcia, Silvia; Concha-Eastman, Alberto**	An information system for injuries from external causes (SILEX): a successful project in El Salvador	2008	[205]	term “graph” is used for something different than for patient graphs	term “graph” is used for something different than for patient graphs	
**Sauleau, Erik A.; Paumier, Jean-Philippe; Buemi, Antoine**	Medical record linkage in health information systems by approximate string matching and clustering	2005	[206]	graph is not associated to an individual patient	term “graph” is used for something different than for patient graphs	
**Schenk, Robert J. [JR]; Schenk, Jenna**	Integration of remote blood glucose meter upload technology into a clinical pharmacist medication therapy management service	2011	[207]	term “graph” is used for something different than for patient graphs	term “graph” is used for something different than for patient graphs	
**Schultz, S. E.; Rothwell, D. M.; Chen, Z.; Tu, K.**	Identifying cases of congestive heart failure from administrative data: a validation study using primary care patient records	2013	[208]	Dublette	Dublette	
**Schultz, S. E.; Rothwell, D. M.; Chen, Z.; Tu, K.**	Identifying cases of congestive heart failure from administrative data: a validation study using primary care patient records	2013	[209]	term “graph” is used for something different than for patient graphs	term “graph” is used for something different than for patient graphs	
**Sevick, Mary Ann; Zickmund, Susan; Korytkowski, Mary; Piraino, Beth; Sereika, Susan; Mihalko, Shannon; Snetselaar, Linda; Stumbo, Phyllis; Hausmann, Leslie; Ren, Dianxu; Marsh, Rita; Sakraida, Teresa; Gibson, Jolynn; Safaien, Mehry; Starrett, Terry J.; Burke, Lora E.**	Design, feasibility, and acceptability of an intervention using personal digital assistant-based self-monitoring in managing type 2 diabetes	2008	[210]	term “graph” is used for something different than for patient graphs	term “graph” is used for something different than for patient graphs	
**Sharit, Joseph; Lisigurski, Miriam; Andrade, Allen D.; Karanam, Chandana; Nazi, Kim M.; Lewis, James R.; Ruiz, Jorge G.**	The Roles of Health Literacy, Numeracy, and Graph Literacy on the Usability of the VA’s Personal Health Record by Veterans	2014	[211]	term “graph” is used for something different than for patient graphs	term “graph” is used for something different than for patient graphs	
**Shaverdian, Anna A.; Zhou, Hao; Michailidis, George; Jagadish, H. V.**	Algebraic Visual Analysis: The Catalano Phone Call Data Set Case Study	2009	[212]	graph is not associated to an individual patient	graph is not associated to an individual patient	

**Shaw, Julie L. V.; Cohen, Ashley; Konforte, Danijela; Binesh-Marvasti, Tina; Colantonio, David A.; Adeli, Khosrow**	Validity of establishing pediatric reference intervals based on hospital patient data: a comparison of the modified Hoffmann approach to CALIPER reference intervals obtained in healthy children	2014	[213]	graph is not associated to an individual patient	term “graph” is used for something different than for patient graphs	
**Sheridan, Joanna; Chamberlain, Kerry; Dupuis, Ann**	Timelining: visualizing experience	2011	[214]	term “graph” is used for something different than for patient graphs	term “graph” is used for something different than for patient graphs	
**Shibuya, Tetsuo**	Geometric Suffix Tree: Indexing Protein 3-D Structures	2010	[215]	term “graph” is used for something different than for patient graphs	term “graph” is used for something different than for patient graphs	
**Shigli, Heenakousar; Tejas, M. H.; Narayan, Lohit; Desai, Shrinivas D.**	User Intervention Based Segmentation of Myocardium In Cardiac Cine MRI Images	2015	[216]	term “graph” is used for something different than for patient graphs	term “graph” is used for something different than for patient graphs	
**Shim, S-H; Kim, D-Y; Lee, D-Y; Lee, S-W; Park, J-Y; Lee, J. J.; Kim, J-H; Kim, Y-M; Kim, Y-T; Nam, J-H**	Metabolic tumour volume and total lesion glycolysis, measured using preoperative 18F-FDG PET/CT, predict the recurrence of endometrial cancer	2014	[217]	term “graph” is used for something different than for patient graphs	term “graph” is used for something different than for patient graphs	
**Sills, Marion R.; Kwan, Bethany M.; Yawn, Barbara P.; Sauer, Brian C.; Fairclough, Diane L.; Federico, Monica J.; Juarez-Colunga, Elizabeth; Schilling, Lisa M.**	Medical home characteristics and asthma control: a prospective, observational cohort study protocol	2013	[218]	term “graph” is used for something different than for patient graphs	term “graph” is used for something different than for patient graphs	
**Sim, Livvi Li Wei; Ban, Kenneth Hon Kim; Tan, Tin Wee; Sethi, Sunil Kumar; Loh, Tze Ping**	Development of a clinical decision support system for diabetes care: A pilot study	2017	[219]	term “graph” is used for something different than for patient graphs	term “graph” is used for something different than for patient graphs	
**Sittig, Dean F.; Murphy, Daniel R.; Smith, Michael W.; Russo, Elise; Wright, Adam; Singh, Hardeep**	Graphical display of diagnostic test results in electronic health records: a comparison of 8 systems	2015	[220]	term “graph” is used for something different than for patient graphs	term “graph” is used for something different than for patient graphs	
**SMART, J. F.; ROUX, M.**	A MODEL FOR MEDICAL KNOWLEDGE REPRESENTATION APPLICATION TO THE ANALYSIS OF DESCRIPTIVE PATHOLOGY REPORTS	1995	[221]	term “graph” is used for something different than for patient graphs	term “graph” is used for something different than for patient graphs	
**Sobo, E. J.; Andriese, S.; Stroup, C.; Morgan, D.; Kurtin, P.**	Developing indicators for emergency medical services (EMS) system evaluation and quality improvement: a statewide demonstration and planning project	2001	[222]	term “graph” is used for something different than for patient graphs	term “graph” is used for something different than for patient graphs	
**Soldes, O. S.; Younger, J. G.; Hirschl, R. B.**	Predictors of malignancy in childhood peripheral lymphadenopathy	1999	[223]	term “graph” is used for something different than for patient graphs	term “graph” is used for something different than for patient graphs	
**Sondhi, Parikshit; Sun, Jimeng; Tong, Hanghang; Zhai, ChengXiang**	SympGraph: A Framework for Mining Clinical Notes Through Symptom Relation Graphs	2012	[224]	term “graph” is used for something different than for patient graphs	term “graph” is used for something different than for patient graphs	

**Sovik, Signe; Skaga, Nils Oddvar; Hanoa, Rolf; Eken, Torsten**	Sudden survival improvement in critical neurotrauma: An exploratory analysis using a stratified statistical process control technique	2014	[225]	graph is not associated to an individual patient	term "graph" is used for something different than for patient graphs	
**Sridhar, Srinath; Dhamdhere, Kedar; Blelloch, Guy; Halperin, Eran; Ravi, R.; Schwartz, Russell**	Algorithms for Efficient Near-Perfect Phylogenetic Tree Reconstruction in Theory and Practice	2007	[226]	term "graph" is used for something different than for patient graphs	term "graph" is used for something different than for patient graphs	
**Stefansson, C. G.**	Map analyses of psychiatric services. The application of a computerized psychiatric case register to geographical analysis	1984	[227]	term "graph" is used for something different than for patient graphs	term "graph" is used for something different than for patient graphs	
**Stevens, V. J.; Rossner, J.; Greenlick, M.; Stevens, N.; Frankel, H. M.; Craddick, S.**	Freedom from fat: a contemporary multi-component weight loss program for the general population of obese adults	1989	[228]	term "graph" is used for something different than for patient graphs	term "graph" is used for something different than for patient graphs	
**Sumner, W. 2nd; Truszczynski, M.; Marek, V. W.**	A formal model of family medicine	1996	[229]	term "graph" is used for something different than for patient graphs	term "graph" is used for something different than for patient graphs	
**Sun, Kai; Goncalves, Joana P.; Larminie, Chris; Przulj, Natasa**	Predicting disease associations via biological network analysis	2014	[230]	term "graph" is used for something different than for patient graphs	term "graph" is used for something different than for patient graphs	
**Swartz, Susan H.; Cowan, Timothy M.; DePue, Judy; Goldstein, Michael G.**	Academic profiling of tobacco-related performance measures in primary care	2002	[231]	term "graph" is used for something different than for patient graphs	term "graph" is used for something different than for patient graphs	
**Syalim, Amril; Nishide, Takashi; Sakurai, Kouichi**	Securing Provenance of Distributed Processes in an Untrusted Environment	2012	[232]	term "graph" is used for something different than for patient graphs	term "graph" is used for something different than for patient graphs	
**Szeto, Lap Keung; Liew, Alan Wee-Chung; Yan, Hong; Tang, Sy-sen**	Gene Expression Data Clustering and Visualization Based on a Binary Hierarchical Clustering Framework	2003	[233]	term "graph" is used for something different than for patient graphs	term "graph" is used for something different than for patient graphs	
**Taffe, John; Dennerstein, Lorraine**	Menstrual diary data and menopausal transition: methodologic issues	2002	[234]	term "graph" is used for something different than for patient graphs	term "graph" is used for something different than for patient graphs	
**Techentin, R.; Foti, D.; Li, P.; Daniel, E.; Gilbert, B.; Holmes, D.; Al-Saffar, S.**	Development of a Semi-synthetic Dataset as a Testbed for Big-Data Semantic Analytics	2014	[235]	term "graph" is used for something different than for patient graphs	term "graph" is used for something different than for patient graphs	
**Tezcan, B.; Khazaezadeh, N.; Ash, A.; Oteng-Ntim, E.**	Social disparity and intrauterine death: from politics to policies	2011	[236]	term "graph" is used for something different than for patient graphs	term "graph" is used for something different than for patient graphs	
**Tiikkaja, Sanna; Sandin, Sven; Hultman, Christina M.; Modin, Bitte; Malki, Ninoa; Sparen, Par**	Psychiatric disorder and work life: A longitudinal study of intra-generational social mobility	2016	[237]	graph is not associated to an individual patient	term "graph" is used for something different than for patient graphs	
**Toner, James P.; Coddington, Charles C.; Doody, Kevin; van Voorhis, Brad; Seifer,**	Society for Assisted Reproductive Technology and assisted reproductive	2016	[238]	graph is not associated to an individual patient	term "graph" is used for something different than for patient graphs	

**David B.; Ball, G. David; Luke, Barbara; Wantman, Ethan**	technology in the United States: a 2016 update					
**Torkar, Simon; Benedik, Peter; Rajkovic, Uros; Sustersic, Olga; Rajkovic, Vladislav**	Design of a Recommendation System for Adding Support in the Treatment of Chronic Patients	2016	[239]	term “graph” is used for something different than for patient graphs	term “graph” is used for something different than for patient graphs	
**Troszynski, Michal; Niemiec, Tomasz; Wilczynska, Anna**	Assessment of three-level selective perinatal care based on the analysis of early perinatal death rates and cesarean sections in Poland in 2008	2009	[240]	term “graph” is used for something different than for patient graphs	term “graph” is used for something different than for patient graphs	
**Truyen Tran; Dinh Phung; Luo, Wei; Venkatesh, Svetha**	Stabilized sparse ordinal regression for medical risk stratification	2015	[241]	term “graph” is used for something different than for patient graphs	term “graph” is used for something different than for patient graphs	
**Tukiendorf, A.**	Cervix uteri cancer incidence in relation to ethnic situation in Opole province, Poland	2002	[242]	term “graph” is used for something different than for patient graphs	term “graph” is used for something different than for patient graphs	
**Ugon, Adrien; Philippe, Carole; Pietrasz, Slawomir; Ganascia, Jean-Gabriel; Levy, Pierre P.**	OPTISAS a new method to analyse patients with Sleep Apnea Syndrome	2008	[243]	term “graph” is used for something different than for patient graphs	term “graph” is used for something different than for patient graphs	
**Valko, Michal; Kveton, Branislav; Valizadegan, Hamed; Cooper, Gregory F.; Hauskrecht, Milos**	Conditional Anomaly Detection with Soft Harmonic Functions	2011	[244]	term “graph” is used for something different than for patient graphs	term “graph” is used for something different than for patient graphs	
**Vallejo, I. L.; Herrero, H. H.; Sanz, J. J.C.; Martin, J. J.D.; Azarola, E. N.**	Diagnostic variability in a cohort of patients with multiple admissions in the last two decades	2003	[245]	term “graph” is used for something different than for patient graphs	term “graph” is used for something different than for patient graphs	
**Vani, S. N.**	Appropriate technologies for mother and child health care in developing countries	1989	[246]	graph is not associated to an individual patient	term “graph” is used for something different than for patient graphs	
**Vilares, M.; Ribadas, F. J.; Graña, J.**	Approximately Common Patterns in Shared-forests	2001	[247]	term “graph” is used for something different than for patient graphs	term “graph” is used for something different than for patient graphs	
**Voskoboinik, A.; Gutman, M. J.; Croagh, D.; Bell, R.; Saunder, A.; Gribbin, J.; Kanellis, J.**	Implementation and learning of laproscopic donor nephrectomy by a non-transplant general surgeon with advanced laparoscopic skills	2011	[248]	term “graph” is used for something different than for patient graphs	term “graph” is used for something different than for patient graphs	
**Vossler, D. G.; Am Haltiner; Schepp, S. K.; Friel, P. A.; Caylor, L. M.; Morgan, J. D.; Doherty, M. J.**	Ictal stuttering - A sign suggestive of psychogenic nonepileptic seizures	2004	[249]	term “graph” is used for something different than for patient graphs	term “graph” is used for something different than for patient graphs	
**Vossler, D. G.; Haltiner, A. M.; Schepp, S. K.; Friel, P. A.; Caylor, L. M.; Morgan, J. D.; Doherty, M. J.**	Ictal stuttering: a sign suggestive of psychogenic nonepileptic seizures	2004	[250]	Dublette	Dublette	
**Vranken, R.; Coulombier, D.; Kenyon, T.; Koosimile, B.; Mavunga, T.; Coggin, W.; Binkin, N.**	Use of a computerized tuberculosis register for automated generation of case finding,	2002	[251]	term “graph” is used for something different than for patient graphs	term “graph” is used for something different than for patient graphs	

	sputum conversion, and treatment outcome reports					
**Walley, J. D.; McDonald, M.**	Integration of mother and child health services in Ethiopia	1991	[252]	term “graph” is used for something different than for patient graphs	term “graph” is used for something different than for patient graphs	
**Wang, Zheng; Chakraborty, Prithwish; Mekaru, Sumiko R.; Brownstein, John S.; Ye, Jieping; Ramakrishnan, Naren**	Dynamic Poisson Autoregression for Influenza-Like-Illness Case Count Prediction	2015	[253]	term “graph” is used for something different than for patient graphs	term “graph” is used for something different than for patient graphs	
**Wang, Jason Tsong-Li; Chirn, Gung-Wei; Marr, Thomas G.; Shapiro, Bruce; Shasha, Dennis; Zhang, Kaizhong**	Combinatorial Pattern Discovery for Scientific Data: Some Preliminary Results	1994	[254]	term “graph” is used for something different than for patient graphs	term “graph” is used for something different than for patient graphs	
**Wang, Jason Tsong-Li; Chirn, Gung-Wei; Marr, Thomas G.; Shapiro, Bruce; Shasha, Dennis; Zhang, Kaizhong**	Combinatorial Pattern Discovery for Scientific Data: Some Preliminary Results	1994	[255]	Dublette	From multimodality digital imaging to multimedia patient record/ dupl	
**Wang, Biing-Feng; Lin, Chien-Hsin; Yang, I-Tse**	Constructing a Gene Team Tree in Almost O (N Lg N) Time	2014	[256]	term “graph” is used for something different than for patient graphs	term “graph” is used for something different than for patient graphs	
**Wang, X.; Wang, F.; Wang, J.; Qian, B.; Hu, J.**	Exploring Patient Risk Groups with Incomplete Knowledge	2013	[257]	graph is not associated to an individual patient	graph is not associated to an individual patient	
**Welch, Janet L.; Siek, Katie A.; Connelly, Kay H.; Astroth, Kim S.; McManus, M. Sue; Scott, Linda; Heo, Seongkum; Kraus, Michael A.**	Merging health literacy with computer technology: self-managing diet and fluid intake among adult hemodialysis patients	2010	[258]	term “graph” is used for something different than for patient graphs	term “graph” is used for something different than for patient graphs	
**White, D.; Choi, H.; Peloquin, C.; Zhu, Y.; Zhang, Y.**	Secular trend of adhesive capsulitis	2011	[259]	term “graph” is used for something different than for patient graphs	term “graph” is used for something different than for patient graphs	
**Wilhelms, Jane; van Gelder, Allen**	Octrees for Faster Isosurface Generation	1992	[260]	term “graph” is used for something different than for patient graphs	term “graph” is used for something different than for patient graphs	
**Winter, Alfred; Brigl, Birgit; Funkat, Gert; Haeber, Anke; Heller, Oliver; Wendt, Thomas**	3LGM(2)-Modeling to support management of health information systems	2007	[261]	term “graph” is used for something different than for patient graphs	term “graph” is used for something different than for patient graphs	
**Wongsuphasawat, Krist; Gotz, David**	Exploring Flow, Factors, and Outcomes of Temporal Event Sequences with the Outflow Visualization	2012	[262]	term “graph” is used for something different than for patient graphs	term “graph” is used for something different than for patient graphs	
**Wu, Taoyang; Moulton, Vincent; Steel, Mike**	Refining Phylogenetic Trees Given Additional Data: An Algorithm Based on Parsimony	2009	[263]	term “graph” is used for something different than for patient graphs	term “graph” is used for something different than for patient graphs	
**WU, Yubao; Zhu, Xiaofeng; Li, Li; Fan, Wei; Jin, Ruoming; Zhang, Xiang**	Mining Dual Networks: Models, Algorithms, and Applications	2016	[264]	term “graph” is used for something different than for patient graphs	term “graph” is used for something different than for patient graphs	

**Xie, W.; Wu, J.**	Mining positive and negative weighted association rules in medical records without user-specified weights based on HITS model	2010	[265]	term “graph” is used for something different than for patient graphs	term “graph” is used for something different than for patient graphs	
**Xie, C.; Yang, P.; Yang, Y.**	Open Knowledge Accessing Method in IoT-based Hospital Information System for Medical Record Enrichment	2018	[266]	term “graph” is used for something different than for patient graphs	term “graph” is used for something different than for patient graphs	
**Yamin, A.; Khan, S. A.; Yasin, U. U.**	Automated system of hess screen for diagnosis of paralytic strabismus using computer aided diagnosis	2013	[267]	term “graph” is used for something different than for patient graphs	term “graph” is used for something different than for patient graphs	
**Yoon, Sunmoo; Cohen, Bevin; Cato, Kenrick D.; Liu, Jianfang; Larson, Elaine L.**	Visualization of Data Regarding Infections Using Eye Tracking Techniques	2016	[268]	term “graph” is used for something different than for patient graphs	term “graph” is used for something different than for patient graphs	
**Yoon, Dukyong; Park, Inwhee; Schuemie, Martijn J.; Park, Man Young; Kim, Ju Han; Park, Rae Woong**	A quantitative method for assessment of prescribing patterns using electronic health records	2013	[269]	term “graph” is used for something different than for patient graphs	term “graph” is used for something different than for patient graphs	
**Zamora, M.; Baradad, M.; Amado, E.; Cordomí, S.; Limón, E.; Ribera, J.; Arias, M.; Gavaldà, R.**	Characterizing chronic disease and polymedication prescription patterns from electronic health records	2015	[270]	term “graph” is used for something different than for patient graphs	term “graph” is used for something different than for patient graphs	
**Zenios, S.**	Information Technology in Health Care Systems: Barriers to Adoption	2006	[271]	term “graph” is used for something different than for patient graphs	term “graph” is used for something different than for patient graphs	
**Zhang, He; Mehotra, Sanjay; Liebovitz, David; Gunter, Carl A.; Malin, Bradley**	Mining Deviations from Patient Care Pathways via Electronic Medical Record System Audits	2013	[272]	term “graph” is used for something different than for patient graphs	term “graph” is used for something different than for patient graphs	
**Zhang, Ming; Zhang, Hong; Tjandra, D.; Wong, S. T. C.**	DBMap: a space-conscious data visualization and knowledge discovery framework for biomedical data warehouse	2004	[273]	term “graph” is used for something different than for patient graphs	term “graph” is used for something different than for patient graphs	
**Zhao, Sendong**	Mining Medical Causality for Diagnosis Assistance	2017	[274]	term “graph” is used for something different than for patient graphs	term “graph” is used for something different than for patient graphs	
**Zheng, Ziwei; Wan, Xiaojun**	Graph-Based Multi-Modality Learning for Clinical Decision Support	2016	[275]	term “graph” is used for something different than for patient graphs	term “graph” is used for something different than for patient graphs	
**Zirkind, Givon**	Genetic Database Optimization: How Data Inspection and Consideration, Provides for Index Compression and Record Access Optimization of Genetic Databases	2006	[276]	term “graph” is used for something different than for patient graphs	term “graph” is used for something different than for patient graphs	
**ZWEIGENBAUM, P.**	MENELAS - AN ACCESS SYSTEM FOR MEDICAL RECORDS USING NATURAL-LANGUAGE	1994	[277]	term “graph” is used for something different than for patient graphs	term “graph” is used for something different than for patient graphs	

**Albarakati, N.; Obradovic, Z.**	Disease-Based Clustering of Hospital Admission: Disease Network of Hospital Networks Approach	2017	[278]	graph is not associated to an individual patient		term “graph” is used for something different than for patient graphs
**Andersen, A.**	An implementation of secure multi-party computations to preserve privacy when processing EMR data	2013	[279]	term “graph” is used for something different than for patient graphs		term “graph” is used for something different than for patient graphs
**Andersen, Anders**	SNOOP: Privacy Preserving Middleware for Secure Multi-party Computations	2014	[280]	term “graph” is used for something different than for patient graphs		term “graph” is used for something different than for patient graphs
**Andersen, A.; Yigzaw, K. Y.; Karlsen, R.**	Privacy preserving health data processing	2014	[281]	term “graph” is used for something different than for patient graphs		term “graph” is used for something different than for patient graphs
**Brunson, Jason Cory; Laubenbacher, Reinhard C.**	Applications of network analysis to routinely collected health care data: a systematic review	2018	[282]	term “graph” is used for something different than for patient graphs		term “graph” is used for something different than for patient graphs
**Chen, Jinpeng; Poon, Josiah; Poon, Simon K.; Xu, Ling; Sze, Daniel M. Y.**	Mining Symptom-Herb Patterns from Patient Records Using Tripartite Graph	2015	[283]	graph is not associated to an individual patient		graph is not associated to an individual patient
**Finney, John M.; Walker, A. Sarah; Peto, Tim E. A.; Wyllie, David H.**	An efficient record linkage scheme using graphical analysis for identifier error detection	2011	[284]	graph is not associated to an individual patient		graph is not associated to an individual patient
**Goodwin, Travis R.; Harabagiu, Sanda M.**	Medical Question Answering for Clinical Decision Support	2016	[285]	graph is not associated to an individual patient		graph is not associated to an individual patient
**Goodwin, Travis R.; Harabagiu, Sanda M.**	Knowledge Representations and Inference Techniques for Medical Question Answering	2018	[286]	graph is not associated to an individual patient		graph is not associated to an individual patient
**Hanzlicek, Petr; Spidlen, Josef; Heroutova, Helena; Nagy, Miroslav**	User interface of MUDR electronic health record	2005	[287]	term “graph” is used for something different than for patient graphs		term “graph” is used for something different than for patient graphs
**Herskovic, Jorge R.; Subramanian, Devika; Cohen, Trevor; Bozzo-Silva, Pamela A.; Bearden, Charles F.; Bernstam, Elmer V.**	Graph-based signal integration for high-throughput phenotyping	2012	[288]	term “graph” is used for something different than for patient graphs		term “graph” is used for something different than for patient graphs
**Honglan, L.; Xiaona, Q.; Bin, F.**	The Symptoms and Pathogenesis Entity Recognition of TCM Medical Records Based on CRF	2015	[289]	term “graph” is used for something different than for patient graphs		term “graph” is used for something

						different than for patient graphs
**Huet, B.; Artigou, J. Y.; Poirier, J.; Blain, G.**	Meta-modelling: the appropriate solution for a family of applications	2001	[290]	term "graph" is used for something different than for patient graphs		term "graph" is used for something different than for patient graphs
**Huet, B.; Pourriat, J. L.; Martin, J.; Cupa, M.**	An automaton computer program for a microcomputerized real-time (thesaurus based) abstract medical record	1982	[291]	term "graph" is used for something different than for patient graphs		term "graph" is used for something different than for patient graphs
**Jeunemaitre, X.; Degoulet, P.; Morice, V.; Chatellier, G.; Devries, C.; Plouin, P. F.; Boisvieux, J. F.; Menard, J.**	Testing an expert system for hypertension	1986	[292]	term "graph" is used for something different than for patient graphs		term "graph" is used for something different than for patient graphs
**Ji, Xiang; Ae Chun, Soon; Geller, James**	Predicting Comorbid Conditions and Trajectories using Social Health Records	2016	[293]	term "graph" is used for something different than for patient graphs		graph is not associated to an individual patient
**Kavuluru, Ramakanth; Han, Sifei; Harris, Daniel**	Unsupervised Extraction of Diagnosis Codes from EMRs Using Knowledge-Based and Extractive Text Summarization Techniques	2013	[294]	graph is not associated to an individual patient		term "graph" is used for something different than for patient graphs
**Kerschberger, Bernhard; Hilderbrand, Katherine; Boulle, Andrew M.; Coetzee, David; Goemaere, Eric; Azevedo, Virginia de; van Cutsem, Gilles**	The effect of complete integration of HIV and TB services on time to initiation of antiretroviral therapy: a before-after study	2012	[295]	graph is not associated to an individual patient		graph is not associated to an individual patient
**Koopman, Bevan**	Semantic Search As Inference: Applications in Health Informatics	2014	[296]	graph is not associated to an individual patient		general essay, no specific data example
**Koopman, Bevan; Zuccon, Guido; Bruza, Peter; Sitbon, Laurianne; Lawley, Michael**	Graph-based Concept Weighting for Medical Information Retrieval	2012	[297]	graph is not associated to an individual patient		graph is not associated to an individual patient
**Mondal, S.; Mukherjee, N.**	Mobile-assisted remote healthcare delivery	2016	[298]	term "graph" is used for something different than for patient graphs		graph is not associated to an individual patient
**Monsen, Karen A.; Banerjee, Arindam; Das, Puja**	Discovering client and intervention patterns in home visiting data	2010	[299]	term "graph" is used for something different than for patient graphs		graph is not associated to an individual patient
**Muller, R.; Sergl, M.; Nauerth, U.; Schoppe, D.; Pommerening, K.; Dittrich, H. M.**	THEMPO: a knowledge-based system for therapy planning in pediatric oncology	1997	[300]	term "graph" is used for something different than for patient graphs		graph is not associated to an individual patient
**Ni, J.; Fei, H.; Fan, W.; Zhang, X.**	Automated Medical Diagnosis by Ranking Clusters Across the Symptom-Disease Network	2017	[301]	term "graph" is used for something different than for patient graphs		graph is not associated to an individual patient

**O'Neil, M.; Payne, C.; Read, J.**	Read Codes Version 3: a user led terminology	1995	[302]	term “graph” is used for something different than for patient graphs		graph is not associated to an individual patient
**Pietrzyk, P. M.**	Free text analysis	1995	[303]	term “graph” is used for something different than for patient graphs		general essay, no specific data example
**Săcărea, C.; Şotropa, D.; Troancă, D.**	Symptoms investigation by means of formal concept analysis for enhancing medical diagnoses	2017	[304]	graph is not associated to an individual patient		graph is not associated to an individual patient
**Soulakis, Nicholas D.; Carson, Matthew B.; Lee, Young Ji; Schneider, Daniel H.; Skeehan, Connor T.; Scholtens, Denise M.**	Visualizing collaborative electronic health record usage for hospitalized patients with heart failure	2015	[305]	term “graph” is used for something different than for patient graphs		graph is not associated to an individual patient
**Zhang, Zhaoyang; Wang, Honggang; Wang, Chonggang; Fang, Hua**	Cluster-based Epidemic Control Through Smartphone-based Body Area Networks	2015	[306]	graph is not associated to an individual patient		graph is not associated to an individual patient
**Adamusiak, Tomasz; Shimoyama, Naoki; Shimoyama, Mary**	Next generation phenotyping using the unified medical language system	2014	[307]		No graph mentioned	graph is not associated to an individual patient
**Alvarez, Jose Mar\'ıa; Polo, Luis; Jimenez, Weena; Abella, Pablo; Labra, Jose Emilio**	Application of the Spreading Activation Technique for Recommending Concepts of Well-known Ontologies in Medical Systems	2011	[308]		Not associated with patient	graph is not associated to patient data but to spoken language about a patient
**Antonakakis, Marios; Dimitriadis, Stavros I.; Zervakis, Michalis; Micheloyannis, Sifis; Rezaie, Roozbeh; Babajani-Feremi, Abbas; Zouridakis, George; Papanicolaou, Andrew C.**	Altered cross-frequency coupling in resting-state MEG after mild traumatic brain injury	2016	[309]		Not used for patient data in general but brain regions	graph is not associated to patient data but to spoken language about a patient
**Arantes, W. Manzi de; Verdier, C.**	Public health alert system for health networks: application to cardiology	2005	[310]		Not used for patient data directly	graph is not associated to patient data but to spoken language about a patient
**Athreya, A. P.; Ngiam, K. Y.; Luo, Z.; Tai, E. S.; Kalbarczyk, Z.; Iyer, R. K.**	Towards Longitudinal Analysis of a Population’s Electronic Health Records Using Factor Graphs	2016	[311]		Pupulation data, not individuals	graph is not associated to an individual patient
**Dhulekar, Nimit; Oztan, Basak; Yener, Bülent; Bingol, Haluk O.; Irim, Gulcin; Aktekin, Berrin; Aykut-Bingöl, Canan**	Graph-theoretic Analysis of Epileptic Seizures on Scalp EEG Recordings	2014	[312]		no patient data in general, only eeg	graph is not associated to patient data but to spoken language about a patient
**Frazier, George F.**	An Incremental Algorithm for Building Temporal Quadtrees	1993	[313]		no patient data	term “graph” is used for something

						different than for patient graphs
**Gerson, C. D.; Gerson, M-J**	Technical report: an ePRO patient reported outcome program for the evaluation of patients with irritable bowel syndrome	2014	[314]		graph as visualization	term “graph” is used for something different than for patient graphs
**Ghosh, Priyanka; Kalyanaraman, Ananth**	A Fast Sketch-based Assembler for Genomes	2016	[315]		no patient data but gene assembly	term “graph” is used for something different than for patient graphs
**Gopakumar, Shivapratap; Tran, Truyen; Nguyen, Tu Dinh; Phung, Dinh; Venkatesh, Svetha**	Stabilizing high-dimensional prediction models using feature graphs	2015	[316]		no data of individual patients	graph is not associated to an individual patient
**Heer, Jeffrey; Perer, Adam**	Orion: A system for modeling, transformation and visualization of multidimensional heterogeneous networks	2014	[317]		no data of individual patients	graph is not associated to an individual patient
**Jing, X.; Cimino, J. J.**	A complementary graphical method for reducing and analyzing large data sets. Case studies demonstrating thresholds setting and selection	2014	[318]		no graph representation of data but graph reduction	Only a converter for semantic data representation database
**Jupin, Joseph; Shi, Justin Y.**	Identity Tracking in Big Data: Preliminary Research Using In-Memory Data Graph Models for Record Linkage and Probabilistic Signature Hashing for Approximate String Matching in Big Health and Human Services Databases	2014	[319]		no graph representation of data but record linkage	general essay, no specific data example
**Lieberman, Michael D.; Taheri, Sima; Guo, whatever; Mirrashed, Fatemeh; Yahav, Inbal; Aris, Aleks; Shneiderman, Ben**	Visual Exploration Across Biomedical Databases	2011	[320]		graph as visualization	term “graph” is used for something different than for patient graphs
**Luz, Saturnino; Kane, Bridget**	Classification of Patient Case Discussions Through Analysis of Vocalisation Graphs	2009	[321]		term “graph” is used for something different than for patient graphs	graph is not associated to patient data but to spoken language about a patient
**Mattson, Donald C.; Yang, Jing**	The Child Therapy Tracking System (CTTS): A model for an expressive therapy electronic health record (EHR)	2013	[322]		term “graph” is used for something different than for patient graphs	term “graph” is used for something different than for patient graphs
**Muller, R.**	The CliniCon framework for context representation in electronic patient records	1997	[323]		term “graph” is used for something different than for patient graphs	graph is not associated to an individual patient

**Noren, G. Niklas; Hopstadius, Johan; Bate, Andrew; Star, Kristina; Edwards, I. Ralph**	Temporal pattern discovery in longitudinal electronic patient records	2010	[324]		graph is not associated to an individual patient	term “graph” is used for something different than for patient graphs
**Nose, Y.; Akazawa, K.; Watanabe, Y.; Yokota, M.; Okamura, S.; Maehara, Y.; Sugimachi, K.**	Cancer registration using case history database in hospital information system	1988	[325]		term “graph” is used for something different than for patient graphs	term “graph” is used for something different than for patient graphs
**Ogushi, Y.; Haruki, Y.; Okada, Y.; Takahashi, M.; Shimizu, M.; Izumi, Y.; Watabe, T.; Kobayashi, S.; Okuyama, J.; Kurita, Y.**	Development and evaluation of regional health database systems	1998	[326]		term “graph” is used for something different than for patient graphs	term “graph” is used for something different than for patient graphs
**Oladimeji, Ebenezer A.; Chung, Lawrence; Jung, Hyo Taeg; Kim, Jaehyoun**	Managing Security and Privacy in Ubiquitous eHealth Information Interchange	2011	[327]		term “graph” is used for something different than for patient graphs	term “graph” is used for something different than for patient graphs
**Onimura, N.; Yamashita, T.; Nakayama, N.; Soejima, H.; Hirokawa, S.**	Generation of Sentence Template Graph from SOAP Format Medical Documents	2016	[328]		graph is not associated to an individual patient	term “graph” is used for something different than for patient graphs
**Oshiro, Tatsuo; Oshiro, Hisako; Tanimizu, Masahito**	Effective methods of organizing complex information in advanced cancer patients	2014	[329]		graph is not associated to an individual patient	term “graph” is used for something different than for patient graphs
**Osop, H.; Sahama, T.**	Electronic health records: Improvement to healthcare decision-making	2016	[330]		graph is not associated to an individual patient	term “graph” is used for something different than for patient graphs
**Poscia, Roberto; Ghio, Stefano; D'Alto, Michele; Vitulo, Patrizio; Mule, Massimilano; Albera, Carlo; Parisi, Francesco; Badagliacca, Roberto; Fedele, Francesco; Vizza, Carmine Dario**	'Real-life' information on pulmonary arterial hypertension: the iPHnet Project	2014	[331]		term “graph” is used for something different than for patient graphs	term “graph” is used for something different than for patient graphs
**Staubert, S.; Schaaf, M.; Jahn, F.; Brandner, R.; Winter, A.**	Modeling Interoperable Information Systems with 3LGM(2) and IHE	2015	[332]		term “graph” is used for something different than for patient graphs	term “graph” is used for something different than for patient graphs
**Techentin, R.; Sauver, J. S.; Huddleston, J.; Gilbert, B.; Holmes, D.**	Lessons learned from the semantic translation of healthcare data	2014	[333]		graph is not associated to an individual patient	Only a converter for semantic data representation database

**Whyatt, Caroline P.; Torres, Elizabeth B.**	The Social-dance: Decomposing Naturalistic Dyadic Interaction Dynamics to the 'Micro-level'	2017	[334]		graph is not associated to an individual patient	graph is not associated to an individual patient

References

1. Abbs v. Sullivan. Fed Report. 1992;963:918–29.
2. Adin CA, Gregory CR, Kyles AE, Cowgill L. Diagnostic predictors of complications and survival after renal transplantation in cats. VETERINARY SURGERY. 2001;30:515–21. doi:10.1053/jvet.2001.28418.
3. Agogo GO, van der Voet H, van't Veer P, Ferrari P, Leenders M, Muller DC, et al. Use of two-part regression calibration model to correct for measurement error in episodically consumed foods in a single-replicate study design: EPIC case study. PLoS One. 2014;9:e113160. doi:10.1371/journal.pone.0113160.
4. Amit M, Yen TC, Liao CT, Chaturvedi P, Agarwal JP, Kowalski LP, et al. The origin of regional failure in oral cavity squamous cell carcinoma with pathologically negative neck metastases. JAMA Otolaryngol Head Neck Surg. 2014;140:1130–7. doi:10.1001/jamaoto.2014.1539.
5. Andreano A, Rebora P, Valsecchi MG, Russo AG. Adherence to guidelines and breast cancer patients survival: a population-based cohort study analyzed with a causal inference approach. Breast Cancer Res Treat. 2017;164:119–31. doi:10.1007/s10549-017-4210-z.
6. Anindya IC, Roy H, Kantarcioglu M, Malin B. Building a Dossier on the Cheap: Integrating Distributed Personal Data Resources Under Cost Constraints. In: New York, NY, USA: ACM; 2017. p. 1549–1558. doi:10.1145/3132847.3132951.
7. Antani S. Integrating image and text information for biomedical information retrieval. In: ; 2010. p. 3. doi:10.1109/CBMS.2010.6042631.
8. Anwar MN, Oakes MP. Data Mining of Audiology Patient Records: Factors Influencing the Choice of Hearing Aid Type. In: New York, NY, USA: ACM; 2011. p. 11–18. doi:10.1145/2064696.2064701.
9. Anwar MN, Oakes MP. Data mining of audiology patient records: factors influencing the choice of hearing aid type. BMC Med Inform Decis Mak. 2012;12 Suppl 1:S6. doi:10.1186/1472-6947-12-S1-S6.
10. Arcilla MJB, Ang VCT, Ochoa MN, Padua CPL, Payawal RE. Kidnew: The Kidney Transplant Patient Personal Health Buddy. In: New York, NY, USA: ACM; 2015. p. 133–134. doi:10.1145/2750511.2750539.
11. Atreja A, Khan S, Rogers JD, Otobo E, Patel NP, Ullman T, et al. Impact of the Mobile HealthPROMISE Platform on the Quality of Care and Quality of Life in Patients With Inflammatory Bowel Disease: Study Protocol of a Pragmatic Randomized Controlled Trial. JMIR Res Protoc. 2015;4:e23. doi:10.2196/resprot.4042.
12. Bakel LA, Wilson K, Tyler A, Tham E, Reese J, Bothner J, Kaplan DW. A quality improvement study to improve inpatient problem list use. Hosp Pediatr. 2014;4:205–10. doi:10.1542/hpeds.2013-0060.
13. Baptista Macaroff WM, Castroman Espasandin P. Analgesic quality in a postoperative pain service: continuous assessment with the cumulative sum (cusum) method. Rev Esp Anestesiol Reanim. 2007;54:11–6.
14. Barsky M, Stege U, Thomo A, Upton C. A Graph Approach to the Threshold All-against-all Substring Matching Problem. J. Exp. Algorithmics. 2008;12:1.10:1. doi:10.1145/1227161.1370601.
15. Bassily MN, Wilson R, Pompei F, Burmistrov D. Cancer survival as a function of age at diagnosis: a study of the Surveillance, Epidemiology and End Results database. Cancer Epidemiol. 2010;34:667–81. doi:10.1016/j.canep.2010.04.013.
16. Baud RH, Am RASSINOUX, SCHERRER, JR. NATURAL-LANGUAGE PROCESSING AND SEMANTICAL REPRESENTATION OF MEDICAL TEXTS. Methods Inf Med. 1992;31:117–25.
17. Baud RH, Am RASSINOUX, WAGNER JC, Lovis C, JUGE C, ALPAY LL, et al. REPRESENTING CLINICAL NARRATIVES USING CONCEPTUAL GRAPHS. Methods Inf Med. 1995;34:176–86.
18. Baud RH, Rassinoux AM, Scherrer JR. Natural language processing and semantical representation of medical texts. Methods Inf Med. 1992;31:117–25.
19. Bean DM, Wu H, Iqbal E, Dzahini O, Ibrahim ZM, Broadbent M, et al. Knowledge graph prediction of unknown adverse drug reactions and validation in electronic health records. Sci Rep. 2017;7:16416. doi:10.1038/s41598-017-16674-x.
20. Bean DM, Wu H, Iqbal E, Dzahini O, Ibrahim ZM, Broadbent M, et al. Author Correction: Knowledge graph prediction of unknown adverse drug reactions and validation in electronic health records. Sci Rep. 2018;8:4284. doi:10.1038/s41598-018-22521-4.
21. Beilin LJ, Bulpitt CJ, Coles EC, Dollery CT, Johnson BF, Mearns C, et al. Computer-based hypertension clinic records: a co-operative study. Br Med J. 1974;2:212–6.
22. Bellin E, Kalkut G. Is time-slice analysis superior to total hospital length of stay in demonstrating the effectiveness of a month-long intensive effort on a medicine service? Qual Manag Health Care. 2004;13:143–9.

23. Benedik P, Rajkovic U, Sustersic O. Toward the design of a nursing ontology system. Comput Inform Nurs. 2014;32:580–8. doi:10.1097/CIN.0000000000000117.
24. Bereg S, Zhang Y. Phylogenetic Networks Based on the Molecular Clock Hypothesis. IEEE/ACM Trans. Comput. Biol. Bioinformatics. 2007;4:661–7. doi:10.1109/tcbb.2007.1043.
25. Bernauer J, Franz M, Schoop D, Schoop M, Pretschner DP. The compositional approach for representing medical concept systems. Medinfo. 1995;8 Pt 1:70–4.
26. Bertrand D, Gascuel O. Topological Rearrangements and Local Search Method for Tandem Duplication Trees. IEEE/ACM Trans. Comput. Biol. Bioinformatics. 2005;2:15–28. doi:10.1109/TCBB.2005.15.
27. Bingham A. Computerized patient records benefit physician offices. Healthc Financ Manage. 1997;51:68–70.
28. Bono B de, Helvensteijn M, Kokash N, Martorelli I, Sarwar D, Islam S, et al. Requirements for the formal representation of pathophysiology mechanisms by clinicians. INTERFACE FOCUS 2016. doi:10.1098/rsfs.2015.0099.
29. Bordewich M, Gascuel O, Huber KT, Moulton V. Consistency of Topological Moves Based on the Balanced Minimum Evolution Principle of Phylogenetic Inference. IEEE/ACM Trans. Comput. Biol. Bioinformatics. 2009;6:110–7. doi:10.1109/TCBB.2008.37.
30. Brewer NT, Gilkey MB, Lillie SE, Hesse BW, Sheridan SL. Tables or bar graphs? Presenting test results in electronic medical records. Med Decis Making. 2012;32:545–53. doi:10.1177/0272989X12441395.
31. Britto MT, Jimison HB, Munafo JK, Wissman J, Rogers ML, Hersh W. Usability testing finds problems for novice users of pediatric portals. J Am Med Inform Assoc. 2009;16:660–9. doi:10.1197/jamia.M3154.
32. Brown AL. Constructing Chromosome Scale Suffix Trees. In: Darlinghurst, Australia, Australia: Australian Computer Society, Inc; 2004. p. 105–112.
33. Buchtela D, Anger Z, Peleska J, Vesely A, Zvarova J. Presentation of medical guidelines on a computer. Stud Health Technol Inform. 2004;105:166–71.
34. Bui AAT, Taira RK, Churchill B, Kangarloo H. Integrated visualization of problemcentric urologic patient records. Ann N Y Acad Sci. 2002;980:267–77.
35. Burgess HA. Use of the labor graph in Malawi. J Nurse Midwifery. 1986;31:46–52.
36. Butch SH. Practical use of computerized hospital information systems to improve blood transfusion. Am J Clin Pathol. 1997;107:S50-6.
37. Cates W, JR. Legal abortion: the public health record. Science. 1982;215:1586–90.
38. Cen S, Han L, Ma J. Ranking Weblogs by Analyzing Reading and Commenting Activities. In: Washington, DC, USA: IEEE Computer Society; 2009. p. 442–449. doi:10.1109/WI-IAT.2009.77.
39. Chazelle B. Technical Perspective: Finding a Good Neighbor, Near and Fast. Commun. ACM. 2008;51:115. doi:10.1145/1327452.1327493.
40. Chen P, Zhao T, Feng R, Chai J, Tong G, Wang D. Patterns and trends with cancer incidence and mortality rates reported by the China National Cancer Registry. Asian Pac J Cancer Prev. 2014;15:6327–32.
41. Chennamsetty H, Chalasani S, Riley D. Predictive analytics on Electronic Health Records (EHRs) using Hadoop and Hive. In: ; 2015. p. 1–5. doi:10.1109/ICECCT.2015.7226129.
42. Christinat Y, Moret BME. A Transcript Perspective on Evolution. IEEE/ACM Trans. Comput. Biol. Bioinformatics. 2013;10:1403–11. doi:10.1109/TCBB.2012.145.
43. Christofidis MJ, Hill A, Horswill MS, Watson MO. Observation charts with overlapping blood pressure and heart rate graphs do not yield the performance advantage that health professionals assume: an experimental study. J Adv Nurs. 2014;70:610–24. doi:10.1111/jan.12223.
44. Chung HH, Kim JW, Kang KW, Park N, Song Y, Chung J, Kang S. Post-treatment (1)(8)FFDG maximum standardized uptake value as a prognostic marker of recurrence in endometrial carcinoma. Eur J Nucl Med Mol Imaging. 2011;38:74–80. doi:10.1007/s00259-010-1614-y.
45. Cimino JJ, Clayton PD, Hripcsak G, Johnson SB. Knowledge-based approaches to the maintenance of a large controlled medical terminology. J Am Med Inform Assoc. 1994;1:35–50.
46. Collier T, Steenkamp R, Tomson C, Caskey F, Ansell D, Roderick P, Nitsch D. Patterns and effects of missing comorbidity data for patients starting renal replacement therapy in England, Wales and Northern Ireland. Nephrol Dial Transplant. 2011;26:3651–8. doi:10.1093/ndt/gfr111.
47. Cota E, Ribeiro L, Bezerra JS, Costa A, da Silva RE, Cota G. Using formal methods for content validation of medical procedure documents. Int J Med Inform. 2017;104:10–25. doi:10.1016/j.ijmedinf.2017.04.012.
48. Cruz JP, Libatique NJ, Tangonan G. Steganography and data hiding in flash video (FLV). In: ; 2012. p. 1–6. doi:10.1109/TENCON.2012.6412279.
49. Cypko MA, Wojdziak J, Stoehr M, Kirchner B, Preim B, Dietz A, et al. Visual Verification of Cancer Staging for Therapy Decision Support. COMPUTER GRAPHICS FORUM. 2017;36:109–20. doi:10.1111/cgf.13172.
50. Dabek F, Caban JJ. A grammar-based approach to model the patient's clinical trajectory after a mild traumatic brain injury. In: ; 2015. p. 723–730. doi:10.1109/BIBM.2015.7359775.
51. Dabek F, Chen J, Garbarino A, Caban JJ. Visualization of Longitudinal Clinical Trajectories Using a Graph-based Approach. In: New York, NY, USA: ACM; 2015. p. 5:1. doi:10.1145/2836034.2836039.
52. Dahl MR, Vedsted P. Personal data and confidentiality on the Internet. Ugeskr Laeger. 2008;170:4027–9.

53. Danaei G, Garcia Rodriguez LA, Fernandez Cantero O, Logan R, Hernan MA. Observational data for comparative effectiveness research: An emulation of randomised trials of statins and primary prevention of coronary heart disease. STATISTICAL METHODS IN MEDICAL RESEARCH. 2013;22:70–96. doi:10.1177/0962280211403603.
54. Davidson S, Natan D, Novikov I, Sokolover N, Erlich A, Shamir R. Body mass index and weight-for-length ratio references for infants born at 33-42 weeks gestation: a new tool for anthropometric assessment. Clin Nutr. 2011;30:634–9. doi:10.1016/j.clnu.2011.03.010.
55. Degnan JH, Rosenberg NA, Stadler T. A Characterization of the Set of Species Trees That Produce Anomalous Ranked Gene Trees. IEEE/ACM Trans. Comput. Biol. Bioinformatics. 2012;9:1558–68. doi:10.1109/TCBB.2012.110.
56. Delman BS. A problem-oriented approach to journal selection for hospital libraries. Bull Med Libr Assoc. 1982;70:397–410.
57. Dent RM, Penwarden RM, Harris N, Hotz SB. Development and evaluation of patient-centered software for a weight-management clinic. OBESITY RESEARCH. 2002;10:651–6. doi:10.1038/oby.2002.88.
58. Dietz KR, Zhang L, Seidel FG. The transverse diameter of the chest on routine radiographs reliably estimates gestational age and weight in premature infants. Pediatr Radiol. 2015;45:1303–7. doi:10.1007/s00247-015-3332-x.
59. Diprose GK, Evans DH, Levene MI. A microcomputer monitoring and data-acquisition system for intensive care units. J Med Eng Technol. 1985;9:80–4.
60. Elliott D, Allen E, McKinley S, Perry L, Duffield C, Fry M, et al. User acceptance of observation and response charts with a track and trigger system: a multisite staff survey. J Clin Nurs. 2016;25:2211–22. doi:10.1111/jocn.13303.
61. Engholm G, Ferlay J, Christensen N, Bray F, Gjerstorff ML, Klint A, et al. NORDCAN--a Nordic tool for cancer information, planning, quality control and research. Acta Oncol. 2010;49:725–36. doi:10.3109/02841861003782017.
62. Erkal S, Gerberich SG, Ryan AD, Renier CM, Alexander BH. Animal-related injuries: a population-based study of a five-state region in the upper Midwest: Regional Rural Injury Study II. J Safety Res. 2008;39:351–63. doi:10.1016/j.jsr.2008.03.002.
63. Evandt J, Oftedal B, Krog NH, Skurtveit S, Nafstad P, Schwarze PE, et al. Road traffic noise and registry based use of sleep medication. Environ Health. 2017;16:110. doi:10.1186/s12940-017-0330-5.
64. Evans JM, MacDonald TM. Misclassification and selection bias in case-control studies using an automated database. Pharmacoepidemiol Drug Saf. 1997;6:313–8. doi:10.1002/(SICI)1099-1557(199709/10)6:5<313::AID-PDS292>3.0.CO;2-K.
65. Faeder JR, Blinov ML, Hlavacek WS. Graphical Rule-based Representation of Signal-transduction Networks. In: New York, NY, USA: ACM; 2005. p. 133–140. doi:10.1145/1066677.1066712.
66. Faloutsos C. Future Directions in Data Mining: Streams, Networks, Self-similarity and Power Laws. In: New York, NY, USA: ACM; 2002. p. 93. doi:10.1145/584792.584794.
67. Faloutsos C, Kamel I. Beyond Uniformity and Independence: Analysis of R-trees Using the Concept of Fractal Dimension. In: New York, NY, USA: ACM; 1994. p. 4–13. doi:10.1145/182591.182593.
68. Fan K, Eisenberg M, Walsh A, Aiello A, Heller K. Hierarchical Graph-Coupled HMMs for Heterogeneous Personalized Health Data. In: New York, NY, USA: ACM; 2015. p. 239–248. doi:10.1145/2783258.2783326.
69. Farfan F, Hristidis V, Ranganathan A, Burke RP. Ontology-Aware Search on XML-based Electronic Medical Records. In: ; 2008. p. 1525–1527. doi:10.1109/ICDE.2008.4497611.
70. Fazeli Dehkordy S, Carlos RC, Hall KS, Dalton VK. Novel data sources for women's health research: mapping breast screening online information seeking through Google trends. Acad Radiol. 2014;21:1172–6. doi:10.1016/j.acra.2014.05.005.
71. Feng J, Zhu D. Faster Algorithms for Sorting by Transpositions and Sorting by Block Interchanges. ACM Trans. Algorithms 2007. doi:10.1145/1273340.1273341.
72. Ferreira TS, Luiz de Souza-Braga A, Soraia Cavalcanti-Valente G, Ferreira de Souza D, Moreira Carvalho-Alves E. Nursing audit: The impact of nursing annotation in the context of hospital gloss. AQUICHAN. 2009;9:38–49.
73. Finlayson SG, LePendu P, Shah NH. Building the graph of medicine from millions of clinical narratives. Sci Data. 2014;1:140032. doi:10.1038/sdata.2014.32.
74. Flack JR. Seven years experience with a computerized diabetes clinic database. Medinfo. 1995;8 Pt 1:332.
75. Florit AM, Mori AR, Simone M de, d'Annunzio V, Ricci FL, Lalle C. Context trees: representing co-operative healthcare activities in IREP. Comput Methods Programs Biomed. 1995;48:175–81.
76. Folio LR, Machado LB, Dwyer AJ. Multimedia-enhanced Radiology Reports: Concept, Components, and Challenges. Radiographics. 2018;38:462–82. doi:10.1148/rg.2017170047.
77. Forister JFY, Sun A, Weissman BA. Progress report on a post-radial keratotomy patient 20 years after surgery. Eye Contact Lens. 2007;33:334–7. doi:10.1097/ICL.0b013e318030f1b6.
78. Fors UG, Sandberg HC. Computer-aided risk management--a software tool for the Hidep model. Quintessence Int. 2001;32:309–20.
79. Fraccaro P, Vigo M, Balatsoukas P, van der Veer, Sabine N., Hassan L, Williams R, et al. Presentation of laboratory test results in patient portals: influence of interface design on risk interpretation and visual search behaviour. BMC Med Inform Decis Mak 2018. doi:10.1186/s12911-018-0589-7.
80. Friesen MR, Hamel C, McLeod RD. A mHealth application for chronic wound care: findings of a user trial. Int J Environ Res Public Health. 2013;10:6199–214. doi:10.3390/ijerph10116199.

81. Gail MH, Costantino JP, Pee D, Bondy M, Newman L, Selvan M, et al. Projecting individualized absolute invasive breast cancer risk in African American women. J Natl Cancer Inst. 2007;99:1782–92. doi:10.1093/jnci/djm223.
82. Galopin A, Bouaud J, Pereira S, Seroussi B. Using an ontological modeling to evaluate the consistency of clinical practice guidelines: application to the comparison of three guidelines on the management of adult hypertension. Stud Health Technol Inform. 2014;205:38–42.
83. Galopin A, Bouaud J, Pereira S, Seroussi B. An Ontology-Based Clinical Decision Support System for the Management of Patients with Multiple Chronic Disorders. Stud Health Technol Inform. 2015;216:275–9.
84. Garla V, Taylor C, Brandt C. Semi-supervised clinical text classification with Laplacian SVMs: an application to cancer case management. J Biomed Inform. 2013;46:869–75. doi:10.1016/j.jbi.2013.06.014.
85. Garrett WF, Fuchs H, Whitton MC, State A. Real-time Incremental Visualization of Dynamic Ultrasound Volumes Using Parallel BSP Trees. In: Los Alamitos, CA, USA: IEEE Computer Society Press; 1996. p. 235-ff.
86. Gevins AS, Yeager CL, Diamond SL. Interactive Analysis and Display of the Electroencephalogram (EEG) in Real Time. In: New York, NY, USA: ACM; 1974. p. 75–76. doi:10.1145/563182.563233.
87. Gibson TA, Goldberg DS. Evaluating Theoretical Models of Protein Interaction Network Evolution Without Seed Graphs. In: New York, NY, USA: ACM; 2013. p. 724:724. doi:10.1145/2506583.2512388.
88. Glinkowski W. Web-Based Support for Fracture Healing Evaluation and Monitoring. TELEMEDICINE AND E-HEALTH. 2011;17:201–10. doi:10.1089/tmj.2010.0131.
89. Goodwin T, Harabagiu S. Embedding Open-domain Common-sense Knowledge from Text. LREC Int Conf Lang Resour Eval. 2016;2016:4621–8.
90. Grinspan ZM, Abramson EL, Banerjee S, Kern LM, Kaushal R, Shapiro JS. Potential value of health information exchange for people with epilepsy: crossover patterns and missing clinical data. AMIA Annu Symp Proc. 2013;2013:527–36.
91. Gunter C. Detecting Roles and Anomalies in Hospital Access Audit Logs. In: New York, NY, USA: ACM; 2014. p. 1. doi:10.1145/2665936.2668879.
92. Guzzi PH, Mina M. Towards the Assessment of Semantic Similarity Analysis of Protein Data: Main Approaches and Issues. ACM SIGBioinformatics Rec. 2012;2:17–8. doi:10.1145/2384691.2384694.
93. Hackney DN, Knudtson EJ, Rossi KQ, Krugh D, O'Shaughnessy RW. Management of pregnancies complicated by anti-c isoimmunization. Obstet Gynecol. 2004;103:24–30. doi:10.1097/01.AOG.0000109206.22354.2C.
94. Halachev M, Shiri N, Thamildurai A. Exact Match Search in Sequence Data Using Suffix Trees. In: New York, NY, USA: ACM; 2005. p. 123–130. doi:10.1145/1099554.1099579.
95. Harsola A, Thale S, Panse MS. Low Cost Digital Stethoscope for Heart Sounds. In: New York, NY, USA: ACM; 2011. p. 1275–1279. doi:10.1145/1980022.1980304.
96. Hartzler AL, Izard JP, Dalkin BL, Mikles SP, Gore JL. Design and feasibility of integrating personalized PRO dashboards into prostate cancer care. JOURNAL OF THE AMERICAN MEDICAL INFORMATICS ASSOCIATION. 2016;23:38–47. doi:10.1093/jamia/ocv101.
97. Hausler R, Washburn D, Rey P, Stefanoff F. Acquiring otoneurological results by computer. Evaluation after 2 years of experience. Ann Otolaryngol Chir Cervicofac. 1982;99:403–8.
98. Henry SB, Mead CN. Nursing classification systems: necessary but not sufficient for representing "what nurses do" for inclusion in computer-based patient record systems. J Am Med Inform Assoc. 1997;4:222–32.
99. Henzinger MR, King V, Warnow T. Constructing a Tree from Homeomorphic Subtrees, with Applications to Computational Evolutionary Biology. In: Philadelphia, PA, USA: Society for Industrial and Applied Mathematics; 1996. p. 333–340.
100. Herman T, Pemmaraju SV, Segre AM, Polgreen PM, Curtis DE, Fries J, et al. Wireless Applications for Hospital Epidemiology. In: New York, NY, USA: ACM; 2009. p. 45–50. doi:10.1145/1540373.1540384.
101. Hettne K, Soiland-Reyes S, Klyne G, Belhajjame K, Gamble M, Bechhofer S, et al. Workflow Forever: Semantic Web Semantic Models and Tools for Preserving and Digitally Publishing Computational Experiments. In: New York, NY, USA: ACM; 2012. p. 36–37. doi:10.1145/2166896.2166909.
102. Hofer C, Kiechl S, Lang W. The Austrian Stroke-Unit-Registry. Wien Med Wochenschr. 2008;158:411–7. doi:10.1007/s10354-008-0563-6.
103. Hoimyr H, Christensen TD, Emmertsen K, Johnsen SP, Riis A, Hansen OK, Hjortdal VE. Surgical repair of coarctation of the aorta: up to 40 years of follow-up. Eur J Cardiothorac Surg. 2006;30:910–6. doi:10.1016/j.ejcts.2006.09.016.
104. Hripcsak G, Soulakis ND, Li L, Morrison FP, Lai AM, Friedman C, et al. Syndromic surveillance using ambulatory electronic health records. J Am Med Inform Assoc. 2009;16:354–61. doi:10.1197/jamia.M2922.
105. Huang H, Gong T, Ye N, Wang R, Dou Y. Private and Secured Medical Data Transmission and Analysis for Wireless Sensing Healthcare System. IEEE Transactions on Industrial Informatics. 2017;13:1227–37. doi:10.1109/TII.2017.2687618.

106. Huesch MD, Schetter S, Segel J, Chetlen A. Evaluation of the “Angelina Jolie Effect” on Screening Mammography Utilization in an Academic Center. JOURNAL OF THE AMERICAN COLLEGE OF RADIOLOGY. 2017;14:1020–6. doi:10.1016/j.jacr.2017.03.016.
107. Islam SKH, Khan MK, Li X. Security Analysis and Improvement of 'a More Secure Anonymous User Authentication Scheme for the Integrated EPR Information System'. PLoS One 2015. doi:10.1371/journal.pone.0131368.
108. Italian cancer figures, report 2012: Cancer in children and adolescents. Epidemiol Prev. 2013;37:1–225.
109. Jacky JP, Kalet IJ. An Object-oriented Programming Discipline for Standard Pascal. Commun. ACM. 1987;30:772–6. doi:10.1145/30401.30403.
110. Jamakovic M, Baljic R. Significance of copper level in serum and routine laboratory parameters in estimation of outspreading of Hodgkin's lymphoma. Med Arch. 2013;67:185–7.
111. Jamil HM, Modica GA, Teran MA. Towards a Visual Query Interface for Phylogenetic Databases. In: New York, NY, USA: ACM; 2001. p. 57–64. doi:10.1145/502585.502596.
112. Jamison RN, Jurcik DC, Edwards RR, Huang C, Ross EL. A Pilot Comparison of a Smartphone App With or Without 2-Way Messaging Among Chronic Pain Patients: Who Benefits From a Pain App? Clin J Pain. 2017;33:676–86. doi:10.1097/AJP.0000000000000455.
113. Kawada T, Aoki S, Suzuki S. Management of personal health examination data for a population by use of a portable computer. Nihon Koshu Eisei Zasshi. 1992;39:105–7.
114. Kay M, Choe EK, Shepherd J, Greenstein B, Watson N, Consolvo S, Kientz JA. Lullaby: A Capture & Access System for Understanding the Sleep Environment. In: New York, NY, USA: ACM; 2012. p. 226–234. doi:10.1145/2370216.2370253.
115. Kazmierczak SC, Leen TK, Erdogmus D, Carreira-Perpinan MA. Reduction of multi-dimensional laboratory data to a two-dimensional plot: a novel technique for the identification of laboratory error. Clin Chem Lab Med. 2007;45:749–52. doi:10.1515/CCLM.2007.177.
116. Keenan GM, Lopez KD, Yao Y, Sousa VEC, Stifter J, Febretti A, et al. Toward Meaningful Care Plan Clinical Decision Support: Feasibility and Effects of a Simulated Pilot Study. Nurs Res. 2017;66:388–98. doi:10.1097/NNR.0000000000000234.
117. Kennedy I, Ritter H. Antenatal records: do they help us? A new record for watching fetal growth. Trop Doct. 1984;14:130–2. doi:10.1177/004947558401400310.
118. Kennedy I, Stephens B. A novel antenatal record to help midwives. Practitioner. 1979;223:18–24.
119. Khan IA. Personalized Electronic Health Record System for Monitoring Patients with Chronic Disease. In: ; 2013. p. 121–126. doi:10.1109/SIEDS.2013.6549505.
120. Khan A, Bhowmick SS, Bonchi F. Summarizing Static and Dynamic Big Graphs. Proc. VLDB Endow. 2017;10:1981–4. doi:10.14778/3137765.3137825.
121. Khan A, Elnikety S. Systems for Big-graphs. Proc. VLDB Endow. 2014;7:1709–10. doi:10.14778/2733004.2733067.
122. Klann JG, McCoy AB, Wright A, Wattanasin N, Sittig DF, Murphy SN. Health care transformation through collaboration on open-source informatics projects: integrating a medical applications platform, research data repository, and patient summarization. Interact J Med Res. 2013;2:e11. doi:10.2196/ijmr.2454.
123. Klann JG, Szolovits P, Downs SM, Schadow G. Decision support from local data: creating adaptive order menus from past clinician behavior. J Biomed Inform. 2014;48:84–93. doi:10.1016/j.jbi.2013.12.005.
124. Kofler J, Pesenhofer R, Landl G, Sommerfeld-Stur I, Peham C. Monitoring of dairy cow claw health status in 15 herds using the computerised documentation program Claw Manager and digital parameters. Tierarztl Prax Ausg G Grosstiere Nutztiere. 2013;41:31–44.
125. Kourtis G, Caiaffa M, Forte C, Scarlato MI, Macchia L. Retrospective monitoring in the management of persistent asthma. Respir Care. 2011;56:633–43. doi:10.4187/respcare.00796.
126. Kraus S, Castellanos I, Albermann M, Schuettler C, Prokosch H, Staudigel M, Toddenroth D. Using Arden Syntax for the Generation of Intelligent Intensive Care Discharge Letters. Stud Health Technol Inform. 2016;228:471–5.
127. Kushniruk A, Borycki E, Anderson JG, Anderson MM. Combining Two Forms of Simulation to Predict the Potential Impact of Interface Design on Technology-induced Error in Healthcare. In: San Diego, CA, USA: Society for Computer Simulation International; 2008. p. 497–504.
128. Kwon Y, Kang K, Bae C, Chung H, Kim JH. Lifelog agent for human activity pattern analysis on health avatar platform. Healthc Inform Res. 2014;20:69–75. doi:10.4258/hir.2014.20.1.69.
129. Labarre A, Verwer S. Merging Partially Labelled Trees: Hardness and a Declarative Programming Solution. IEEE/ACM Trans. Comput. Biol. Bioinformatics. 2014;11:389–97. doi:10.1109/TCBB.2014.2307200.
130. Lancia G, Carr R, Walenz B, Istrail S. 101 Optimal PDB Structure Alignments: A Branch-and-cut Algorithm for the Maximum Contact Map Overlap Problem. In: New York, NY, USA: ACM; 2001. p. 193–202. doi:10.1145/369133.369199.
131. Landes SJ, Carlson EB, Ruzek JI, Wang D, Hugo E, DeGaetano N, et al. Provider-Driven Development of a Measurement Feedback System to Enhance Measurement-Based Care in VA Mental Health. COGNITIVE AND BEHAVIORAL PRACTICE. 2015;22:87–100. doi:10.1016/j.cbpra.2014.06.004.
132. Lee HJ, Kim JY, Kim SK, Lee JR, Suh CS, Kim SH. Learning Curve Analysis and Surgical Outcomes of Single-port Laparoscopic Myomectomy. J Minim Invasive Gynecol. 2015;22:607–11. doi:10.1016/j.jmig.2015.01.009.
133. Levin RJ. Incidence of thyroid cancer in residents surrounding the Three Mile Island nuclear facility. Laryngoscope. 2008;118:618–28. doi:10.1097/MLG.0b013e3181613ad2.

134. Lezcano L, Sanchez-Alonso S, Sicilia M. Associating clinical archetypes through UMLS Metathesaurus term clusters. J Med Syst. 2012;36:1249–58. doi:10.1007/s10916-010-9586-9.
135. Liabsuetrakul T, Prappre T, Pairot P, Oumudee N, Islam M. Development of a web-based epidemiological surveillance system with health system response for improving maternal and newborn health: Field-testing in Thailand. Health Informatics J. 2017;23:109–23. doi:10.1177/1460458216628312.
136. Lin YL, Guerguerian A, Tomasi J, Laussen P, Trbovich P. Usability of data integration and visualization software for multidisciplinary pediatric intensive care: a human factors approach to assessing technology. BMC Med Inform Decis Mak 2017. doi:10.1186/s12911-017-0520-7.
137. Lin H, Wang Y, Jing L, Chang P. Mockup design of personal health diary app for patients with chronic kidney disease. Stud Health Technol Inform. 2014;201:124–32.
138. Liu W, Chung BC, Wang R, Ng J, Morlet N. A genetic algorithm enabled ensemble for unsupervised medical term extraction from clinical letters. Health Inf Sci Syst. 2015;3:5. doi:10.1186/s13755-015-0013-y.
139. Liu H, Dou D, Jin R, Lependu P, Shah N. Mining Biomedical Ontologies and Data Using RDF Hypergraphs. In: ; 2013. p. 141–146. doi:10.1109/ICMLA.2013.31.
140. Liu Y, Guo Z, Ke X, Za\"ıane OR. Protein Subcellular Localization Prediction with Associative Classification and Multi-class SVM. In: New York, NY, USA: ACM; 2011. p. 493–495. doi:10.1145/2147805.2147880.
141. Liu JM, Wu HW, Chen WS, Lin WC, Chao Y, Lui WY, Whang-Peng J. Integration of computer-assembled digital images and text data as evidence for the oncological record. J Digit Imaging. 2000;13:55–9.
142. Loorak MH, Perin C, Kamal N, Hill M, Carpendale S. TimeSpan: Using Visualization to Explore Temporal Multi-dimensional Data of Stroke Patients. IEEE Trans Vis Comput Graph. 2016;22:409–18. doi:10.1109/TVCG.2015.2467325.
143. Lopez KD, Wilkie DJ, Yao Y, Sousa V, Febretti A, Stifter J, et al. Nurses' Numeracy and Graphical Literacy: Informing Studies of Clinical Decision Support Interfaces. J Nurs Care Qual. 2016;31:124–30. doi:10.1097/NCQ.0000000000000149.
144. Lovis C, Baud RH, Revillard C, Pult L, Borst F, Geissbuhler A. Paragraph-oriented structure for narratives in medical documentation. Stud Health Technol Inform. 2001;84:638–42.
145. Luo Y, Xin Y, Hochberg E, Joshi R, Uzuner O, Szolovits P. Subgraph augmented non-negative tensor factorization (SANTF) for modeling clinical narrative text. J Am Med Inform Assoc. 2015;22:1009–19. doi:10.1093/jamia/ocv016.
146. Luque-Fernandez MA, Zoega H, Valdimarsdottir U, Williams MA. Deconstructing the smoking-preeclampsia paradox through a counterfactual framework. Eur J Epidemiol. 2016;31:613–23. doi:10.1007/s10654-016-0139-5.
147. Madkour M, Song H, Du Jingcheng, Tao C. A representational analysis of a temporal indeterminancy display in clinical events. In: ; 2016. p. 1089–1095. doi:10.1109/BIBM.2016.7822673.
148. Mandal AK, Sarkar A. Formal representation of service interactions for SaaS based applications. In: ; 2014. p. 54–59. doi:10.1109/ICADIWT.2014.6814700.
149. Margusino-Framinan L, Cid-Silva P, Mena-de-Cea A, Sanclaudio-Luhia AI, Castro-Castro JA, Vazquez-Gonzalez G, Martin-Herranz I. Intelligent MONitoring System for antiviral pharmacotherapy in patients with chronic hepatitis C (SiMON-VC). Farm Hosp. 2017;41:68–88. doi:10.7399/fh.2017.41.1.10590.
150. Markazi-Moghaddam N, Arab M, Ravaghi H, Rashidian A, Khatibi T, Zargar Balaye Jame S. A Knowledge Map for Hospital Performance Concept: Extraction and Analysis: A Narrative Review Article. Iran J Public Health. 2016;45:843–54.
151. Martin GS, Tapsell LC, Batterham MJ, Russell KG. Relative bias in diet history measurements: a quality control technique for dietary intervention trials. Public Health Nutr. 2002;5:537–45. doi:10.1079/PHN2002329.
152. Martinez D, Otegi A, Soroa A, Agirre E. Improving search over Electronic Health Records using UMLS-based query expansion through random walks. J Biomed Inform. 2014;51:100–6. doi:10.1016/j.jbi.2014.04.013.
153. Martinez-Costa C, Schulz S. Validating EHR clinical models using ontology patterns. J Biomed Inform. 2017;76:124–37. doi:10.1016/j.jbi.2017.11.001.
154. Matthews SJ. Heterogeneous Compression of Large Collections of Evolutionary Trees. IEEE/ACM Trans. Comput. Biol. Bioinformatics. 2015;12:807–14. doi:10.1109/TCBB.2014.2366756.
155. Mechkour M, Mulhem P, Fourel F, Berrut EFC. PRIME-GC. A medical information retrieval prototype on the Web. In: ; 1997. p. 2–9. doi:10.1109/RIDE.1997.583681.
156. Mendonça EA, Johnson SB, Seol Y, Cimino JJ. Analyzing the Semantics of Patient Data to Rank Records of Literature Retrieval. In: Stroudsburg, PA, USA: Association for Computational Linguistics; 2002. p. 69–76. doi:10.3115/1118149.1118159.
157. Meystre SM, Lee S, Jung CY, Chevrier RD. Common data model for natural language processing based on two existing standard information models: CDA+GrAF. J Biomed Inform. 2012;45:703–10. doi:10.1016/j.jbi.2011.11.018.
158. Milicchio F, Tradigo G, Veltri P, Prosperi M. High-performance Data Structures for De Novo Assembly of Genomes: Cache Oblivious Generic Programming. In: New York, NY, USA: ACM; 2016. p. 657–662. doi:10.1145/2975167.2985691.
159. Mirshahi A, Kohnen T. Scientific evaluation and quality assurance in refractive surgical interventions. Evaluation of the Datagraph med computer program. Ophthalmologe. 2002;99:629–35.

160. Mirzaei M, Truswell AS, Arnett K, Page A, Taylor R, Leeder SR. Cerebrovascular disease in 48 countries: secular trends in mortality 1950-2005. J Neurol Neurosurg Psychiatry. 2012;83:138–45. doi:10.1136/jnnp-2011-300408.
161. Morin P, Herrmann F, Ammann P, Uebelhart B, Rizzoli R. A rapid self-administered food frequency questionnaire for the evaluation of dietary protein intake. Clin Nutr. 2005;24:768–74. doi:10.1016/j.clnu.2005.03.002.
162. Moruzzi M. The new culture of dematerialized health. Recenti Prog Med. 2014;105:407–9. doi:10.1701/1680.18398.
163. Mossel E, Roch S, Steel M. Shrinkage Effect in Ancestral Maximum Likelihood. IEEE/ACM Trans. Comput. Biol. Bioinformatics. 2009;6:126–33. doi:10.1109/TCBB.2008.107.
164. Mozhayskiy V, Miller B, Ma K, Tagkopoulos I. A Scalable Multi-scale Framework for Parallel Simulation and Visualization of Microbial Evolution. In: New York, NY, USA: ACM; 2011. p. 7:1. doi:10.1145/2016741.2016749.
165. Muller R. The CliniCon framework for context representation in electronic patient records. Proc AMIA Annu Fall Symp. 1997:178–82.
166. Murphy CA, Carstens K, Villamayor P. Electronic growth charts: watching our patients grow. AMIA Annu Symp Proc. 2005:1058.
167. Mwaniki P, Ayieko P, Todd J, English M. Assessment of paediatric inpatient care during a multifaceted quality improvement intervention in Kenyan district hospitals--use of prospectively collected case record data. BMC Health Serv Res. 2014;14:312. doi:10.1186/1472-6963-14-312.
168. Myleus A, Stenlund H, Hernell O, Gothefors L, Hammarstrom M, Persson L, Ivarsson A. Early vaccinations are not risk factors for celiac disease. Pediatrics. 2012;130:e63-70. doi:10.1542/peds.2011-2806.
169. Nagar A, Al-Mubaid H, Bettayeb S. Computing Gene Functional Similarity Using Combined Graphs. In: New York, NY, USA: ACM; 2012. p. 1381–1386. doi:10.1145/2245276.2231995.
170. Newell SD, JR, Englert J, Box-Taylor A, Davis KM, Koch KE. Clinical efficiency tools improve stroke management in a rural southern health system. Stroke. 1998;29:1092–8.
171. Ng K, Ghoting A, Steinhubl SR, Stewart WF, Malin B, Sun J. PARAMO: a PARAllel predictive MOdeling platform for healthcare analytic research using electronic health records. J Biomed Inform. 2014;48:160–70. doi:10.1016/j.jbi.2013.12.012.
172. Nielsen F\. A Fielded Wiki for Personality Genetics. In: New York, NY, USA: ACM; 2010. p. 16:1. doi:10.1145/1832772.1832795.
173. Nikfarjam A, Emadzadeh E, Gonzalez G. Towards generating a patient's timeline: extracting temporal relationships from clinical notes. J Biomed Inform. 2013;46 Suppl:S40-7. doi:10.1016/j.jbi.2013.11.001.
174. Nori N, Kashima H, Yamashita K, Ikai H, Imanaka Y. Simultaneous Modeling of Multiple Diseases for Mortality Prediction in Acute Hospital Care. In: New York, NY, USA: ACM; 2015. p. 855–864. doi:10.1145/2783258.2783308.
175. Okamoto K, Tanaka H, Takemura T, Kume N, Kuroda T, Yoshihara H. A hypothesis-generating support system using medical records for clinical knowledge acquisition. In: ; 2012. p. 1130–1133. doi:10.1109/SCIS-ISIS.2012.6505123.
176. Orban K, Edberg A, Erlandsson L. Using a time-geographical diary method in order to facilitate reflections on changes in patterns of daily occupations. Scand J Occup Ther. 2012;19:249–59. doi:10.3109/11038128.2011.620981.
177. O'Sullivan EM. International variation in the incidence of oral and pharyngeal cancer. Community Dent Health. 2008;25:148–53.
178. Pabón MC, Montoya GA, Millán M. Mediation and graph data models for medical data integration. In: ; 2013. p. 1–9. doi:10.1109/CLEI.2013.6670647.
179. Parkin DM, Ferlay J, Curado M, Bray F, Edwards B, Shin H, Forman D. Fifty years of cancer incidence: CI5 I-IX. Int J Cancer. 2010;127:2918–27. doi:10.1002/ijc.25517.
180. Patil R, Karandikar RG. Digital signal preservation approaches of archived biomedical paper records #x2014; A review. In: ; 2016. p. 1–4. doi:10.1109/WECON.2016.7993456.
181. Pawloski P, Cusick D, Amborn L. Development of clinical pharmacy productivity metrics. AMERICAN JOURNAL OF HEALTH-SYSTEM PHARMACY. 2012;69:49–54. doi:10.2146/ajhp110126.
182. Payne TH, Andrews RD, Breeling J, Ben Davoren J, Smith RM, Volpp B. Graphing clinical events in the VA Computerized Patient Record System. JOURNAL OF THE AMERICAN MEDICAL INFORMATICS ASSOCIATION. 1999:1137.
183. Pedreschi D. Social Network Analytics, Data Science Ethics & Privacy-preserving Analytics. In: New York, NY, USA: ACM; 2017. doi:10.1145/3168836.3168841.
184. Penfield W. Physicians take to the field. IDRC Rep. 1992;20:28–30.
185. Pfeiffer KP. Actual state and perspectives of e-health in Austria and international--an overview. Wien Med Wochenschr. 2011;161:334–40. doi:10.1007/s10354-011-0008-5.
186. Pfundner A, Schnoeberg T, Horn J, Boyce RD, Samwald M. Utilizing the Wikidata System to Improve the Quality of Medical Content in Wikipedia in Diverse Languages: A Pilot Study. JOURNAL OF MEDICAL INTERNET RESEARCH 2015. doi:10.2196/jmir.4163.
187. Poon-Chue A, Menendez L, Gerstner MM, Colletti P, Terk M. MRI evaluation of post-operative seromas in extremity soft tissue sarcomas. SKELETAL RADIOLOGY. 1999;28:279–82. doi:10.1007/s002560050516.
188. Pourriat JL, Huet B, Gabry AL, Rolland C, Cupa M. Case summaries in real-time. Accomplishments with a microcomputer. Ann Fr Anesth Reanim. 1982;1:161–5.
189. Poursadegh M, Poursadegh F, Esmaeili M, Bakhshaee M. Epidemiological Survey of Sinonasal Malignancy in North-East Iran. Iran J Otorhinolaryngol. 2015;27:225–9.

190. Powsner SM, Tufte ER. Summarizing clinical psychiatric data. Psychiatr Serv. 1997;48:1458–61. doi:10.1176/ps.48.11.1458.
191. Preece MHW, Hill A, Horswill MS, Karamatic R, Watson MO. Designing observation charts to optimize the detection of patient deterioriation: reliance on the subjective preferences of healthcare professionals is not enough. Aust Crit Care. 2012;25:238–52. doi:10.1016/j.aucc.2012.01.003.
192. Pryor J. A snapshot of rehabilitation referrals in rural New South Wales. Aust Health Rev. 2010;34:204–9. doi:10.1071/AH08713.
193. Rajagopalan MR, Vellaipandiyan S. Big data framework for national E-governance plan. In: ; 2013. p. 1–5. doi:10.1109/ICTKE.2013.6756283.
194. Rakic N. Acute sensorineural hearing loss at the Otorhinolaryngology Department of the General Hospital in Subotica 1991-1996. Med Pregl. 1999;52:44–52.
195. RAMAYYA GP. AXAUDIT - ANESTHETIC AUDIT SYSTEM. INTERNATIONAL JOURNAL OF CLINICAL MONITORING AND COMPUTING. 1992;9:149–58. doi:10.1007/BF01145167.
196. Rassinoux AM, Michel PA, Wagner J, Baud R. Current trends with natural language processing. Medinfo. 1995;8 Pt 2:1657.
197. Ratib O. From multimodality digital imaging to multimedia patient record. Comput Med Imaging Graph. 1994;18:59–65.
198. Reiz B, Csato L. Bayesian Network Classifier for Medical Data Analysis. INTERNATIONAL JOURNAL OF COMPUTERS COMMUNICATIONS & CONTROL. 2009;4:65–72. doi:10.15837/ijccc.2009.1.2414.
199. Rhea S, Weber DJ, Poole C, Cairns C. Risk factors for hospitalization after dog bite injury: a case-cohort study of emergency department visits. Acad Emerg Med. 2014;21:196–203. doi:10.1111/acem.12312.
200. Rizvi SZR, Fong PW. Interoperability of Relationship- and Role-Based Access Control. In: New York, NY, USA: ACM; 2016. p. 231–242. doi:10.1145/2857705.2857706.
201. Rizvi SZR, Fong PW, Crampton J, Sellwood J. Relationship-Based Access Control for an Open-Source Medical Records System. In: New York, NY, USA: ACM; 2015. p. 113–124. doi:10.1145/2752952.2752962.
202. Rocha J. Graph Comparison by Log-Odds Score Matrices with Application to Protein Topology Analysis. IEEE/ACM Trans. Comput. Biol. Bioinformatics. 2011;8:564–9. doi:10.1109/TCBB.2010.59.
203. Ruiz JG, Andrade AD, Hogue C, Karanam C, Akkineni S, Cevallos D, et al. The Association of Graph Literacy With Use of and Skills Using an Online Personal Health Record in Outpatient Veterans. J Health Commun. 2016;21:83–90. doi:10.1080/10810730.2016.1193915.
204. Ryu I, Siio I. TongueDx: A Tongue Diagnosis for Health Care on Smartphones. In: New York, NY, USA: ACM; 2014. p. 25:1. doi:10.1145/2582051.2582076.
205. Salinas O, Cosio G de, Clavel-Arcas C, Montoya J, Serpas M, Moran de Garcia S, Concha-Eastman A. An information system for injuries from external causes (SILEX): a successful project in El Salvador. Rev Panam Salud Publica. 2008;24:390–9.
206. Sauleau EA, Paumier J, Buemi A. Medical record linkage in health information systems by approximate string matching and clustering. BMC Med Inform Decis Mak. 2005;5:32. doi:10.1186/1472-6947-5-32.
207. Schenk RJ, JR, Schenk J. Integration of remote blood glucose meter upload technology into a clinical pharmacist medication therapy management service. J Diabetes Sci Technol. 2011;5:188–91. doi:10.1177/193229681100500126.
208. Schultz SE, Rothwell DM, Chen Z, Tu K. Identifying cases of congestive heart failure from administrative data: a validation study using primary care patient records. Chronic Dis Inj Can. 2013;33:160–6.
209. Schultz SE, Rothwell DM, Chen Z, Tu K. Identifying cases of congestive heart failure from administrative data: a validation study using primary care patient records. Chronic Dis Inj Can. 2013;33:160–6.
210. Sevick MA, Zickmund S, Korytkowski M, Piraino B, Sereika S, Mihalko S, et al. Design, feasibility, and acceptability of an intervention using personal digital assistant-based self-monitoring in managing type 2 diabetes. Contemp Clin Trials. 2008;29:396–409. doi:10.1016/j.cct.2007.09.004.
211. Sharit J, Lisigurski M, Andrade AD, Karanam C, Nazi KM, Lewis JR, Ruiz JG. The Roles of Health Literacy, Numeracy, and Graph Literacy on the Usability of the VA's Personal Health Record by Veterans. J. Usability Studies. 2014;9:173–93.
212. Shaverdian AA, Zhou H, Michailidis G, Jagadish HV. Algebraic Visual Analysis: The Catalano Phone Call Data Set Case Study. In: New York, NY, USA: ACM; 2009. p. 74–82. doi:10.1145/1562849.1562858.
213. Shaw JLV, Cohen A, Konforte D, Binesh-Marvasti T, Colantonio DA, Adeli K. Validity of establishing pediatric reference intervals based on hospital patient data: a comparison of the modified Hoffmann approach to CALIPER reference intervals obtained in healthy children. Clin Biochem. 2014;47:166–72. doi:10.1016/j.clinbiochem.2013.11.008.
214. Sheridan J, Chamberlain K, Dupuis A. Timelining: visualizing experience. QUALITATIVE RESEARCH. 2011;11:552–69. doi:10.1177/1468794111413235.
215. Shibuya T. Geometric Suffix Tree: Indexing Protein 3-D Structures. J. ACM. 2010;57:15:1. doi:10.1145/1706591.1706595.
216. Shigli H, Tejas MH, Narayan L, Desai SD. User Intervention Based Segmentation of Myocardium In Cardiac Cine MRI Images. In: New York, NY, USA: ACM; 2015. p. 684–689. doi:10.1145/2791405.2791545.

217. Shim S, Kim D, Lee D, Lee S, Park J, Lee JJ, et al. Metabolic tumour volume and total lesion glycolysis, measured using preoperative 18F-FDG PET/CT, predict the recurrence of endometrial cancer. BJOG. 2014;121:1097-106; discussion 1106. doi:10.1111/1471-0528.12543.
218. Sills MR, Kwan BM, Yawn BP, Sauer BC, Fairclough DL, Federico MJ, et al. Medical home characteristics and asthma control: a prospective, observational cohort study protocol. EGEMS (Wash DC). 2013;1:1032. doi:10.13063/2327-9214.1032.
219. Sim LLW, Ban KHK, Tan TW, Sethi SK, Loh TP. Development of a clinical decision support system for diabetes care: A pilot study. PLoS One. 2017;12:e0173021. doi:10.1371/journal.pone.0173021.
220. Sittig DF, Murphy DR, Smith MW, Russo E, Wright A, Singh H. Graphical display of diagnostic test results in electronic health records: a comparison of 8 systems. J Am Med Inform Assoc. 2015;22:900–4. doi:10.1093/jamia/ocv013.
221. SMART JF, ROUX M. A MODEL FOR MEDICAL KNOWLEDGE REPRESENTATION APPLICATION TO THE ANALYSIS OF DESCRIPTIVE PATHOLOGY REPORTS. Methods Inf Med. 1995;34:352–60.
222. Sobo EJ, Andriese S, Stroup C, Morgan D, Kurtin P. Developing indicators for emergency medical services (EMS) system evaluation and quality improvement: a statewide demonstration and planning project. Jt Comm J Qual Improv. 2001;27:138–54.
223. Soldes OS, Younger JG, Hirschl RB. Predictors of malignancy in childhood peripheral lymphadenopathy. J Pediatr Surg. 1999;34:1447–52.
224. Sondhi P, Sun J, Tong H, Zhai C. SympGraph: A Framework for Mining Clinical Notes Through Symptom Relation Graphs. In: New York, NY, USA: ACM; 2012. p. 1167–1175. doi:10.1145/2339530.2339712.
225. Sovik S, Skaga NO, Hanoa R, Eken T. Sudden survival improvement in critical neurotrauma: An exploratory analysis using a stratified statistical process control technique. Injury. 2014;45:1722–30. doi:10.1016/j.injury.2014.05.038.
226. Sridhar S, Dhamdhere K, Blelloch G, Halperin E, Ravi R, Schwartz R. Algorithms for Efficient Near-Perfect Phylogenetic Tree Reconstruction in Theory and Practice. IEEE/ACM Trans. Comput. Biol. Bioinformatics. 2007;4:561–71. doi:10.1109/TCBB.2007.1070.
227. Stefansson CG. Map analyses of psychiatric services. The application of a computerized psychiatric case register to geographical analysis. Acta Psychiatr Scand. 1984;70:515–22.
228. Stevens VJ, Rossner J, Greenlick M, Stevens N, Frankel HM, Craddick S. Freedom from fat: a contemporary multi-component weight loss program for the general population of obese adults. J Am Diet Assoc. 1989;89:1254–8.
229. Sumner W2, Truszczynski M, Marek VW. A formal model of family medicine. J Am Board Fam Pract. 1996;9:41–52.
230. Sun K, Goncalves JP, Larminie C, Przulj N. Predicting disease associations via biological network analysis. BMC Bioinformatics 2014. doi:10.1186/1471-2105-15-304.
231. Swartz SH, Cowan TM, DePue J, Goldstein MG. Academic profiling of tobacco-related performance measures in primary care. Nicotine Tob Res. 2002;4 Suppl 1:S38-44. doi:10.1080/14622200210128018.
232. Syalim A, Nishide T, Sakurai K. Securing Provenance of Distributed Processes in an Untrusted Environment. IEICE TRANSACTIONS ON INFORMATION AND SYSTEMS. 2012;E95D:1894–907. doi:10.1587/transinf.E95.D.1894.
233. Szeto LK, Liew AW, Yan H, Tang S. Gene Expression Data Clustering and Visualization Based on a Binary Hierarchical Clustering Framework. In: Darlinghurst, Australia, Australia: Australian Computer Society, Inc; 2003. p. 145–152.
234. Taffe J, Dennerstein L. Menstrual diary data and menopausal transition: methodologic issues. Acta Obstet Gynecol Scand. 2002;81:588–94.
235. Techentin R, Foti D, Li P, Daniel E, Gilbert B, Holmes D, Al-Saffar S. Development of a Semi-synthetic Dataset as a Testbed for Big-Data Semantic Analytics. In: ; 2014. p. 252–253. doi:10.1109/ICSC.2014.45.
236. Tezcan B, Khazaezadeh N, Ash A, Oteng-Ntim E. Social disparity and intrauterine death: from politics to policies. J Obstet Gynaecol. 2011;31:507–8. doi:10.3109/01443615.2011.587910.
237. Tiikkaja S, Sandin S, Hultman CM, Modin B, Malki N, Sparen P. Psychiatric disorder and work life: A longitudinal study of intra-generational social mobility. Int J Soc Psychiatry. 2016;62:156–66. doi:10.1177/0020764015614594.
238. Toner JP, Coddington CC, Doody K, van Voorhis B, Seifer DB, Ball GD, et al. Society for Assisted Reproductive Technology and assisted reproductive technology in the United States: a 2016 update. Fertil Steril. 2016;106:541–6. doi:10.1016/j.fertnstert.2016.05.026.
239. Torkar S, Benedik P, Rajkovic U, Sustersic O, Rajkovic V. Design of a Recommendation System for Adding Support in the Treatment of Chronic Patients. Stud Health Technol Inform. 2016;225:879–80.
240. Troszynski M, Niemiec T, Wilczynska A. Assessment of three-level selective perinatal care based on the analysis of early perinatal death rates and cesarean sections in Poland in 2008. Ginekol Pol. 2009;80:670–7.
241. Truyen Tran, Dinh Phung, Luo W, Venkatesh S. Stabilized sparse ordinal regression for medical risk stratification. KNOWLEDGE AND INFORMATION SYSTEMS. 2015;43:555–82. doi:10.1007/s10115-014-0740-4.

242. Tukiendorf A. Cervix uteri cancer incidence in relation to ethnic situation in Opole province, Poland. Cent Eur J Public Health. 2002;10:88–92.
243. Ugon A, Philippe C, Pietrasz S, Ganascia J, Levy PP. OPTISAS a new method to analyse patients with Sleep Apnea Syndrome. Stud Health Technol Inform. 2008;136:547–52.
244. Valko M, Kveton B, Valizadegan H, Cooper GF, Hauskrecht M. Conditional Anomaly Detection with Soft Harmonic Functions. Proc IEEE Int Conf Data Min. 2011;2011:735–43. doi:10.1109/ICDM.2011.40.
245. Vallejo IL, Herrero HH, Sanz JJ, Martin JJ, Azarola EN. Diagnostic variability in a cohort of patients with multiple admissions in the last two decades. ACTAS ESPANOLAS DE PSIQUIATRIA. 2003;31:18–23.
246. Vani SN. Appropriate technologies for mother and child health care in developing countries. Indian Pediatr. 1989;26:1124–30.
247. Vilares M, Ribadas FJ, Graña J. Approximately Common Patterns in Shared-forests. In: New York, NY, USA: ACM; 2001. p. 73–80. doi:10.1145/502585.502599.
248. Voskoboinik A, Gutman MJ, Croagh D, Bell R, Saunder A, Gribbin J, Kanellis J. Implementation and learning of laproscopic donor nephrectomy by a non-transplant general surgeon with advanced laparoscopic skills. Asian J Endosc Surg. 2011;4:127–32. doi:10.1111/j.1758-5910.2011.00092.x.
249. Vossler DG, Am Haltiner, Schepp SK, Friel PA, Caylor LM, Morgan JD, Doherty MJ. Ictal stuttering - A sign suggestive of psychogenic nonepileptic seizures. Neurology. 2004;63:516–9. doi:10.1212/01.WNL.0000133208.57562.CB.
250. Vossler DG, Haltiner AM, Schepp SK, Friel PA, Caylor LM, Morgan JD, Doherty MJ. Ictal stuttering: a sign suggestive of psychogenic nonepileptic seizures. Neurology. 2004;63:516–9.
251. Vranken R, Coulombier D, Kenyon T, Koosimile B, Mavunga T, Coggin W, Binkin N. Use of a computerized tuberculosis register for automated generation of case finding, sputum conversion, and treatment outcome reports. Int J Tuberc Lung Dis. 2002;6:111–20.
252. Walley JD, McDonald M. Integration of mother and child health services in Ethiopia. Trop Doct. 1991;21:32–5. doi:10.1177/004947559102100113.
253. Wang Z, Chakraborty P, Mekaru SR, Brownstein JS, Ye J, Ramakrishnan N. Dynamic Poisson Autoregression for Influenza-Like-Illness Case Count Prediction. In: New York, NY, USA: ACM; 2015. p. 1285–1294. doi:10.1145/2783258.2783291.
254. Wang JT, Chirn G, Marr TG, Shapiro B, Shasha D, Zhang K. Combinatorial Pattern Discovery for Scientific Data: Some Preliminary Results. In: New York, NY, USA: ACM; 1994. p. 115–125. doi:10.1145/191839.191863.
255. Wang JT, Chirn G, Marr TG, Shapiro B, Shasha D, Zhang K. Combinatorial Pattern Discovery for Scientific Data: Some Preliminary Results. SIGMOD Rec. 1994;23:115–25. doi:10.1145/191843.191863.
256. Wang B, Lin C, Yang I. Constructing a Gene Team Tree in Almost O (N Lg N) Time. IEEE/ACM Trans. Comput. Biol. Bioinformatics. 2014;11:142–53. doi:10.1109/TCBB.2013.150.
257. Wang X, Wang F, Wang J, Qian B, Hu J. Exploring Patient Risk Groups with Incomplete Knowledge. In: ; 2013. p. 1223–1228. doi:10.1109/ICDM.2013.129.
258. Welch JL, Siek KA, Connelly KH, Astroth KS, McManus MS, Scott L, et al. Merging health literacy with computer technology: self-managing diet and fluid intake among adult hemodialysis patients. Patient Educ Couns. 2010;79:192–8. doi:10.1016/j.pec.2009.08.016.
259. White D, Choi H, Peloquin C, Zhu Y, Zhang Y. Secular trend of adhesive capsulitis. Arthritis Care Res (Hoboken). 2011;63:1571–5. doi:10.1002/acr.20590.
260. Wilhelms J, van Gelder A. Octrees for Faster Isosurface Generation. ACM Trans. Graph. 1992;11:201–27. doi:10.1145/130881.130882.
261. Winter A, Brigl B, Funkat G, Haeber A, Heller O, Wendt T. 3LGM(2)-Modeling to support management of health information systems. Int J Med Inform. 2007;76:145–50. doi:10.1016/j.ijmedinf.2006.07.007.
262. Wongsuphasawat K, Gotz D. Exploring Flow, Factors, and Outcomes of Temporal Event Sequences with the Outflow Visualization. IEEE Trans Vis Comput Graph. 2012;18:2659–68. doi:10.1109/TVCG.2012.225.
263. Wu T, Moulton V, Steel M. Refining Phylogenetic Trees Given Additional Data: An Algorithm Based on Parsimony. IEEE/ACM Trans. Comput. Biol. Bioinformatics. 2009;6:118–25. doi:10.1109/TCBB.2008.100.
264. WU Y, Zhu X, Li L, Fan W, Jin R, Zhang X. Mining Dual Networks: Models, Algorithms, and Applications. ACM Trans. Knowl. Discov. Data. 2016;10:40:1. doi:10.1145/2785970.
265. Xie W, Wu J. Mining positive and negative weighted association rules in medical records without user-specified weights based on HITS model. In: ; 2010. p. 2325–2329. doi:10.1109/BMEI.2010.5639578.
266. Xie C, Yang P, Yang Y. Open Knowledge Accessing Method in IoT-based Hospital Information System for Medical Record Enrichment. IEEE Access. 2018;PP:1. doi:10.1109/ACCESS.2018.2810837.
267. Yamin A, Khan SA, Yasin UU. Automated system of hess screen for diagnosis of paralytic strabismus using computer aided diagnosis. In: ; 2013. p. 300–305. doi:10.1109/IST.2013.6729710.
268. Yoon S, Cohen B, Cato KD, Liu J, Larson EL. Visualization of Data Regarding Infections Using Eye Tracking Techniques. J Nurs Scholarsh. 2016;48:244–53. doi:10.1111/jnu.12204.
269. Yoon D, Park I, Schuemie MJ, Park MY, Kim JH, Park RW. A quantitative method for assessment of prescribing patterns using electronic health records. PLoS One. 2013;8:e75214. doi:10.1371/journal.pone.0075214.

270. Zamora M, Baradad M, Amado E, Cordomí S, Limón E, Ribera J, et al. Characterizing chronic disease and polymedication prescription patterns from electronic health records. In: ; 2015. p. 1–9. doi:10.1109/DSAA.2015.7344870.
271. Zenios S. Information Technology in Health Care Systems: Barriers to Adoption. In: ; 2006. p. 52. doi:10.1109/ICCGI.2006.35.
272. Zhang H, Mehotra S, Liebovitz D, Gunter CA, Malin B. Mining Deviations from Patient Care Pathways via Electronic Medical Record System Audits. ACM Trans. Manage. Inf. Syst. 2013;4:17:1. doi:10.1145/2544102.
273. Zhang M, Zhang H, Tjandra D, Wong STC. DBMap: a space-conscious data visualization and knowledge discovery framework for biomedical data warehouse. IEEE Transactions on Information Technology in Biomedicine. 2004;8:343–53. doi:10.1109/TITB.2004.832550.
274. Zhao S. Mining Medical Causality for Diagnosis Assistance. In: New York, NY, USA: ACM; 2017. p. 841. doi:10.1145/3018661.3022752.
275. Zheng Z, Wan X. Graph-Based Multi-Modality Learning for Clinical Decision Support. In: New York, NY, USA: ACM; 2016. p. 1945–1948. doi:10.1145/2983323.2983880.
276. Zirkind G. Genetic Database Optimization: How Data Inspection and Consideration, Provides for Index Compression and Record Access Optimization of Genetic Databases. In: New York, NY, USA: ACM; 2006. p. 68–76. doi:10.1145/1125170.1125194.
277. ZWEIGENBAUM P. MENELAS - AN ACCESS SYSTEM FOR MEDICAL RECORDS USING NATURAL-LANGUAGE. Comput Methods Programs Biomed. 1994;45:117–20. doi:10.1016/0169-2607(94)90029-9.
278. Albarakati N, Obradovic Z. Disease-Based Clustering of Hospital Admission: Disease Network of Hospital Networks Approach. In: ; 2017. p. 636–641. doi:10.1109/CBMS.2017.87.
279. Andersen A. An implementation of secure multi-party computations to preserve privacy when processing EMR data. In: ; 2013. p. 381–384. doi:10.1109/PST.2013.6596096.
280. Andersen A. SNOOP: Privacy Preserving Middleware for Secure Multi-party Computations. In: New York, NY, USA: ACM; 2014. p. 8:1. doi:10.1145/2677017.2677025.
281. Andersen A, Yigzaw KY, Karlsen R. Privacy preserving health data processing. In: ; 2014. p. 225–230. doi:10.1109/HealthCom.2014.7001845.
282. Brunson JC, Laubenbacher RC. Applications of network analysis to routinely collected health care data: a systematic review. JOURNAL OF THE AMERICAN MEDICAL INFORMATICS ASSOCIATION. 2018;25:210–21. doi:10.1093/jamia/ocx052.
283. Chen J, Poon J, Poon SK, Xu L, Sze DMY. Mining Symptom-Herb Patterns from Patient Records Using Tripartite Graph. Evid Based Complement Alternat Med. 2015;2015:435085. doi:10.1155/2015/435085.
284. Finney JM, Walker AS, Peto TEA, Wyllie DH. An efficient record linkage scheme using graphical analysis for identifier error detection. BMC Med Inform Decis Mak. 2011;11:7. doi:10.1186/1472-6947-11-7.
285. Goodwin TR, Harabagiu SM. Medical Question Answering for Clinical Decision Support. Proc ACM Int Conf Inf Knowl Manag. 2016;2016:297–306. doi:10.1145/2983323.2983819.
286. Goodwin TR, Harabagiu SM. Knowledge Representations and Inference Techniques for Medical Question Answering. ACM TRANSACTIONS ON INTELLIGENT SYSTEMS AND TECHNOLOGY 2018. doi:10.1145/3106745.
287. Hanzlicek P, Spidlen J, Heroutova H, Nagy M. User interface of MUDR electronic health record. Int J Med Inform. 2005;74:221–7. doi:10.1016/j.ijmedinf.2004.06.003.
288. Herskovic JR, Subramanian D, Cohen T, Bozzo-Silva PA, Bearden CF, Bernstam EV. Graph-based signal integration for high-throughput phenotyping. BMC Bioinformatics. 2012;13 Suppl 13:S2. doi:10.1186/1471-2105-13-S13-S2.
289. Honglan L, Xiaona Q, Bin F. The Symptoms and Pathogenesis Entity Recognition of TCM Medical Records Based on CRF. In: ; 2015. p. 1479–1484. doi:10.1109/UIC-ATC-ScalCom-CBDCom-IoP.2015.267.
290. Huet B, Artigou JY, Poirier J, Blain G. Meta-modelling: the appropriate solution for a family of applications. Stud Health Technol Inform. 2001;84:623–7.
291. Huet B, Pourriat JL, Martin J, Cupa M. An automaton computer program for a microcomputerized real-time (thesaurus based) abstract medical record. Comput Programs Biomed. 1982;15:117–23.
292. Jeunemaitre X, Degoulet P, Morice V, Chatellier G, Devries C, Plouin PF, et al. Testing an expert system for hypertension. Arch Mal Coeur Vaiss. 1986;79:808–12.
293. Ji X, Ae Chun S, Geller J. Predicting Comorbid Conditions and Trajectories using Social Health Records. IEEE Trans Nanobioscience 2016. doi:10.1109/TNB.2016.2564299.
294. Kavuluru R, Han S, Harris D. Unsupervised Extraction of Diagnosis Codes from EMRs Using Knowledge-Based and Extractive Text Summarization Techniques. Adv Artif Intell (2013). 2013;7884:77–88. doi:10.1007/978-3-642-38457-8_7.
295. Kerschberger B, Hilderbrand K, Boulle AM, Coetzee D, Goemaere E, Azevedo V de, van Cutsem G. The effect of complete integration of HIV and TB services on time to initiation of antiretroviral therapy: a before-after study. PLoS One. 2012;7:e46988. doi:10.1371/journal.pone.0046988.
296. Koopman B. Semantic Search As Inference: Applications in Health Informatics. SIGIR Forum. 2014;48:116–7. doi:10.1145/2701583.2701601.
297. Koopman B, Zuccon G, Bruza P, Sitbon L, Lawley M. Graph-based Concept Weighting for Medical Information Retrieval. In: New York, NY, USA: ACM; 2012. p. 80–87. doi:10.1145/2407085.2407096.

298. Mondal S, Mukherjee N. Mobile-assisted remote healthcare delivery. In: ; 2016. p. 630–635. doi:10.1109/PDGC.2016.7913199.
299. Monsen KA, Banerjee A, Das P. Discovering client and intervention patterns in home visiting data. West J Nurs Res. 2010;32:1031–54. doi:10.1177/0193945910370970.
300. Muller R, Sergl M, Nauerth U, Schoppe D, Pommerening K, Dittrich HM. THEMPO: a knowledge-based system for therapy planning in pediatric oncology. Comput Biol Med. 1997;27:177–200.
301. Ni J, Fei H, Fan W, Zhang X. Automated Medical Diagnosis by Ranking Clusters Across the Symptom-Disease Network. In: ; 2017. p. 1009–1014. doi:10.1109/ICDM.2017.130.
302. O'Neil M, Payne C, Read J. Read Codes Version 3: a user led terminology. Methods Inf Med. 1995;34:187–92.
303. Pietrzyk PM. Free text analysis. Int J Biomed Comput. 1995;39:139–44.
304. Săcărea C, Şotropa D, Troancă D. Symptoms investigation by means of formal concept analysis for enhancing medical diagnoses. In: ; 2017. p. 1–5. doi:10.23919/SOFTCOM.2017.8115588.
305. Soulakis ND, Carson MB, Lee YJ, Schneider DH, Skeehan CT, Scholtens DM. Visualizing collaborative electronic health record usage for hospitalized patients with heart failure. J Am Med Inform Assoc. 2015;22:299–311. doi:10.1093/jamia/ocu017.
306. Zhang Z, Wang H, Wang C, Fang H. Cluster-based Epidemic Control Through Smartphone-based Body Area Networks. IEEE Trans Parallel Distrib Syst. 2015;26:681–90. doi:10.1109/TPDS.2014.2313331.
307. Adamusiak T, Shimoyama N, Shimoyama M. Next generation phenotyping using the unified medical language system. JMIR Med Inform. 2014;2:e5. doi:10.2196/medinform.3172.
308. Alvarez JM, Polo L, Jimenez W, Abella P, Labra JE. Application of the Spreading Activation Technique for Recommending Concepts of Well-known Ontologies in Medical Systems. In: New York, NY, USA: ACM; 2011. p. 626–635. doi:10.1145/2147805.2147913.
309. Antonakakis M, Dimitriadis SI, Zervakis M, Micheloyannis S, Rezaie R, Babajani-Feremi A, et al. Altered cross-frequency coupling in resting-state MEG after mild traumatic brain injury. Int J Psychophysiol. 2016;102:1–11. doi:10.1016/j.ijpsycho.2016.02.002.
310. Arantes WMd, Verdier C. Public health alert system for health networks: application to cardiology. In: ; 2005. p. 151–154. doi:10.1109/CIC.2005.1588057.
311. Athreya AP, Ngiam KY, Luo Z, Tai ES, Kalbarczyk Z, Iyer RK. Towards Longitudinal Analysis of a Population's Electronic Health Records Using Factor Graphs. In: ; 2016. p. 79–86.
312. Dhulekar N, Oztan B, Yener B, Bingol HO, Irim G, Aktekin B, Aykut-Bingöl C. Graph-theoretic Analysis of Epileptic Seizures on Scalp EEG Recordings. In: New York, NY, USA: ACM; 2014. p. 155–163. doi:10.1145/2649387.2649423.
313. Frazier GF. An Incremental Algorithm for Building Temporal Quadtrees. In: New York, NY, USA: ACM; 1993. p. 446–452. doi:10.1145/170791.170896.
314. Gerson CD, Gerson M. Technical report: an ePRO patient reported outcome program for the evaluation of patients with irritable bowel syndrome. Neurogastroenterol Motil. 2014;26:290–4. doi:10.1111/nmo.12255.
315. Ghosh P, Kalyanaraman A. A Fast Sketch-based Assembler for Genomes. In: New York, NY, USA: ACM; 2016. p. 241–250. doi:10.1145/2975167.2975192.
316. Gopakumar S, Tran T, Nguyen TD, Phung D, Venkatesh S. Stabilizing high-dimensional prediction models using feature graphs. IEEE J Biomed Health Inform. 2015;19:1044–52. doi:10.1109/JBHI.2014.2353031.
317. Heer J, Perer A. Orion: A system for modeling, transformation and visualization of multidimensional heterogeneous networks. INFORMATION VISUALIZATION. 2014;13:111–33. doi:10.1177/1473871612462152.
318. Jing X, Cimino JJ. A complementary graphical method for reducing and analyzing large data sets. Case studies demonstrating thresholds setting and selection. Methods Inf Med. 2014;53:173–85. doi:10.3414/ME13-01-0075.
319. Jupin J, Shi JY. Identity Tracking in Big Data: Preliminary Research Using In-Memory Data Graph Models for Record Linkage and Probabilistic Signature Hashing for Approximate String Matching in Big Health and Human Services Databases. In: New York, NY, USA: ACM; 2014. p. 20:1. doi:10.1145/2640087.2644170.
320. Lieberman MD, Taheri S, Guo w, Mirrashed F, Yahav I, Aris A, Shneiderman B. Visual Exploration Across Biomedical Databases. IEEE/ACM Trans. Comput. Biol. Bioinformatics. 2011;8:536–50. doi:10.1109/TCBB.2010.1.
321. Luz S, Kane B. Classification of Patient Case Discussions Through Analysis of Vocalisation Graphs. In: New York, NY, USA: ACM; 2009. p. 107–114. doi:10.1145/1647314.1647334.
322. Mattson DC, Yang J. The Child Therapy Tracking System (CTTS): A model for an expressive therapy electronic health record (EHR). ARTS IN PSYCHOTHERAPY. 2013;40:509–18. doi:10.1016/j.aip.2013.08.002.
323. Muller R. The CliniCon framework for context representation in electronic patient records. JOURNAL OF THE AMERICAN MEDICAL INFORMATICS ASSOCIATION. 1997:178–82.
324. Noren GN, Hopstadius J, Bate A, Star K, Edwards IR. Temporal pattern discovery in longitudinal electronic patient records. DATA MINING AND KNOWLEDGE DISCOVERY. 2010;20:361–87. doi:10.1007/s10618-009-0152-3.
325. Nose Y, Akazawa K, Watanabe Y, Yokota M, Okamura S, Maehara Y, Sugimachi K. Cancer registration using case history database in hospital information system. Jpn Hosp. 1988;7:21–8.
326. Ogushi Y, Haruki Y, Okada Y, Takahashi M, Shimizu M, Izumi Y, et al. Development and evaluation of regional health database systems. Stud Health Technol Inform. 1998;52 Pt 2:1297–300.

327. Oladimeji EA, Chung L, Jung HT, Kim J. Managing Security and Privacy in Ubiquitous eHealth Information Interchange. In: New York, NY, USA: ACM; 2011. p. 26:1. doi:10.1145/1968613.1968645.
328. Onimura N, Yamashita T, Nakayama N, Soejima H, Hirokawa S. Generation of Sentence Template Graph from SOAP Format Medical Documents. In: ; 2016. p. 159–162. doi:10.1109/CSCI.2016.0037.
329. Oshiro T, Oshiro H, Tanimizu M. Effective methods of organizing complex information in advanced cancer patients. Gan To Kagaku Ryoho. 2014;41 Suppl 1:26–9.
330. Osop H, Sahama T. Electronic health records: Improvement to healthcare decision-making. In: ; 2016. p. 1–6. doi:10.1109/HealthCom.2016.7749474.
331. Poscia R, Ghio S, D'Alto M, Vitulo P, Mule M, Albera C, et al. 'Real-life' information on pulmonary arterial hypertension: the iPHnet Project. Curr Med Res Opin. 2014;30:2409–14. doi:10.1185/03007995.2014.960514.
332. Staubert S, Schaaf M, Jahn F, Brandner R, Winter A. Modeling Interoperable Information Systems with 3LGM(2) and IHE. Methods Inf Med. 2015;54:398–405. doi:10.3414/ME14-02-0027.
333. Techentin R, Sauver JS, Huddleston J, Gilbert B, Holmes D. Lessons learned from the semantic translation of healthcare data. In: ; 2014. p. 513–518. doi:10.1109/HealthCom.2014.7001895.
334. Whyatt CP, Torres EB. The Social-dance: Decomposing Naturalistic Dyadic Interaction Dynamics to the 'Micro-level'. In: New York, NY, USA: ACM; 2017. p. 24:1. doi:10.1145/3077981.3078055.
