# Supplementary material for: Graph-Representation of Patient Data: a Systematic Literature Review
Source: J Med Syst. 2020 Mar 12;44(4):86. doi: 10.1007/s10916-020-1538-4 (PMC7067737; doi:10.1007/s10916-020-1538-4)
Supplement: Supplementary file 6 — (PDF 720 kb) [file 10916_2020_1538_MOESM6_ESM.pdf]

Title: Graph-Representation of Patient Data
A Systematic Literature Review

Journal: Journal of Medical Systems

Authors:

Jens Schrodt, jens.schrodt@med.uni-heidelberg.de Universitätsklinikum Heidelberg Institut für Medizinische Biometrie und Informatik, Im Neuenheimer Feld 130.3, 69120 Heidelberg, Germany, orcid: 0000-0002-9768-4781

Aleksei Dudchenko, Aleksei.Dudchenko@med.uni-heidelberg.de Universitätsklinikum Heidelberg Institut für Medizinische Biometrie und Informatik, Im Neuenheimer Feld 130.3, 69120 Heidelberg, Germany

Petra Knaup, petra.knaup@med.uni-heidelberg.de Universitätsklinikum Heidelberg Institut für Medizinische Biometrie und Informatik, Im Neuenheimer Feld 130.3, 69120 Heidelberg, Germany

Matthias Ganzinger, matthias.ganzinger@med.uni-heidelberg.de Universitätsklinikum Heidelberg Institut für Medizinische Biometrie und Informatik, Im Neuenheimer Feld 130.3, 69120 Heidelberg, Germany

Suppl. Table 4: papers, which were excluded in second round (full-text analysis)

Author, editor or organization	Title	Year	Ref.
Allali, Julien; Sagot, Marie-France	A New Distance for High Level RNA Secondary Structure Comparison	2005	[1]
Bassoe, Carl-Fredrik	Representing health, disorder and their transitions by digraphs	2008	[2]
CAMPBELL, K. E.; DAS, A. K.; MUSEN, M. A.	A LOGICAL FOUNDATION FOR REPRESENTATION OF CLINICAL-DATA	1994	[3]
Chan, Derek J.; Furner, Virginia; Smith, Don E.; Dronavalli, Mithilesh; Bopage, Rohan I.; Post, Jeffrey J.; Bhardwaj, Anjali K.	Non-AIDS complexity amongst patients living with HIV in Sydney: risk factors and health outcomes	2018	[4]
Choi, Edward; Bahadori, Mohammad Taha; Le Song; Stewart, Walter F.; Sun, Jimeng	GRAM: Graph-based Attention Model for Healthcare Representation Learning	2017	[5]
da Silva Klahr, Patricia; Correa Coronel, Christian; Cabral Robinson, Caroline; Fonseca, Joao Marcelo; Dias Flores, Cecilia; Della Mea Plentz, Rodrigo	Influence Diagram As a Support Tool for Clinical Decisions In Cardiopulmonary And Metabolic Rehabilitation	2015	[6]
Datla, V.; Hasan, S. A.; Qadir, A.; Lee, K.; Ling, Y.; Liu, J.; Farri, O.	Automated clinical diagnosis: The role of content in various sections of a clinical document	2017	[7]
Edsberg, Ole; Nordbo, Stein Jakob; Vinnes, Erik; Nytro, Oystein	Design and evaluation of a temporal, graph-based language for querying collections of patient histories	2007	[8]
Giani, U.; Brascio, G.; Bruzzese, D.; Garzillo, C.; Vigilante, S.	Emotional and cognitive information processing in web-based medical education	2007	[9]
Goldstein, Ayelet; Shahar, Yuval	An automated knowledge-based textual summarization system for longitudinal, multivariate clinical data	2016	[10]
Goodwin, T.; Harabagiu, S. M.	Automatic Generation of a Qualified Medical Knowledge Graph and Its Usage for Retrieving Patient Cohorts from Electronic Medical Records	2013	[11]
Gortzis, L. G.; Nikiforidis, G.	Tracing and cataloguing knowledge in an e-health cardiology environment	2008	[12]
Harabagiu, S.	Big mechanisms for processing big data in medical informatics	2015	[13]

Harris, Julie	Next Generation Linkage Management System	2013	[14]
Jiang, Jingchi; Li, Xueli; Zhao, Chao; Guan, Yi; Yu, Qiubin	Learning and inference in knowledge-based probabilistic model for medical diagnosis	2017	[15]
Kamkar, I.; Gupta, S.; Li, Cheng; Phung, D.; Venkatesh, S.	Stable clinical prediction using graph support vector machines	2016	[16]
Manzi de Arantes, Wilmondes Jr; Verdier, Christine	Defining quality-measurable medical alerts from incomplete data through fuzzy linguistic variables and modifiers	2010	[17]
Meamarzadeh, H.; Khayyambashi, M. R.; Saraee, M. H.	Extracting Temporal Rules from Medical Data	2009	[18]
Norén, G. Niklas; Bate, Andrew; Hopstadius, Johan; Star, Kristina; Edwards, I. Ralph	Temporal Pattern Discovery for Trends and Transient Effects: Its Application to Patient Records	2008	[19]
Osler, Merete; Prescott, Eva; Wium-Andersen, Ida Kim; Ibfelt, Else Helene; Jorgensen, Martin Balslev; Andersen, Per Kragh; Jorgensen, Terese Sara Hoj; Wium-Andersen, Marie Kim; Martensson, Solvej	The Impact of Comorbid Depression on Educational Inequality in Survival after Acute Coronary Syndrome in a Cohort of 83 062 Patients and a Matched Reference Population	2015	[20]
Pathak, J.; Kiefer, R. C.; Chute, C. G.	Mining drug-drug interaction patterns from linked data: A case study for Warfarin, Clopidogrel, and Simvastatin	2013	[21]
Paulson, Erin H.; Gerberich, Susan Goodwin; Alexander, Bruce H.; Ryan, Andrew; Renier, Colleen M.; Zhang, Xueying; French, L. Ronald; Masten, Ann S.; Carlson, Kathleen Ferguson	Fall-related injuries among agricultural household members: Regional Rural Injury Study II (RRIS-II)	2006	[22]
Rathe, J. C.; Elliott, P. F.	A cancer registry for the community radiation center	1979	[23]
Rodnick, J. E.	The use of automated ambulatory medical records	1977	[24]
Rothwell, D. J.; Cote, R. A.; Cordeau, J. P.; Boisvert, M. A.	Developing a standard data structure for medical language--the SNOMED proposal	1993	[25]
Rotmensch, Maya; Halpern, Yoni; Tlimat, Abdulhakim; Horng, Steven; Sontag, David	Learning a Health Knowledge Graph from Electronic Medical Records	2017	[26]
Sato, D.; Freitas, A.	Understanding quality in data from hospital diagnosis and procedures: A statistical characterization	2010	[27]
Shahn, Zach; Ryan, Patrick; Madigan, David	Predicting Health Outcomes from High-Dimensional Longitudinal Health Histories Using Relational Random Forests	2015	[28]
Spidlen, Josef; Hanzlicek, Petr; Riha, Antonin; Zvarova, Jana	Flexible information storage in MUDR(II) EHR	2006	[29]
Wang, Fei; Liu, Chuanren; Wang, Yajuan; Hu, Jianying; Yu, Guoqiang	A Graph Based Methodology for Temporal Signature Identification from HER	2015	[30]
Wang, Sen; Chang, Xiaojun; Li, Xue; Long, Guodong; Yao, Lina; Sheng, Quan Z.	Diagnosis Code Assignment Using Sparsity-Based Disease Correlation Embedding	2016	[31]
Wiesner, Martin; Pfeifer, Daniel	Adapting Recommender Systems to the Requirements of Personal Health Record Systems	2010	[32]
Yamashita, Takanori; Onimura, Naoya; Soejima, Hidehisa; Nakashima, Naoki; Hirokawa, Sachio	Graph Clustering System for Text-Based Records in a Clinical Pathway	2017	[33]
Yesha, R.; Gangopadhyay, A.; Siegel, E.	A graph-based method for analyzing electronic medical records	2015	[34]
Yin, S.; Chen, D.; Le J	Deep Neural Network Based on Translation Model for Diabetes Knowledge Graph	2017	[35]
Yousefi, A.; Mastouri, N.; Sartipi, K.	Scenario-oriented information extraction from electronic health records	2009	[36]
Zhang, Ping; Wang, Fei; Hu, Jianying; Sorrentino, Robert	Towards personalized medicine: leveraging patient similarity and drug similarity analytics	2014	[37]
Zhou, Xiaohua; Han, Hyoil; Chankai, Isaac; Prestrud, Ann; Brooks, Ari	Approaches to Text Mining for Clinical Medical Records	2006	[38]

References

1. Allali J, Sagot M. A New Distance for High Level RNA Secondary Structure Comparison. IEEE/ACM Trans. Comput. Biol. Bioinformatics. 2005;2:3–14. doi:10.1109/TCBB.2005.2.
2. Bassoe C. Representing health, disorder and their transitions by digraphs. Stud Health Technol Inform. 2008;136:133–8.
3. CAMPBELL KE, DAS AK, MUSEN MA. A LOGICAL FOUNDATION FOR REPRESENTATION OF CLINICAL-DATA. JOURNAL OF THE AMERICAN MEDICAL INFORMATICS ASSOCIATION. 1994;1:218–32. doi:10.1136/jamia.1994.95236154.
4. Chan DJ, Furner V, Smith DE, Dronavalli M, Bopage RI, Post JJ, Bhardwaj AK. Non-AIDS complexity amongst patients living with HIV in Sydney: risk factors and health outcomes. AIDS Res Ther. 2018;15:6. doi:10.1186/s12981-018-0193-z.
5. Choi E, Bahadori MT, Le Song, Stewart WF, Sun J. GRAM: Graph-based Attention Model for Healthcare Representation Learning. In: New York, NY, USA: ACM; 2017. p. 787–795. doi:10.1145/3097983.3098126.
6. da Silva Klahr P, Correa Coronel C, Cabral Robinson C, Fonseca JM, Dias Flores C, Della Mea Plentz R. Influence Diagram As a Support Tool for Clinical Decisions In Cardiopulmonary And Metabolic Rehabilitation. Stud Health Technol Inform. 2015;216:290–4.
7. Datla V, Hasan SA, Qadir A, Lee K, Ling Y, Liu J, Farri O. Automated clinical diagnosis: The role of content in various sections of a clinical document. In: ; 2017. p. 1004–1011. doi:10.1109/BIBM.2017.8217794.
8. Edsberg O, Nordbo SJ, Vinnes E, Nytro O. Design and evaluation of a temporal, graph-based language for querying collections of patient histories. Stud Health Technol Inform. 2007;129:402–6.
9. Giani U, Brascio G, Bruzzese D, Garzillo C, Vigilante S. Emotional and cognitive information processing in web-based medical education. J Biomed Inform. 2007;40:332–42. doi:10.1016/j.jbi.2006.11.004.
10. Goldstein A, Shahar Y. An automated knowledge-based textual summarization system for longitudinal, multivariate clinical data. J Biomed Inform. 2016;61:159–75. doi:10.1016/j.jbi.2016.03.022.
11. Goodwin T, Harabagiu SM. Automatic Generation of a Qualified Medical Knowledge Graph and Its Usage for Retrieving Patient Cohorts from Electronic Medical Records. In: ; 2013. p. 363–370. doi:10.1109/ICSC.2013.68.
12. Gortzis LG, Nikiforidis G. Tracing and cataloguing knowledge in an e-health cardiology environment. J Biomed Inform. 2008;41:217–23. doi:10.1016/j.jbi.2007.09.001.
13. Harabagiu S. Big mechanisms for processing big data in medical informatics. In: ; 2015. p. 1–2. doi:10.1109/SPMB.2015.7405440.
14. Harris J. Next Generation Linkage Management System. In: Darlinghurst, Australia, Australia: Australian Computer Society, Inc; 2013. p. 7–12.
15. Jiang J, Li X, Zhao C, Guan Y, Yu Q. Learning and inference in knowledge-based probabilistic model for medical diagnosis. KNOWLEDGE-BASED SYSTEMS. 2017;138:58–68. doi:10.1016/j.knosys.2017.09.030.
16. Kamkar I, Gupta S, Li C, Phung D, Venkatesh S. Stable clinical prediction using graph support vector machines. In: ; 2016. p. 3332–3337. doi:10.1109/ICPR.2016.7900148.
17. Manzi de Arantes, Wilmondes Jr, Verdier C. Defining quality-measurable medical alerts from incomplete data through fuzzy linguistic variables and modifiers. IEEE Trans Inf Technol Biomed. 2010;14:916–22. doi:10.1109/TITB.2009.2020063.
18. Meamarzadeh H, Khayyambashi MR, Saraee MH. Extracting Temporal Rules from Medical Data. In: ; 2009. p. 327–331. doi:10.1109/ICCTD.2009.72.
19. Norén GN, Bate A, Hopstadius J, Star K, Edwards IR. Temporal Pattern Discovery for Trends and Transient Effects: Its Application to Patient Records. In: New York, NY, USA: ACM; 2008. p. 963–971. doi:10.1145/1401890.1402005.
20. Osler M, Prescott E, Wium-Andersen IK, Ibfelt EH, Jorgensen MB, Andersen PK, et al. The Impact of Comorbid Depression on Educational Inequality in Survival after Acute Coronary Syndrome in a Cohort of 83 062 Patients and a Matched Reference Population. PLoS One. 2015;10:e0141598. doi:10.1371/journal.pone.0141598.
21. Pathak J, Kiefer RC, Chute CG. Mining drug-drug interaction patterns from linked data: A case study for Warfarin, Clopidogrel, and Simvastatin. In: ; 2013. p. 23–30. doi:10.1109/BIBM.2013.6732595.
22. Paulson EH, Gerberich SG, Alexander BH, Ryan A, Renier CM, Zhang X, et al. Fall-related injuries among agricultural household members: Regional Rural Injury Study II (RRIS-II). J Occup Environ Med. 2006;48:959–68. doi:10.1097/01.jom.0000225110.89501.a3.
23. Rathe JC, Elliott PF. A cancer registry for the community radiation center. Cancer. 1979;43:198–204.
24. Rodnick JE. The use of automated ambulatory medical records. J Fam Pract. 1977;5:253–64.
25. Rothwell DJ, Cote RA, Cordeau JP, Boisvert MA. Developing a standard data structure for medical language--the SNOMED proposal. Proc Annu Symp Comput Appl Med Care. 1993:695–9.
26. Rotmensch M, Halpern Y, Tlimat A, Horng S, Sontag D. Learning a Health Knowledge Graph from Electronic Medical Records. Sci Rep. 2017;7:5994. doi:10.1038/s41598-017-05778-z.
27. Sato D, Freitas A. Understanding quality in data from hospital diagnosis and procedures: A statistical characterization. In: ; 2010. p. 1–4.

28. Shahn Z, Ryan P, Madigan D. Predicting Health Outcomes from High-Dimensional Longitudinal Health Histories Using Relational Random Forests. STATISTICAL ANALYSIS AND DATA MINING. 2015;8:128–36. doi:10.1002/sam.11268.
29. Spidlen J, Hanzlicek P, Riha A, Zvarova J. Flexible information storage in MUDR(II) EHR. Int J Med Inform. 2006;75:201–8. doi:10.1016/j.ijmedinf.2005.07.044.
30. Wang F, Liu C, Wang Y, Hu J, Yu G. A Graph Based Methodology for Temporal Signature Identification from HER: RETRACTED ARTICLE - NICHT BENUTZEN, ABGELEHNTER ARTIKEL. AMIA Annu Symp Proc. 2015;2015:1269–78.
31. Wang S, Chang X, Li X, Long G, Yao L, Sheng QZ. Diagnosis Code Assignment Using Sparsity-Based Disease Correlation Embedding. IEEE TRANSACTIONS ON KNOWLEDGE AND DATA ENGINEERING. 2016;28:3191–202. doi:10.1109/TKDE.2016.2605687.
32. Wiesner M, Pfeifer D. Adapting Recommender Systems to the Requirements of Personal Health Record Systems. In: New York, NY, USA: ACM; 2010. p. 410–414. doi:10.1145/1882992.1883053.
33. Yamashita T, Onimura N, Soejima H, Nakashima N, Hirokawa S. Graph Clustering System for Text-Based Records in a Clinical Pathway. Stud Health Technol Inform. 2017;245:649–52.
34. Yesha R, Gangopadhyay A, Siegel E. A graph-based method for analyzing electronic medical records. In: ; 2015. p. 1036–1041. doi:10.1145/2808797.2808806.
35. Yin S, Chen D, Le J. Deep Neural Network Based on Translation Model for Diabetes Knowledge Graph. In: ; 2017. p. 318–323. doi:10.1109/CBD.2017.62.
36. Yousefi A, Mastouri N, Sartipi K. Scenario-oriented information extraction from electronic health records. In: ; 2009. p. 1–5. doi:10.1109/CBMS.2009.5255451.
37. Zhang P, Wang F, Hu J, Sorrentino R. Towards personalized medicine: leveraging patient similarity and drug similarity analytics. AMIA Jt Summits Transl Sci Proc. 2014;2014:132–6.
38. Zhou X, Han H, Chankai I, Prestrud A, Brooks A. Approaches to Text Mining for Clinical Medical Records. In: New York, NY, USA: ACM; 2006. p. 235–239. doi:10.1145/1141277.1141330.
