# Supplementary material for: Graph-Representation of Patient Data: a Systematic Literature Review
Source: J Med Syst. 2020 Mar 12;44(4):86. doi: 10.1007/s10916-020-1538-4 (PMC7067737; doi:10.1007/s10916-020-1538-4)
Supplement: Supplementary file 7 — (PDF 1.03 mb) [file 10916_2020_1538_MOESM7_ESM.pdf]

Title: Graph-Representation of Patient Data
A Systematic Literature Review

Journal: Journal of Medical Systems

Authors:

Jens Schrodt, jens.schrodt@med.uni-heidelberg.de Universitätsklinikum Heidelberg Institut für Medizinische Biometrie und Informatik, Im Neuenheimer Feld 130.3, 69120 Heidelberg, Germany, orcid: 0000-0002-9768-4781

Aleksei Dudchenko, Aleksei.Dudchenko@med.uni-heidelberg.de Universitätsklinikum Heidelberg Institut für Medizinische Biometrie und Informatik, Im Neuenheimer Feld 130.3, 69120 Heidelberg, Germany

Petra Knaup, petra.knaup@med.uni-heidelberg.de Universitätsklinikum Heidelberg Institut für Medizinische Biometrie und Informatik, Im Neuenheimer Feld 130.3, 69120 Heidelberg, Germany

Matthias Ganzinger, matthias.ganzinger@med.uni-heidelberg.de Universitätsklinikum Heidelberg Institut für Medizinische Biometrie und Informatik, Im Neuenheimer Feld 130.3, 69120 Heidelberg, Germany

Suppl. Table 5: list of all investigated papers

Year	Title	Author, editor or organization	Ref.
1992	Abbs v. Sullivan		[1]
2014	Next generation phenotyping using the unified medical language system	Adamusiak, Tomasz; Shimoyama, Naoki; Shimoyama, Mary	[2]
2001	Diagnostic predictors of complications and survival after renal transplantation in cats	Adin, C. A.; Gregory, C. R.; Kyles, A. E.; Cowgill, L.	[3]
2014	Use of two-part regression calibration model to correct for measurement error in episodically consumed foods in a single-replicate study design: EPIC case study	Agogo, George O.; van der Voet, Hilko; van't Veer, Pieter; Ferrari, Pietro; Leenders, Max; Muller, David C.; Sanchez-Cantalejo, Emilio; Bamia, Christina; Braaten, Tonje; Knuppel, Sven; Johansson, Ingegerd; van Eeuwijk, Fred A.; Boshuizen, Hendriek	[4]
2017	Disease-Based Clustering of Hospital Admission: Disease Network of Hospital Networks Approach	Albarakati, N.; Obradovic, Z.	[5]
2005	A New Distance for High Level RNA Secondary Structure Comparison	Allali, Julien; Sagot, Marie-France	[6]
2011	Application of the Spreading Activation Technique for Recommending Concepts of Well-known Ontologies in Medical Systems	Alvarez, Jose Mar\'ıa; Polo, Luis; Jimenez, Weena; Abella, Pablo; Labra, Jose Emilio	[7]
2014	The origin of regional failure in oral cavity squamous cell carcinoma with pathologically negative neck metastases	Amit, Moran; Yen, Tzu Chen; Liao, Chun Ta; Chaturvedi, Pankaj; Agarwal, Jai Prakash; Kowalski, Luiz Paulo; Kohler, Hugo F.; Ebrahimi, Ardalan; Clark, Jonathan R.; Cernea, Claudio Roberto; Brandao, Jose S.; Kreppel, Matthias; Zoller, Joachim E.; Leider-Trejo, Leonor; Bachar, Gideon; Shpitzer, Thomas; Bolzoni, Andrea Villaret; Patel, Raj P.; Jonnalagadda, Sashikanth; Robbins, Thomas Kevin; Shah, Jatin P.; Patel, Snehal G.; Gil, Ziv	[8]

2013	An implementation of secure multi-party computations to preserve privacy when processing EMR data	Andersen, A.	[9]
2014	SNOOP: Privacy Preserving Middleware for Secure Multi-party Computations	Andersen, Anders	[10]
2014	Privacy preserving health data processing	Andersen, A.; Yigzaw, K. Y.; Karlsen, R.	[11]
2017	Adherence to guidelines and breast cancer patients survival: a population-based cohort study analyzed with a causal inference approach	Andreano, Anita; Rebora, Paola; Valsecchi, Maria Grazia; Russo, Antonio Giampiero	[12]
2017	Building a Dossier on the Cheap: Integrating Distributed Personal Data Resources Under Cost Constraints	Anindya, Imrul Chowdhury; Roy, Harichandan; Kantarcioglu, Murat; Malin, Bradley	[13]
2010	Integrating image and text information for biomedical information retrieval	Antani, S.	[14]
2016	Altered cross-frequency coupling in resting-state MEG after mild traumatic brain injury	Antonakakis, Marios; Dimitriadis, Stavros I.; Zervakis, Michalis; Micheloyannis, Sifis; Rezaie, Roozbeh; Babajani-Feremi, Abbas; Zouridakis, George; Papanicolaou, Andrew C.	[15]
2011	Data Mining of Audiology Patient Records: Factors Influencing the Choice of Hearing Aid Type	Anwar, Muhammad Naveed; Oakes, Michael Philip	[16]
2012	Data mining of audiology patient records: factors influencing the choice of hearing aid type	Anwar, Muhammad N.; Oakes, Michael P.	[17]
2005	Public health alert system for health networks: application to cardiology	Arantes, W. Manzi de; Verdier, C.	[18]
2015	Kidnew: The Kidney Transplant Patient Personal Health Buddy	Arcilla, Mary Jane B.; Ang, Viana Celina T.; Ochoa, Mikaela Nazarene; Padua, Christian Paulo L.; Payawal, Riezl E.	[19]
2016	Towards Longitudinal Analysis of a Population's Electronic Health Records Using Factor Graphs	Athreya, A. P.; Ngiam, K. Y.; Luo, Z.; Tai, E. S.; Kalbarczyk, Z.; Iyer, R. K.	[20]
2007	GRAFIP: A FRAMEWORK FOR THE REPRESENTATION OF HEALTHY AND PATHOLOGICAL CEREBRAL INFORMATION	Atif, J.; Hudelot, C.; Nempont, O.; Richard, N.; Batrancourt, B.; Angelini, E.; Bloch, I.	[21]
2015	Impact of the Mobile HealthPROMISE Platform on the Quality of Care and Quality of Life in Patients With Inflammatory Bowel Disease: Study Protocol of a Pragmatic Randomized Controlled Trial	Atreja, Ashish; Khan, Sameer; Rogers, Jason D.; Otobo, Emamuzo; Patel, Nishant P.; Ullman, Thomas; Colombel, Jean Fred; Moore, Shirley; Sands, Bruce E.	[22]
2014	A quality improvement study to improve inpatient problem list use	Bakel, Leigh Anne; Wilson, Karen; Tyler, Amy; Tham, Eric; Reese, Jennifer; Bothner, Joan; Kaplan, David W.	[23]
2007	Analgesic quality in a postoperative pain service: continuous assessment with the cumulative sum (cusum) method	Baptista Macaroff, W. M.; Castroman Espasandin, P.	[24]
2008	A Graph Approach to the Threshold All-against-all Substring Matching Problem	Barsky, Marina; Stege, Ulrike; Thomo, Alex; Upton, Chris	[25]
2010	Cancer survival as a function of age at diagnosis: a study of the Surveillance, Epidemiology and End Results database	Bassily, Mena N.; Wilson, Richard; Pompei, Francesco; Burmistrov, Dimitriy	[26]
2008	Representing health, disorder and their transitions by digraphs	Bassoe, Carl-Fredrik	[27]
1992	NATURAL-LANGUAGE PROCESSING AND SEMANTICAL REPRESENTATION OF MEDICAL TEXTS	Baud, R. H.; Am RASSINOUX; SCHERRER, [JR]	[28]
1995	REPRESENTING CLINICAL NARRATIVES USING CONCEPTUAL GRAPHS	Baud, R. H.; Am RASSINOUX; WAGNER, J. C.; Lovis, C.; JUGE, C.; ALPAY, L. L.; Michel, P. A.; Degoulet, P.; SCHERRER, [JR]	[29]
1992	Natural language processing and semantical representation of medical texts	Baud, R. H.; Rassinoux, A. M.; Scherrer, J. R.	[30]
2017	Knowledge graph prediction of unknown adverse drug reactions and validation in electronic health records	Bean, Daniel M.; Wu, Honghan; Iqbal, Ehtesham; Dzahini, Olubanke; Ibrahim, Zina M.; Broadbent, Matthew; Stewart, Robert; Dobson, Richard J. B.	[31]
2018	Author Correction: Knowledge graph prediction of unknown adverse drug reactions and validation in electronic health records	Bean, Daniel M.; Wu, Honghan; Iqbal, Ehtesham; Dzahini, Olubanke; Ibrahim, Zina M.; Broadbent, Matthew; Stewart, Robert; Dobson, Richard J. B.	[32]

1974	Computer-based hypertension clinic records: a co-operative study	Beilin, L. J.; Bulpitt, C. J.; Coles, E. C.; Dollery, C. T.; Johnson, B. F.; Mearns, C.; Munro-Faure, A. D.; Turner, S. C.	[33]
2004	Is time-slice analysis superior to total hospital length of stay in demonstrating the effectiveness of a month-long intensive effort on a medicine service?	Bellin, Eran; Kalkut, Gary	[34]
2014	Toward the design of a nursing ontology system	Benedik, Peter; Rajkovic, Uros; Sustersic, Olga	[35]
2007	Phylogenetic Networks Based on the Molecular Clock Hypothesis	Bereg, Sergey; Zhang, Yuanyi	[36]
1995	The compositional approach for representing medical concept systems	Bernauer, J.; Franz, M.; Schoop, D.; Schoop, M.; Pretschner, D. P.	[37]
2005	Topological Rearrangements and Local Search Method for Tandem Duplication Trees	Bertrand, Denis; Gascuel, Olivier	[38]
1997	Computerized patient records benefit physician offices	Bingham, A.	[39]
2016	Requirements for the formal representation of pathophysiology mechanisms by clinicians	Bono, B. de; Helvensteijn, M.; Kokash, N.; Martorelli, I.; Sarwar, D.; Islam, S.; Grenon, P.; Hunter, P.	[40]
2009	Consistency of Topological Moves Based on the Balanced Minimum Evolution Principle of Phylogenetic Inference	Bordewich, Magnus; Gascuel, Olivier; Huber, Katharina T.; Moulton, Vincent	[41]
2012	Tables or bar graphs? Presenting test results in electronic medical records	Brewer, Noel T.; Gilkey, Melissa B.; Lillie, Sarah E.; Hesse, Bradford W.; Sheridan, Stacey L.	[42]
2009	Usability testing finds problems for novice users of pediatric portals	Britto, Maria T.; Jimison, Holly B.; Munafo, Jennifer Knopf; Wissman, Jennifer; Rogers, Michelle L.; Hersh, William	[43]
2004	Constructing Chromosome Scale Suffix Trees	Brown, A. L.	[44]
2018	Applications of network analysis to routinely collected health care data: a systematic review	Brunson, Jason Cory; Laubenbacher, Reinhard C.	[45]
2004	Presentation of medical guidelines on a computer	Buchtela, David; Anger, Zdenek; Peleska, Jan; Vesely, Arnost; Zvarova, Jana	[46]
2002	Integrated visualization of problemcentric urologic patient records	Bui, Aleex A. T.; Taira, Ricky K.; Churchill, Bernard; Kangarloo, Hooshang	[47]
1986	Use of the labor graph in Malawi	Burgess, H. A.	[48]
1997	Practical use of computerized hospital information systems to improve blood transfusion	Butch, S. H.	[49]
1994	A LOGICAL FOUNDATION FOR REPRESENTATION OF CLINICAL-DATA	CAMPBELL, K. E.; DAS, A. K.; MUSEN, M. A.	[50]
2015	An alternative database approach for management of SNOMED CT and improved patient data queries	Campbell, W. Scott; Pedersen, Jay; McClay, James C.; Rao, Praveen; Bastola, Dhundy; Campbell, James R.	[51]
1982	Legal abortion: the public health record	Cates, W. [JR]	[52]
2009	Ranking Weblogs by Analyzing Reading and Commenting Activities	Cen, Songxiang; Han, Li; Ma, Jian	[53]
2018	Non-AIDS complexity amongst patients living with HIV in Sydney: risk factors and health outcomes	Chan, Derek J.; Furner, Virginia; Smith, Don E.; Dronavalli, Mithilesh; Bopage, Rohan I.; Post, Jeffrey J.; Bhardwaj, Anjali K.	[54]
2008	Technical Perspective: Finding a Good Neighbor, Near and Fast	Chazelle, Bernard	[55]
2016	Mining Health Examination Records #x2014;A Graph-Based Approach	Chen, L.; Li, X.; Sheng, Q. Z.; Peng, W. C.; Bennett, J.; Hu, H. Y.; Huang, N.	[56]
2015	Mining Symptom-Herb Patterns from Patient Records Using Tripartite Graph	Chen, Jinpeng; Poon, Josiah; Poon, Simon K.; Xu, Ling; Sze, Daniel M. Y.	[57]
2014	Patterns and trends with cancer incidence and mortality rates reported by the China National Cancer Registry	Chen, Peng-Lai; Zhao, Ting; Feng, Rui; Chai, Jing; Tong, Gui-Xian; Wang, De-Bin	[58]
2015	Predictive analytics on Electronic Health Records (EHRs) using Hadoop and Hive	Chennamsetty, H.; Chalasani, S.; Riley, D.	[59]
2017	GRAM: Graph-based Attention Model for Healthcare Representation Learning	Choi, Edward; Bahadori, Mohammad Taha; Le Song; Stewart, Walter F.; Sun, Jimeng	[60]
2013	A Transcript Perspective on Evolution	Christinat, Yann; Moret, Bernard M. E.	[61]

2014	Observation charts with overlapping blood pressure and heart rate graphs do not yield the performance advantage that health professionals assume: an experimental study	Christofidis, Melany J.; Hill, Andrew; Horswill, Mark S.; Watson, Marcus O.	[62]
2011	Post-treatment (1)(8)FFDG maximum standardized uptake value as a prognostic marker of recurrence in endometrial carcinoma	Chung, Hyun Hoon; Kim, Jae Weon; Kang, Keon Wook; Park, Noh-Hyun; Song, Yong-Sang; Chung, June-Key; Kang, Soon-Beom	[63]
1994	Knowledge-based approaches to the maintenance of a large controlled medical terminology	Cimino, J. J.; Clayton, P. D.; Hripcsak, G.; Johnson, S. B.	[64]
2011	Patterns and effects of missing comorbidity data for patients starting renal replacement therapy in England, Wales and Northern Ireland	Collier, Timothy; Steenkamp, Retha; Tomson, Charlie; Caskey, Fergus; Ansell, David; Roderick, Paul; Nitsch, Dorothea	[65]
2017	Using formal methods for content validation of medical procedure documents	Cota, Erika; Ribeiro, Leila; Bezerra, Jonas Santos; Costa, Andrei; da Silva, Rosiana Estefane; Cota, Glaucia	[66]
2012	Steganography and data hiding in flash video (FLV)	Cruz, J. P.; Libatique, N. J.; Tangonan, G.	[67]
2017	Visual Verification of Cancer Staging for Therapy Decision Support	Cypko, M. A.; Wojdziak, J.; Stoehr, M.; Kirchner, B.; Preim, B.; Dietz, A.; Lemke, H. U.; Oeltze-Jafra, S.	[68]
2015	Influence Diagram As a Support Tool for Clinical Decisions In Cardiopulmonary And Metabolic Rehabilitation	da Silva Klahr, Patricia; Correa Coronel, Christian; Cabral Robinson, Caroline; Fonseca, Joao Marcelo; Dias Flores, Cecilia; Della Mea Plentz, Rodrigo	[69]
2015	A grammar-based approach to model the patient's clinical trajectory after a mild traumatic brain injury	Dabek, F.; Caban, J. J.	[70]
2015	Visualization of Longitudinal Clinical Trajectories Using a Graph-based Approach	Dabek, Filip; Chen, Jian; Garbarino, Alexander; Caban, Jesus J.	[71]
2008	Personal data and confidentiality on the Internet	Dahl, Mads Ronald; Vedsted, Peter	[72]
2013	Observational data for comparative effectiveness research: An emulation of randomised trials of statins and primary prevention of coronary heart disease	Danaei, Goodarz; Garcia Rodriguez, Luis A.; Fernandez Cantero, Oscar; Logan, Roger; Hernan, Miguel A.	[73]
2017	Automated clinical diagnosis: The role of content in various sections of a clinical document	Datla, V.; Hasan, S. A.; Qadir, A.; Lee, K.; Ling, Y.; Liu, J.; Farri, O.	[74]
2011	Body mass index and weight-for-length ratio references for infants born at 33-42 weeks gestation: a new tool for anthropometric assessment	Davidson, Shmuel; Natan, Dafna; Novikov, Ilya; Sokolover, Nir; Erlich, Avi; Shamir, Raanan	[75]
2012	A Characterization of the Set of Species Trees That Produce Anomalous Ranked Gene Trees	Degnan, James H.; Rosenberg, Noah A.; Stadler, Tanja	[76]
1982	A problem-oriented approach to journal selection for hospital libraries	Delman, B. S.	[77]
2002	Development and evaluation of patient-centered software for a weight-management clinic	Dent, R. M.; Penwarden, R. M.; Harris, N.; Hotz, S. B.	[78]
2014	Graph-theoretic Analysis of Epileptic Seizures on Scalp EEG Recordings	Dhulekar, Nimit; Oztan, Basak; Yener, Bülent; Bingol, Haluk O.; Irim, Gulcin; Aktekin, Berrin; Aykut-Bingöl, Canan	[79]
2015	The transverse diameter of the chest on routine radiographs reliably estimates gestational age and weight in premature infants	Dietz, Kelly R.; Zhang, Lei; Seidel, Frank G.	[80]
1985	A microcomputer monitoring and data-acquisition system for intensive care units	Diprose, G. K.; Evans, D. H.; Levene, M. I.	[81]
2007	Design and evaluation of a temporal, graph-based language for querying collections of patient histories	Edsberg, Ole; Nordbo, Stein Jakob; Vinnes, Erik; Nytro, Oystein	[82]
2016	User acceptance of observation and response charts with a track and trigger system: a multisite staff survey	Elliott, Doug; Allen, Emily; McKinley, Sharon; Perry, Lin; Duffield, Christine; Fry, Margaret; Gallagher, Robyn; Iedema, Rick; Roche, Michael	[83]
2010	NORDCAN--a Nordic tool for cancer information, planning, quality control and research	Engholm, Gerda; Ferlay, Jacques; Christensen, Niels; Bray, Freddie; Gjerstorff, Marianne L.; Klint, Asa; Kotlum, Joanis E.; Olafsdottir, Elinborg; Pukkala, Eero; Storm, Hans H.	[84]

2008	Animal-related injuries: a population-based study of a five-state region in the upper Midwest: Regional Rural Injury Study II	Erkal, Sibel; Gerberich, Susan Goodwin; Ryan, Andrew D.; Renier, Colleen M.; Alexander, Bruce H.	[85]
2015	Predicting Sequences of Clinical Events by Using a Personalized Temporal Latent Embedding Model	Esteban, C.; Schmidt, D.; Krompaß, D.; Tresp, V.	[86]
2017	Road traffic noise and registry based use of sleep medication	Evandt, Jorunn; Oftedal, Bente; Krog, Norun Hjertager; Skurtveit, Svetlana; Nafstad, Per; Schwarze, Per E.; Skovlund, Eva; Houthuijs, Danny; Aasvang, Gunn Marit	[87]
1997	Misclassification and selection bias in case-control studies using an automated database	Evans, J. M.; MacDonald, T. M.	[88]
2005	Graphical Rule-based Representation of Signal-transduction Networks	Faeder, James R.; Blinov, Michael L.; Hlavacek, William S.	[89]
2002	Future Directions in Data Mining: Streams, Networks, Self-similarity and Power Laws	Faloutsos, Christos	[90]
1994	Beyond Uniformity and Independence: Analysis of R-trees Using the Concept of Fractal Dimension	Faloutsos, Christos; Kamel, Ibrahim	[91]
2015	Hierarchical Graph-Coupled HMMs for Heterogeneous Personalized Health Data	Fan, Kai; Eisenberg, Marisa; Walsh, Alison; Aiello, Allison; Heller, Katherine	[92]
2008	Ontology-Aware Search on XML-based Electronic Medical Records	Farfan, F.; Hristidis, V.; Ranganathan, A.; Burke, R. P.	[93]
2014	Novel data sources for women's health research: mapping breast screening online information seeking through Google trends	Fazeli Dehkordy, Soudabeh; Carlos, Ruth C.; Hall, Kelli S.; Dalton, Vanessa K.	[94]
2007	Faster Algorithms for Sorting by Transpositions and Sorting by Block Interchanges	Feng, Jianxing; Zhu, Daming	[95]
2009	Nursing audit: The impact of nursing annotation in the context of hospital gloss	Ferreira, Tania S.; Luiz de Souza-Braga, Andre; Soraia Cavalcanti-Valente, Geilsa; Ferreira de Souza, Deise; Moreira Carvalho-Alves, Enilda	[96]
2014	Building the graph of medicine from millions of clinical narratives	Finlayson, Samuel G.; LePendu, Paea; Shah, Nigam H.	[97]
2011	An efficient record linkage scheme using graphical analysis for identifier error detection	Finney, John M.; Walker, A. Sarah; Peto, Tim E. A.; Wyllie, David H.	[98]
1995	Seven years experience with a computerized diabetes clinic database	Flack, J. R.	[99]
1995	Context trees: representing co-operative healthcare activities in IREP	Florit, A. M.; Mori, A. R.; Simone, M. de; d'Annunzio, V.; Ricci, F. L.; Lalle, C.	[100]
2018	Multimedia-enhanced Radiology Reports: Concept, Components, and Challenges	Folio, Les R.; Machado, Laura B.; Dwyer, Andrew J.	[101]
2007	Progress report on a post-radial keratotomy patient 20 years after surgery	Forister, Julie F. Y.; Sun, Amy; Weissman, Barry A.	[102]
2001	Computer-aided risk management--a software tool for the Hidep model	Fors, U. G.; Sandberg, H. C.	[103]
2018	Presentation of laboratory test results in patient portals: influence of interface design on risk interpretation and visual search behaviour	Fraccaro, Paolo; Vigo, Markel; Balatsoukas, Panagiotis; van der Veer, Sabine N.; Hassan, Lamiece; Williams, Richard; Wood, Grahame; Sinha, Smeeta; Buchan, Iain; Peek, Niels	[104]
1993	An Incremental Algorithm for Building Temporal Quadtrees	Frazier, George F.	[105]
2013	A mHealth application for chronic wound care: findings of a user trial	Friesen, Marcia R.; Hamel, Carole; McLeod, Robert D.	[106]
2007	Projecting individualized absolute invasive breast cancer risk in African American women	Gail, Mitchell H.; Costantino, Joseph P.; Pee, David; Bondy, Melissa; Newman, Lisa; Selvan, Mano; Anderson, Garnet L.; Malone, Kathleen E.; Marchbanks, Polly A.; McCaskill-Stevens, Worta; Norman, Sandra A.; Simon, Michael S.; Spirtas, Robert; Ursin, Giske; Bernstein, Leslie	[107]
2014	Using an ontological modeling to evaluate the consistency of clinical practice guidelines: application to the comparison of three guidelines on the management of adult hypertension	Galopin, Alexandre; Bouaud, Jacques; Pereira, Suzanne; Seroussi, Brigitte	[108]
2015	An Ontology-Based Clinical Decision Support System for the Management of Patients with Multiple Chronic Disorders	Galopin, Alexandre; Bouaud, Jacques; Pereira, Suzanne; Seroussi, Brigitte	[109]

2013	Semi-supervised clinical text classification with Laplacian SVMs: an application to cancer case management	Garla, Vijay; Taylor, Caroline; Brandt, Cynthia	[110]
1996	Real-time Incremental Visualization of Dynamic Ultrasound Volumes Using Parallel BSP Trees	Garrett, William F.; Fuchs, Henry; Whitton, Mary C.; State, Andrei	[111]
2014	Technical report: an ePRO patient reported outcome program for the evaluation of patients with irritable bowel syndrome	Gerson, C. D.; Gerson, M-J	[112]
1974	Interactive Analysis and Display of the Electroencephalogram (EEG) in Real Time	Gevins, A. S.; Yeager, C. L.; Diamond, S. L.	[113]
2016	A Fast Sketch-based Assembler for Genomes	Ghosh, Priyanka; Kalyanaraman, Ananth	[114]
2007	Emotional and cognitive information processing in web-based medical education	Giani, U.; Brascio, G.; Bruzzese, D.; Garzillo, C.; Vigilante, S.	[115]
2013	Evaluating Theoretical Models of Protein Interaction Network Evolution Without Seed Graphs	Gibson, Todd A.; Goldberg, Debra S.	[116]
2011	Web-Based Support for Fracture Healing Evaluation and Monitoring	Glinkowski, Wojciech	[117]
2016	An automated knowledge-based textual summarization system for longitudinal, multivariate clinical data	Goldstein, Ayelet; Shahar, Yuval	[118]
2013	Automatic Generation of a Qualified Medical Knowledge Graph and Its Usage for Retrieving Patient Cohorts from Electronic Medical Records	Goodwin, T.; Harabagiu, S. M.	[119]
2016	Embedding Open-domain Common-sense Knowledge from Text	Goodwin, Travis; Harabagiu, Sanda	[120]
2016	Medical Question Answering for Clinical Decision Support	Goodwin, Travis R.; Harabagiu, Sanda M.	[121]
2018	Knowledge Representations and Inference Techniques for Medical Question Answering	Goodwin, Travis R.; Harabagiu, Sanda M.	[122]
2015	Stabilizing high-dimensional prediction models using feature graphs	Gopakumar, Shivapratap; Tran, Truyen; Nguyen, Tu Dinh; Phung, Dinh; Venkatesh, Svetha	[123]
2008	Tracing and cataloguing knowledge in an e-health cardiology environment	Gortzis, L. G.; Nikiforidis, G.	[124]
2013	Potential value of health information exchange for people with epilepsy: crossover patterns and missing clinical data	Grinspan, Zachary M.; Abramson, Erika L.; Banerjee, Samprit; Kern, Lisa M.; Kaushal, Rainu; Shapiro, Jason S.	[125]
2014	Detecting Roles and Anomalies in Hospital Access Audit Logs	Gunter, Carl	[126]
2012	Towards the Assessment of Semantic Similarity Analysis of Protein Data: Main Approaches and Issues	Guzzi, Pietro Hiram; Mina, Marco	[127]
2004	Management of pregnancies complicated by anti-c isoimmunization	Hackney, David N.; Knudtson, Eric J.; Rossi, Karen Q.; Krugh, Dave; O'Shaughnessy, Richard W.	[128]
2005	Exact Match Search in Sequence Data Using Suffix Trees	Halachev, Mihail; Shiri, Nematollaah; Thamildurai, Anand	[129]
2004	Universal electronic health record MUDR	Hanzlicek, Petr; Spidlen, Josef; Nagy, Miroslav	[130]
2005	User interface of MUDR electronic health record	Hanzlicek, Petr; Spidlen, Josef; Heroutova, Helena; Nagy, Miroslav	[131]
2015	Big mechanisms for processing big data in medical informatics	Harabagiu, S.	[132]
2013	Next Generation Linkage Management System	Harris, Julie	[133]
2011	Low Cost Digital Stethoscope for Heart Sounds	Harsola, A.; Thale, S.; Panse, M. S.	[134]
2016	Design and feasibility of integrating personalized PRO dashboards into prostate cancer care	Hartzler, Andrea L.; Izard, Jason P.; Dalkin, Bruce L.; Mikles, Sean P.; Gore, John L.	[135]
1982	Acquiring otoneurological results by computer. Evaluation after 2 years of experience	Hausler, R.; Washburn, D.; Rey, P.; Stefanoff, F.	[136]
2014	Orion: A system for modeling, transformation and visualization of multidimensional heterogeneous networks	Heer, Jeffrey; Perer, Adam	[137]

1997	Nursing classification systems: necessary but not sufficient for representing "what nurses do" for inclusion in computer-based patient record systems	Henry, S. B.; Mead, C. N.	[138]
1996	Constructing a Tree from Homeomorphic Subtrees, with Applications to Computational Evolutionary Biology	Henzinger, Monika Rauch; King, Valerie; Warnow, Tandy	[139]
2009	Wireless Applications for Hospital Epidemiology	Herman, Ted; Pemmaraju, Sriram V.; Segre, Alberto M.; Polgreen, Philip M.; Curtis, Donald E.; Fries, Jason; Hlady, Chris; Severson, Monica	[140]
2012	Graph-based signal integration for high-throughput phenotyping	Herskovic, Jorge R.; Subramanian, Devika; Cohen, Trevor; Bozzo-Silva, Pamela A.; Bearden, Charles F.; Bernstam, Elmer V.	[141]
2012	Workflow Forever: Semantic Web Semantic Models and Tools for Preserving and Digitally Publishing Computational Experiments	Hettne, Kristina; Soiland-Reyes, Stian; Klyne, Graham; Belhajjame, Khalid; Gamble, Matthew; Bechhofer, Sean; Roos, Marco; Corcho, Oscar	[142]
2008	The Austrian Stroke-Unit-Registry	Hofer, Christine; Kiechl, Stefan; Lang, Wilfried	[143]
2006	Surgical repair of coarctation of the aorta: up to 40 years of follow-up	Hoimyr, Hilde; Christensen, Thomas D.; Emmertsen, Kristian; Johnsen, Soren P.; Riis, Anders; Hansen, Ole Kromann; Hjortdal, Vibeke E.	[144]
2015	The Symptoms and Pathogenesis Entity Recognition of TCM Medical Records Based on CRF	Honglan, L.; Xiaona, Q.; Bin, F.	[145]
2009	Syndromic surveillance using ambulatory electronic health records	Hripcsak, George; Soulakis, Nicholas D.; Li, Li; Morrison, Frances P.; Lai, Albert M.; Friedman, Carol; Calman, Neil S.; Mostashari, Farzad	[146]
2017	Private and Secured Medical Data Transmission and Analysis for Wireless Sensing Healthcare System	Huang, H.; Gong, T.; Ye, N.; Wang, R.; Dou, Y.	[147]
2017	Evaluation of the “Angelina Jolie Effect” on Screening Mammography Utilization in an Academic Center	Huesch, Marco D.; Schetter, Susann; Segel, Joel; Chetlen, Alison	[148]
2001	Meta-modelling: the appropriate solution for a family of applications	Huet, B.; Artigou, J. Y.; Poirier, J.; Blain, G.	[149]
1982	An automaton computer program for a microcomputerized real-time (thesaurus based) abstract medical record	Huet, B.; Pourriat, J. L.; Martin, J.; Cupa, M.	[150]
2015	Security Analysis and Improvement of ‘a More Secure Anonymous User Authentication Scheme for the Integrated EPR Information System’	Islam, S. K. Hafizul; Khan, Muhammad Khurram; Li, Xiong	[151]
2013	Italian cancer figures, report 2012: Cancer in children and adolescents		[152]
1987	An Object-oriented Programming Discipline for Standard Pascal	Jacky, Jonathan P.; Kalet, Ira J.	[153]
2013	Significance of copper level in serum and routine laboratory parameters in estimation of outspreading of Hodgkin's lymphoma	Jamakovic, Mesud; Baljic, Rusmir	[154]
2001	Towards a Visual Query Interface for Phylogenetic Databases	Jamil, Hasan M.; Modica, Giovanni A.; Teran, Maria A.	[155]
2017	A Pilot Comparison of a Smartphone App With or Without 2-Way Messaging Among Chronic Pain Patients: Who Benefits From a Pain App?	Jamison, Robert N.; Jurcik, Dylan C.; Edwards, Robert R.; Huang, Chuan-Chin; Ross, Edgar L.	[156]
1986	Testing an expert system for hypertension	Jeunemaitre, X.; Degoulet, P.; Morice, V.; Chatellier, G.; Devries, C.; Plouin, P. F.; Boisvieux, J. F.; Menard, J.	[157]
2016	Predicting Comorbid Conditions and Trajectories using Social Health Records	Ji, Xiang; Ae Chun, Soon; Geller, James	[158]
2017	Learning and inference in knowledge-based probabilistic model for medical diagnosis	Jiang, Jingchi; Li, Xueli; Zhao, Chao; Guan, Yi; Yu, Qiubin	[159]
2014	A complementary graphical method for reducing and analyzing large data sets. Case studies demonstrating thresholds setting and selection	Jing, X.; Cimino, J. J.	[160]

2014	Identity Tracking in Big Data: Preliminary Research Using In-Memory Data Graph Models for Record Linkage and Probabilistic Signature Hashing for Approximate String Matching in Big Health and Human Services Databases	Jupin, Joseph; Shi, Justin Y.	[161]
2016	Stable clinical prediction using graph support vector machines	Kamkar, I.; Gupta, S.; Li, Cheng; Phung, D.; Venkatesh, S.	[162]
2015	Managing Data in Healthcare Information Systems: Many Models, One Solution	Kaur, K.; Rani, R.	[163]
2013	Unsupervised Extraction of Diagnosis Codes from EMRs Using Knowledge-Based and Extractive Text Summarization Techniques	Kavuluru, Ramakanth; Han, Sifei; Harris, Daniel	[164]
1992	Management of personal health examination data for a population by use of a portable computer	Kawada, T.; Aoki, S.; Suzuki, S.	[165]
2012	Lullaby: A Capture & Access System for Understanding the Sleep Environment	Kay, Matthew; Choe, Eun Kyoung; Shepherd, Jesse; Greenstein, Benjamin; Watson, Nathaniel; Consolvo, Sunny; Kientz, Julie A.	[166]
2007	Reduction of multi-dimensional laboratory data to a two-dimensional plot: a novel technique for the identification of laboratory error	Kazmierczak, Steven C.; Leen, Todd K.; Erdogmus, Deniz; Carreira-Perpinan, Miguel A.	[167]
2017	Toward Meaningful Care Plan Clinical Decision Support: Feasibility and Effects of a Simulated Pilot Study	Keenan, Gail M.; Lopez, Karen Dunn; Yao, Yingwei; Sousa, Vanessa E. C.; Stifter, Janet; Febretti, Alessandro; Johnson, Andrew; Wilkie, Diana J.	[168]
1984	Antenatal records: do they help us? A new record for watching fetal growth	Kennedy, I.; Ritter, H.	[169]
1979	A novel antenatal record to help midwives	Kennedy, I.; Stephens, B.	[170]
2012	The effect of complete integration of HIV and TB services on time to initiation of antiretroviral therapy: a before-after study	Kerschberger, Bernhard; Hilderbrand, Katherine; Boulle, Andrew M.; Coetzee, David; Goemaere, Eric; Azevedo, Virginia de; van Cutsem, Gilles	[171]
2013	Personalized Electronic Health Record System for Monitoring Patients with Chronic Disease	Khan, I. A.	[172]
2017	Summarizing Static and Dynamic Big Graphs	Khan, Arijit; Bhowmick, Sourav S.; Bonchi, Francesco	[173]
2014	Systems for Big-graphs	Khan, Arijit; Elnikety, Sameh	[174]
2013	Health care transformation through collaboration on open-source informatics projects: integrating a medical applications platform, research data repository, and patient summarization	Klann, Jeffrey G.; McCoy, Allison B.; Wright, Adam; Wattanasin, Nich; Sittig, Dean F.; Murphy, Shawn N.	[175]
2014	Decision support from local data: creating adaptive order menus from past clinician behavior	Klann, Jeffrey G.; Szolovits, Peter; Downs, Stephen M.; Schadow, Gunther	[176]
2013	Monitoring of dairy cow claw health status in 15 herds using the computerised documentation program Claw Manager and digital parameters	Kofler, J.; Pesenhofer, R.; Landl, G.; Sommerfeld-Stur, I.; Peham, C.	[177]
2014	Semantic Search As Inference: Applications in Health Informatics	Koopman, Bevan	[178]
2012	Graph-based Concept Weighting for Medical Information Retrieval	Koopman, Bevan; Zuccon, Guido; Bruza, Peter; Sitbon, Laurianne; Lawley, Michael	[179]
2011	Retrospective monitoring in the management of persistent asthma	Kourtis, Georgios; Caiaffa, Maria-Filomena; Forte, Cesarea; Scarlato, Marisa I.; Macchia, Luigi	[180]
2016	Using Arden Syntax for the Generation of Intelligent Intensive Care Discharge Letters	Kraus, Stefan; Castellanos, Ixchel; Albermann, Matthias; Schuettler, Christina; Prokosch, Hans-Ulrich; Staudigel, Martin; Toddenroth, Dennis	[181]
2008	Combining Two Forms of Simulation to Predict the Potential Impact of Interface Design on Technology-induced Error in Healthcare	Kushniruk, Andre; Borycki, Elizabeth; Anderson, James G.; Anderson, Marilyn M.	[182]
2014	Lifelog agent for human activity pattern analysis on health avatar platform	Kwon, Yongjin; Kang, Kyuchang; Bae, Changseok; Chung, Hee-Joon; Kim, Ju Han	[183]
2014	Merging Partially Labelled Trees: Hardness and a Declarative Programming Solution	Labarre, Anthony; Verwer, Sicco	[184]
2001	101 Optimal PDB Structure Alignments: A Branch-and-cut Algorithm for the Maximum Contact Map Overlap Problem	Lancia, Giuseppe; Carr, Robert; Walenz, Brian; Istrail, Sorin	[185]

2015	Provider-Driven Development of a Measurement Feedback System to Enhance Measurement-Based Care in VA Mental Health	Landes, Sara J.; Carlson, Eve B.; Ruzek, Josef I.; Wang, Dan; Hugo, Emily; DeGaetano, Noah; Chambers, Justin G.; Lindley, Steven E.	[186]
2015	Learning Curve Analysis and Surgical Outcomes of Single-port Laparoscopic Myomectomy	Lee, Hee Jun; Kim, Ju Yeong; Kim, Seul Ki; Lee, Jung Ryeol; Suh, Chang Suk; Kim, Seok Hyun	[187]
2008	Incidence of thyroid cancer in residents surrounding the Three Mile Island nuclear facility	Levin, Roger J.	[188]
2012	Associating clinical archetypes through UMLS Metathesaurus term clusters	Lezcano, Leonardo; Sanchez-Alonso, Salvador; Sicilia, Miguel-Angel	[189]
2017	Development of a web-based epidemiological surveillance system with health system response for improving maternal and newborn health: Field-testing in Thailand	Liabsuetrakul, Tippawan; Prappre, Tagoon; Pairot, Pakamas; Oumudee, Nurlisa; Islam, Monir	[190]
2011	Visual Exploration Across Biomedical Databases	Lieberman, Michael D.; Taheri, Sima; Guo, whatever; Mirrashed, Fatemeh; Yahav, Inbal; Aris, Aleks; Shneiderman, Ben	[191]
2017	Usability of data integration and visualization software for multidisciplinary pediatric intensive care: a human factors approach to assessing technology	Lin, Ying Ling; Guerguerian, Anne-Marie; Tomasi, Jessica; Laussen, Peter; Trbovich, Patricia	[192]
2014	Mockup design of personal health diary app for patients with chronic kidney disease	Lin, Hsiu-Wen; Wang, Yu-Jen; Jing, Ling-Fang; Chang, Polun	[193]
2015	A genetic algorithm enabled ensemble for unsupervised medical term extraction from clinical letters	Liu, Wei; Chung, Bo Chuen; Wang, Rui; Ng, Jonathon; Morlet, Nigel	[194]
2013	Mining Biomedical Ontologies and Data Using RDF Hypergraphs	Liu, H.; Dou, D.; Jin, R.; Lependu, P.; Shah, N.	[195]
2011	Protein Subcellular Localization Prediction with Associative Classification and Multi-class SVM	Liu, Yifeng; Guo, Zhaochen; Ke, Xiaodi; Za\"ıane, Osmar R.	[196]
2015	Temporal Phenotyping from Longitudinal Electronic Health Records: A Graph Based Framework	Liu, Chuanren; Wang, Fei; Hu, Jianying; Xiong, Hui	[197]
2000	Integration of computer-assembled digital images and text data as evidence for the oncological record	Liu, J. M.; Wu, H. W.; Chen, W. S.; Lin, W. C.; Chao, Y.; Lui, W. Y.; Whang-Peng, J.	[198]
2016	TimeSpan: Using Visualization to Explore Temporal Multi-dimensional Data of Stroke Patients	Loorak, Mona Hosseinkhani; Perin, Charles; Kamal, Noreen; Hill, Michael; Carpendale, Sheelagh	[199]
2016	Nurses' Numeracy and Graphical Literacy: Informing Studies of Clinical Decision Support Interfaces	Lopez, Karen Dunn; Wilkie, Diana J.; Yao, Yingwei; Sousa, Vanessa; Febretti, Alessandro; Stifter, Janet; Johnson, Andrew; Keenan, Gail M.	[200]
2001	Paragraph-oriented structure for narratives in medical documentation	Lovis, C.; Baud, R. H.; Revillard, C.; Pult, L.; Borst, F.; Geissbuhler, A.	[201]
2015	Subgraph augmented non-negative tensor factorization (SANTF) for modeling clinical narrative text	Luo, Yuan; Xin, Yu; Hochberg, Ephraim; Joshi, Rohit; Uzuner, Ozlem; Szolovits, Peter	[202]
2016	Deconstructing the smoking-preeclampsia paradox through a counterfactual framework	Luque-Fernandez, Miguel Angel; Zoega, Helga; Valdimarsdottir, Unnur; Williams, Michelle A.	[203]
2009	Classification of Patient Case Discussions Through Analysis of Vocalisation Graphs	Luz, Saturnino; Kane, Bridget	[204]
2016	A representational analysis of a temporal indeterminancy display in clinical events	Madkour, M.; Song, Hsing-yi; Du Jingcheng; Tao, C.	[205]
2014	Formal representation of service interactions for SaaS based applications	Mandal, A. K.; Sarkar, A.	[206]
2010	Defining quality-measurable medical alerts from incomplete data through fuzzy linguistic variables and modifiers	Manzi de Arantes, Wilmondes Jr; Verdier, Christine	[207]
2017	Intelligent MONitoring System for antiviral pharmacotherapy in patients with chronic hepatitis C (SiMON-VC)	Margusino-Framinan, Luis; Cid-Silva, Purificacion; Mena-de-Cea, Alvaro; Sanclaudio-Luhia, Ana Isabel; Castro-Castro, Jose Antonio; Vazquez-Gonzalez, Guillermo; Martin-Herranz, Isabel	[208]
2016	A Knowledge Map for Hospital Performance Concept: Extraction and Analysis: A Narrative Review Article	Markazi-Moghaddam, Nader; Arab, Mohammad; Ravaghi, Hamid; Rashidian, Arash; Khatibi, Toktam; Zargar Balaye Jame, Sanaz	[209]

2002	Relative bias in diet history measurements: a quality control technique for dietary intervention trials	Martin, Gina S.; Tapsell, Linda C.; Batterham, Marijka J.; Russell, Kenneth G.	[210]
2014	Improving search over Electronic Health Records using UMLS-based query expansion through random walks	Martinez, David; Otegi, Arantxa; Soroa, Aitor; Agirre, Eneko	[211]
2017	Validating EHR clinical models using ontology patterns	Martinez-Costa, Catalina; Schulz, Stefan	[212]
2015	Heterogeneous Compression of Large Collections of Evolutionary Trees	Matthews, Suzanne J.	[213]
2013	The Child Therapy Tracking System (CTTS): A model for an expressive therapy electronic health record (EHR)	Mattson, Donald C.; Yang, Jing	[214]
2009	Extracting Temporal Rules from Medical Data	Meamarzadeh, H.; Khayyambashi, M. R.; Saraee, M. H.	[215]
1997	PRIME-GC. A medical information retrieval prototype on the Web	Mechkour, M.; Mulhem, P.; Fourel, F.; Berrut, E. F. C.	[216]
2002	Analyzing the Semantics of Patient Data to Rank Records of Literature Retrieval	Mendonça, Eneida A.; Johnson, Stephen B.; Seol, Yoon-Ho; Cimino, James J.	[217]
2012	Common data model for natural language processing based on two existing standard information models: CDA+GrAF	Meystre, Stephane M.; Lee, Sanghoon; Jung, Chai Young; Chevrier, Raphael D.	[218]
2016	High-performance Data Structures for De Novo Assembly of Genomes: Cache Oblivious Generic Programming	Milicchio, Franco; Tradigo, Giuseppe; Veltri, Pierangelo; Prosperi, Mattia	[219]
2002	Scientific evaluation and quality assurance in refractive surgical interventions. Evaluation of the Datagraph med computer program	Mirshahi, A.; Kohnen, T.	[220]
2012	Cerebrovascular disease in 48 countries: secular trends in mortality 1950-2005	Mirzaei, Masoud; Truswell, A. Stewart; Arnett, Kathryn; Page, Andrew; Taylor, Richard; Leeder, Stephen R.	[221]
2016	Mobile-assisted remote healthcare delivery	Mondal, S.; Mukherjee, N.	[222]
2010	Discovering client and intervention patterns in home visiting data	Monsen, Karen A.; Banerjee, Arindam; Das, Puja	[223]
2005	A rapid self-administered food frequency questionnaire for the evaluation of dietary protein intake	Morin, Patricia; Herrmann, Francois; Ammann, Patrick; Uebelhart, Brigitte; Rizzoli, Rene	[224]
2014	The new culture of dematerialized health	Moruzzi, Mauro	[225]
2009	Shrinkage Effect in Ancestral Maximum Likelihood	Mossel, Elchanan; Roch, Sebastien; Steel, Mike	[226]
2011	A Scalable Multi-scale Framework for Parallel Simulation and Visualization of Microbial Evolution	Mozhayskiy, Vadim; Miller, Bob; Ma, Kwan-Liu; Tagkopoulos, Ilias	[227]
1997	The CliniCon framework for context representation in electronic patient records	Muller, R.	[228]
1997	The CliniCon framework for context representation in electronic patient records	Muller, R.	[229]
1997	THEMPO: a knowledge-based system for therapy planning in pediatric oncology	Muller, R.; Sergl, M.; Nauerth, U.; Schoppe, D.; Pommerening, K.; Dittrich, H. M.	[230]
1996	A graph-grammar approach to represent causal, temporal and other contexts in an oncological patient record	Muller, R.; Thews, O.; Rohrbach, C.; Sergl, M.; Pommerening, K.	[231]
2005	Electronic growth charts: watching our patients grow	Murphy, Cynthia A.; Carstens, Kimberly; Villamayor, Precy	[232]
2014	Assessment of paediatric inpatient care during a multifaceted quality improvement intervention in Kenyan district hospitals--use of prospectively collected case record data	Mwaniki, Paul; Ayieko, Philip; Todd, Jim; English, Mike	[233]
2012	Early vaccinations are not risk factors for celiac disease	Myleus, Anna; Stenlund, Hans; Hernell, Olle; Gothefors, Leif; Hammarstrom, Marie-Louise; Persson, Lars-Ake; Ivarsson, Anneli	[234]
2012	Computing Gene Functional Similarity Using Combined Graphs	Nagar, Anurag; Al-Mubaid, Hisham; Bettayeb, Said	[235]
1998	Clinical efficiency tools improve stroke management in a rural southern health system	Newell, S. D. [JR]; Englert, J.; Box-Taylor, A.; Davis, K. M.; Koch, K. E.	[236]

2014	PARAMO: a PARAllel predictive MOdeling platform for healthcare analytic research using electronic health records	Ng, Kenney; Ghoting, Amol; Steinhubl, Steven R.; Stewart, Walter F.; Malin, Bradley; Sun, Jimeng	[237]
2017	Automated Medical Diagnosis by Ranking Clusters Across the Symptom-Disease Network	Ni, J.; Fei, H.; Fan, W.; Zhang, X.	[238]
2010	A Fielded Wiki for Personality Genetics	Nielsen, Finn \AArup	[239]
2013	Towards generating a patient's timeline: extracting temporal relationships from clinical notes	Nikfarjam, Azadeh; Emadzadeh, Ehsan; Gonzalez, Graciela	[240]
2008	Temporal Pattern Discovery for Trends and Transient Effects: Its Application to Patient Records	Norén, G. Niklas; Bate, Andrew; Hopstadius, Johan; Star, Kristina; Edwards, I. Ralph	[241]
2010	Temporal pattern discovery in longitudinal electronic patient records	Noren, G. Niklas; Hopstadius, Johan; Bate, Andrew; Star, Kristina; Edwards, I. Ralph	[242]
2015	Simultaneous Modeling of Multiple Diseases for Mortality Prediction in Acute Hospital Care	Nori, Nozomi; Kashima, Hisashi; Yamashita, Kazuto; Ikai, Hiroshi; Imanaka, Yuichi	[243]
1988	Cancer registration using case history database in hospital information system	Nose, Y.; Akazawa, K.; Watanabe, Y.; Yokota, M.; Okamura, S.; Maehara, Y.; Sugimachi, K.	[244]
1998	Development and evaluation of regional health database systems	Ogushi, Y.; Haruki, Y.; Okada, Y.; Takahashi, M.; Shimizu, M.; Izumi, Y.; Watabe, T.; Kobayashi, S.; Okuyama, J.; Kurita, Y.	[245]
2012	A hypothesis-generating support system using medical records for clinical knowledge acquisition	Okamoto, K.; Tanaka, H.; Takemura, T.; Kume, N.; Kuroda, T.; Yoshihara, H.	[246]
2011	Managing Security and Privacy in Ubiquitous eHealth Information Interchange	Oladimeji, Ebenezer A.; Chung, Lawrence; Jung, Hyo Taeg; Kim, Jaehyoun	[247]
1995	Read Codes Version 3: a user led terminology	O'Neil, M.; Payne, C.; Read, J.	[248]
2016	Generation of Sentence Template Graph from SOAP Format Medical Documents	Onimura, N.; Yamashita, T.; Nakayama, N.; Soejima, H.; Hirokawa, S.	[249]
2012	Using a time-geographical diary method in order to facilitate reflections on changes in patterns of daily occupations	Orban, Kristina; Edberg, Anna-Karin; Erlandsson, Lena-Karin	[250]
2014	Effective methods of organizing complex information in advanced cancer patients	Oshiro, Tatsuo; Oshiro, Hisako; Tanimizu, Masahito	[251]
2015	The Impact of Comorbid Depression on Educational Inequality in Survival after Acute Coronary Syndrome in a Cohort of 83 062 Patients and a Matched Reference Population	Osler, Merete; Prescott, Eva; Wium-Andersen, Ida Kim; Ibfelt, Else Helene; Jorgensen, Martin Balslev; Andersen, Per Kragh; Jorgensen, Terese Sara Hoj; Wium-Andersen, Marie Kim; Martensson, Solvej	[252]
2016	Electronic health records: Improvement to healthcare decision-making	Osop, H.; Sahama, T.	[253]
2008	International variation in the incidence of oral and pharyngeal cancer	O'Sullivan, E. M.	[254]
2013	Mediation and graph data models for medical data integration	Pabón, M. C.; Montoya, G. A.; Millán, M.	[255]
2010	Fifty years of cancer incidence: CI5 I-IX	Parkin, D. Max; Ferlay, Jacques; Curado, Maria-Paula; Bray, Freddie; Edwards, Brenda; Shin, Hai-Rim; Forman, David	[256]
2013	Mining drug-drug interaction patterns from linked data: A case study for Warfarin, Clopidogrel, and Simvastatin	Pathak, J.; Kiefer, R. C.; Chute, C. G.	[257]
2016	Digital signal preservation approaches of archived biomedical paper records #x2014; A review	Patil, R.; Karandikar, R. G.	[258]
2006	Fall-related injuries among agricultural household members: Regional Rural Injury Study II (RRIS-II)	Paulson, Erin H.; Gerberich, Susan Goodwin; Alexander, Bruce H.; Ryan, Andrew; Renier, Colleen M.; Zhang, Xueying; French, L. Ronald; Masten, Ann S.; Carlson, Kathleen Ferguson	[259]
2012	Development of clinical pharmacy productivity metrics	Pawloski, Pamala; Cusick, Dona; Amborn, Lori	[260]
1999	Graphing clinical events in the VA Computerized Patient Record System	Payne, T. H.; Andrews, R. D.; Breeling, J.; Ben Davoren, J.; Smith, R. M.; Volpp, B.	[261]
2017	Social Network Analytics, Data Science Ethics & Privacy-preserving Analytics	Pedreschi, Dino	[262]

1992	Physicians take to the field	Penfield, W.	[263]
2011	Actual state and perspectives of e-health in Austria and international--an overview	Pfeiffer, Karl P.	[264]
2015	Utilizing the Wikidata System to Improve the Quality of Medical Content in Wikipedia in Diverse Languages: A Pilot Study	Pfundner, Alexander; Schnoeberg, Tobias; Horn, John; Boyce, Richard D.; Samwald, Matthias	[265]
1995	Free text analysis	Pietrzyk, P. M.	[266]
1999	MRI evaluation of post-operative seromas in extremity soft tissue sarcomas	Poon-Chue, A.; Menendez, L.; Gerstner, M. M.; Colletti, P.; Terk, M.	[267]
2014	'Real-life' information on pulmonary arterial hypertension: the iPHnet Project	Poscia, Roberto; Ghio, Stefano; D'Alto, Michele; Vitulo, Patrizio; Mule, Massimilano; Albera, Carlo; Parisi, Francesco; Badagliacca, Roberto; Fedele, Francesco; Vizza, Carmine Dario	[268]
1982	Case summaries in real-time. Accomplishments with a microcomputer	Pourriat, J. L.; Huet, B.; Gabry, A. L.; Rolland, C.; Cupa, M.	[269]
2015	Epidemiological Survey of Sinonasal Malignancy in North-East Iran	Poursadegh, Mehdi; Poursadegh, Farid; Esmaeili, Majid; Bakhshaee, Mehdi	[270]
1997	Summarizing clinical psychiatric data	Powsner, S. M.; Tufte, E. R.	[271]
2012	Designing observation charts to optimize the detection of patient deterioriation: reliance on the subjective preferences of healthcare professionals is not enough	Preece, Megan H. W.; Hill, Andrew; Horswill, Mark S.; Karamatic, Rozemary; Watson, Marcus O.	[272]
2010	A snapshot of rehabilitation referrals in rural New South Wales	Pryor, Julie	[273]
2008	Integrated multimedia electronic patient record and graph-based image information for cerebral tumors	Puentes, John; Batrancourt, Benedicte; Atif, Jamal; Angelini, Elsa; Lecornu, Laurent; Zemirline, Abdelhamid; Bloch, Isabelle; Coatrieux, Gouenou; Roux, Christian	[274]
2013	Big data framework for national E-governance plan	Rajagopalan, M. R.; Vellaipandiyan, S.	[275]
1999	Acute sensorineural hearing loss at the Otorhinolaryngology Department of the General Hospital in Subotica 1991-1996	Rakic, N.	[276]
1992	AXAUDIT - ANESTHETIC AUDIT SYSTEM	RAMAYYA, G. P.	[277]
1995	Current trends with natural language processing	Rassinoux, A. M.; Michel, P. A.; Wagner, J.; Baud, R.	[278]
1979	A cancer registry for the community radiation center	Rathe, J. C.; Elliott, P. F.	[279]
1994	From multimodality digital imaging to multimedia patient record	Ratib, O.	[280]
2009	Bayesian Network Classifier for Medical Data Analysis	Reiz, Beata; Csato, Lehel	[281]
2014	Risk factors for hospitalization after dog bite injury: a case-cohort study of emergency department visits	Rhea, Sarah; Weber, David J.; Poole, Charles; Cairns, Charles	[282]
2016	Interoperability of Relationship- and Role-Based Access Control	Rizvi, Syed Zain R.; Fong, Philip W.L.	[283]
2015	Relationship-Based Access Control for an Open-Source Medical Records System	Rizvi, Syed Zain R.; Fong, Philip W.L.; Crampton, Jason; Sellwood, James	[284]
2011	Graph Comparison by Log-Odds Score Matrices with Application to Protein Topology Analysis	Rocha, J.	[285]
1977	The use of automated ambulatory medical records	Rodnick, J. E.	[286]
1993	Developing a standard data structure for medical language--the SNOMED proposal	Rothwell, D. J.; Cote, R. A.; Cordeau, J. P.; Boisvert, M. A.	[287]
2017	Learning a Health Knowledge Graph from Electronic Medical Records	Rotmensch, Maya; Halpern, Yoni; Tlimat, Abdulhakim; Horng, Steven; Sontag, David	[288]
2016	The Association of Graph Literacy With Use of and Skills Using an Online Personal Health Record in Outpatient Veterans	Ruiz, Jorge G.; Andrade, Allen D.; Hogue, Christie; Karanam, Chandana; Akkineni, Sisir; Cevallos, David; Anam, Ramanakumar; Sharit, Joseph	[289]
2014	TongueDx: A Tongue Diagnosis for Health Care on Smartphones	Ryu, Ini; Siio, Itiro	[290]
2017	Symptoms investigation by means of formal concept analysis for enhancing medical diagnoses	Săcărea, C.; Şotropa, D.; Troancă, D.	[291]
2008	An information system for injuries from external causes (SILEX): a successful project in El Salvador	Salinas, Oscar; Cosio, Gerardo de; Clavel-Arcas, Carme; Montoya, Jeannette; Serpas, Mario; Moran de Garcia, Silvia; Concha-Eastman, Alberto	[292]

2010	Understanding quality in data from hospital diagnosis and procedures: A statistical characterization	Sato, D.; Freitas, A.	[293]
2005	Medical record linkage in health information systems by approximate string matching and clustering	Sauleau, Erik A.; Paumier, Jean-Philippe; Buemi, Antoine	[294]
2011	Integration of remote blood glucose meter upload technology into a clinical pharmacist medication therapy management service	Schenk, Robert J. [JR]; Schenk, Jenna	[295]
2013	Identifying cases of congestive heart failure from administrative data: a validation study using primary care patient records	Schultz, S. E.; Rothwell, D. M.; Chen, Z.; Tu, K.	[296]
2013	Identifying cases of congestive heart failure from administrative data: a validation study using primary care patient records	Schultz, S. E.; Rothwell, D. M.; Chen, Z.; Tu, K.	[297]
2008	Design, feasibility, and acceptability of an intervention using personal digital assistant-based self-monitoring in managing type 2 diabetes	Sevick, Mary Ann; Zickmund, Susan; Korytkowski, Mary; Piraino, Beth; Sereika, Susan; Mihalko, Shannon; Snetselaar, Linda; Stumbo, Phyllis; Hausmann, Leslie; Ren, Dianxu; Marsh, Rita; Sakraida, Teresa; Gibson, Jolynn; Safaien, Mehry; Starrett, Terry J.; Burke, Lora E.	[298]
2015	Predicting Health Outcomes from High-Dimensional Longitudinal Health Histories Using Relational Random Forests	Shahn, Zach; Ryan, Patrick; Madigan, David	[299]
2014	The Roles of Health Literacy, Numeracy, and Graph Literacy on the Usability of the VA's Personal Health Record by Veterans	Sharit, Joseph; Lisigurski, Miriam; Andrade, Allen D.; Karanam, Chandana; Nazi, Kim M.; Lewis, James R.; Ruiz, Jorge G.	[300]
2009	Algebraic Visual Analysis: The Catalano Phone Call Data Set Case Study	Shaverdian, Anna A.; Zhou, Hao; Michailidis, George; Jagadish, H. V.	[301]
2014	Validity of establishing pediatric reference intervals based on hospital patient data: a comparison of the modified Hoffmann approach to CALIPER reference intervals obtained in healthy children	Shaw, Julie L. V.; Cohen, Ashley; Konforte, Danijela; Binesh-Marvasti, Tina; Colantonio, David A.; Adeli, Khosrow	[302]
2011	Timelining: visualizing experience	Sheridan, Joanna; Chamberlain, Kerry; Dupuis, Ann	[303]
2010	Geometric Suffix Tree: Indexing Protein 3-D Structures	Shibuya, Tetsuo	[304]
2015	User Intervention Based Segmentation of Myocardium In Cardiac Cine MRI Images	Shigli, Heenakousar; Tejas, M. H.; Narayan, Lohit; Desai, Shrinivas D.	[305]
2014	Metabolic tumour volume and total lesion glycolysis, measured using preoperative 18F-FDG PET/CT, predict the recurrence of endometrial cancer	Shim, S-H; Kim, D-Y; Lee, D-Y; Lee, S-W; Park, J-Y; Lee, J. J.; Kim, J-H; Kim, Y-M; Kim, Y-T; Nam, J-H	[306]
2013	Medical home characteristics and asthma control: a prospective, observational cohort study protocol	Sills, Marion R.; Kwan, Bethany M.; Yawn, Barbara P.; Sauer, Brian C.; Fairclough, Diane L.; Federico, Monica J.; Juarez-Colunga, Elizabeth; Schilling, Lisa M.	[307]
2017	Development of a clinical decision support system for diabetes care: A pilot study	Sim, Livvi Li Wei; Ban, Kenneth Hon Kim; Tan, Tin Wee; Sethi, Sunil Kumar; Loh, Tze Ping	[308]
2015	Graphical display of diagnostic test results in electronic health records: a comparison of 8 systems	Sittig, Dean F.; Murphy, Daniel R.; Smith, Michael W.; Russo, Elise; Wright, Adam; Singh, Hardeep	[309]
1995	A MODEL FOR MEDICAL KNOWLEDGE REPRESENTATION APPLICATION TO THE ANALYSIS OF DESCRIPTIVE PATHOLOGY REPORTS	SMART, J. F.; ROUX, M.	[310]
2001	Developing indicators for emergency medical services (EMS) system evaluation and quality improvement: a statewide demonstration and planning project	Sobo, E. J.; Andriese, S.; Stroup, C.; Morgan, D.; Kurtin, P.	[311]
1999	Predictors of malignancy in childhood peripheral lymphadenopathy	Soldes, O. S.; Younger, J. G.; Hirschl, R. B.	[312]
2012	SympGraph: A Framework for Mining Clinical Notes Through Symptom Relation Graphs	Sondhi, Parikshit; Sun, Jimeng; Tong, Hanghang; Zhai, ChengXiang	[313]
2015	Visualizing collaborative electronic health record usage for hospitalized patients with heart failure	Soulakis, Nicholas D.; Carson, Matthew B.; Lee, Young Ji; Schneider, Daniel H.; Skeehan, Connor T.; Scholtens, Denise M.	[314]

2014	Sudden survival improvement in critical neurotrauma: An exploratory analysis using a stratified statistical process control technique	Sovik, Signe; Skaga, Nils Oddvar; Hanoa, Rolf; Eken, Torsten	[315]
2006	Flexible information storage in MUDR(II) EHR	Spidlen, Josef; Hanzlicek, Petr; Riha, Antonin; Zvarova, Jana	[316]
2007	Algorithms for Efficient Near-Perfect Phylogenetic Tree Reconstruction in Theory and Practice	Sridhar, Srinath; Dhamdhere, Kedar; Blelloch, Guy; Halperin, Eran; Ravi, R.; Schwartz, Russell	[317]
2015	Modeling Interoperable Information Systems with 3LGM(2) and IHE	Staubert, S.; Schaaf, M.; Jahn, F.; Brandner, R.; Winter, A.	[318]
1984	Map analyses of psychiatric services. The application of a computerized psychiatric case register to geographical analysis	Stefansson, C. G.	[319]
1989	Freedom from fat: a contemporary multi-component weight loss program for the general population of obese adults	Stevens, V. J.; Rossner, J.; Greenlick, M.; Stevens, N.; Frankel, H. M.; Craddick, S.	[320]
1996	A formal model of family medicine	Sumner, W. 2nd; Truszczynski, M.; Marek, V. W.	[321]
2014	Predicting disease associations via biological network analysis	Sun, Kai; Goncalves, Joana P.; Larminie, Chris; Przulj, Natasa	[322]
2002	Academic profiling of tobacco-related performance measures in primary care	Swartz, Susan H.; Cowan, Timothy M.; DePue, Judy; Goldstein, Michael G.	[323]
2012	Securing Provenance of Distributed Processes in an Untrusted Environment	Syalim, Amril; Nishide, Takashi; Sakurai, Kouichi	[324]
2003	Gene Expression Data Clustering and Visualization Based on a Binary Hierarchical Clustering Framework	Szeto, Lap Keung; Liew, Alan Wee-Chung; Yan, Hong; Tang, Sy-sen	[325]
2002	Menstrual diary data and menopausal transition: methodologic issues	Taffe, John; Dennerstein, Lorraine	[326]
2014	Development of a Semi-synthetic Dataset as a Testbed for Big-Data Semantic Analytics	Techentin, R.; Foti, D.; Li, P.; Daniel, E.; Gilbert, B.; Holmes, D.; Al-Saffar, S.	[327]
2014	Lessons learned from the semantic translation of healthcare data	Techentin, R.; Sauver, J. S.; Huddleston, J.; Gilbert, B.; Holmes, D.	[328]
2011	Social disparity and intrauterine death: from politics to policies	Tezcan, B.; Khazaezadeh, N.; Ash, A.; Oteng-Ntim, E.	[329]
2016	Psychiatric disorder and work life: A longitudinal study of intra-generational social mobility	Tiikkaja, Sanna; Sandin, Sven; Hultman, Christina M.; Modin, Bitte; Malki, Ninoa; Sparen, Par	[330]
2016	Society for Assisted Reproductive Technology and assisted reproductive technology in the United States: a 2016 update	Toner, James P.; Coddington, Charles C.; Doody, Kevin; van Voorhis, Brad; Seifer, David B.; Ball, G. David; Luke, Barbara; Wantman, Ethan	[331]
2016	Design of a Recommendation System for Adding Support in the Treatment of Chronic Patients	Torkar, Simon; Benedik, Peter; Rajkovic, Uros; Sustersic, Olga; Rajkovic, Vladislav	[332]
2009	Assessment of three-level selective perinatal care based on the analysis of early perinatal death rates and cesarean sections in Poland in 2008	Troszynski, Michal; Niemiec, Tomasz; Wilczynska, Anna	[333]
2015	Stabilized sparse ordinal regression for medical risk stratification	Truyen Tran; Dinh Phung; Luo, Wei; Venkatesh, Svetha	[334]
2002	Cervix uteri cancer incidence in relation to ethnic situation in Opole province, Poland	Tukiendorf, A.	[335]
2008	OPTISAS a new method to analyse patients with Sleep Apnea Syndrome	Ugon, Adrien; Philippe, Carole; Pietrasz, Slawomir; Ganascia, Jean-Gabriel; Levy, Pierre P.	[336]
2011	Conditional Anomaly Detection with Soft Harmonic Functions	Valko, Michal; Kveton, Branislav; Valizadegan, Hamed; Cooper, Gregory F.; Hauskrecht, Milos	[337]
2003	Diagnostic variability in a cohort of patients with multiple admissions in the last two decades	Vallejo, I. L.; Herrero, H. H.; Sanz, J. J.C.; Martin, J. J.D.; Azarola, E. N.	[338]
1989	Appropriate technologies for mother and child health care in developing countries	Vani, S. N.	[339]
2001	Approximately Common Patterns in Shared-forests	Vilares, M.; Ribadas, F. J.; Graña, J.	[340]
2011	Implementation and learning of laproscopic donor nephrectomy by a non-transplant general surgeon with advanced laparoscopic skills	Voskoboinik, A.; Gutman, M. J.; Croagh, D.; Bell, R.; Saunder, A.; Gribbin, J.; Kanellis, J.	[341]

2004	Ictal stuttering - A sign suggestive of psychogenic nonepileptic seizures	Vossler, D. G.; Am Haltiner; Schepp, S. K.; Friel, P. A.; Caylor, L. M.; Morgan, J. D.; Doherty, M. J.	[342]
2004	Ictal stuttering: a sign suggestive of psychogenic nonepileptic seizures	Vossler, D. G.; Haltiner, A. M.; Schepp, S. K.; Friel, P. A.; Caylor, L. M.; Morgan, J. D.; Doherty, M. J.	[343]
2002	Use of a computerized tuberculosis register for automated generation of case finding, sputum conversion, and treatment outcome reports	Vranken, R.; Coulombier, D.; Kenyon, T.; Koosimile, B.; Mavunga, T.; Coggin, W.; Binkin, N.	[344]
1991	Integration of mother and child health services in Ethiopia	Walley, J. D.; McDonald, M.	[345]
2015	Dynamic Poisson Autoregression for Influenza-Like-Illness Case Count Prediction	Wang, Zheng; Chakraborty, Prithwish; Mekaru, Sumiko R.; Brownstein, John S.; Ye, Jieping; Ramakrishnan, Naren	[346]
2016	Diagnosis Code Assignment Using Sparsity-Based Disease Correlation Embedding	Wang, Sen; Chang, Xiaojun; Li, Xue; Long, Guodong; Yao, Lina; Sheng, Quan Z.	[347]
1994	Combinatorial Pattern Discovery for Scientific Data: Some Preliminary Results	Wang, Jason Tsong-Li; Chirn, Gung-Wei; Marr, Thomas G.; Shapiro, Bruce; Shasha, Dennis; Zhang, Kaizhong	[348]
1994	Combinatorial Pattern Discovery for Scientific Data: Some Preliminary Results	Wang, Jason Tsong-Li; Chirn, Gung-Wei; Marr, Thomas G.; Shapiro, Bruce; Shasha, Dennis; Zhang, Kaizhong	[349]
2014	Constructing a Gene Team Tree in Almost O (N Lg N) Time	Wang, Biing-Feng; Lin, Chien-Hsin; Yang, I-Tse	[350]
2015	A Graph Based Methodology for Temporal Signature Identification from HER	Wang, Fei; Liu, Chuanren; Wang, Yajuan; Hu, Jianying; Yu, Guoqiang	[351]
2013	Exploring Patient Risk Groups with Incomplete Knowledge	Wang, X.; Wang, F.; Wang, J.; Qian, B.; Hu, J.	[352]
2010	Merging health literacy with computer technology: self-managing diet and fluid intake among adult hemodialysis patients	Welch, Janet L.; Siek, Katie A.; Connelly, Kay H.; Astroth, Kim S.; McManus, M. Sue; Scott, Linda; Heo, Seongkum; Kraus, Michael A.	[353]
2011	Secular trend of adhesive capsulitis	White, D.; Choi, H.; Peloquin, C.; Zhu, Y.; Zhang, Y.	[354]
2017	The Social-dance: Decomposing Naturalistic Dyadic Interaction Dynamics to the 'Micro-level'	Whyatt, Caroline P.; Torres, Elizabeth B.	[355]
2010	Adapting Recommender Systems to the Requirements of Personal Health Record Systems	Wiesner, Martin; Pfeifer, Daniel	[356]
1992	Octrees for Faster Isosurface Generation	Wilhelms, Jane; van Gelder, Allen	[357]
2007	3LGM(2)-Modeling to support management of health information systems	Winter, Alfred; Brigl, Birgit; Funkat, Gert; Haeber, Anke; Heller, Oliver; Wendt, Thomas	[358]
2012	Exploring Flow, Factors, and Outcomes of Temporal Event Sequences with the Outflow Visualization	Wongsuphasawat, Krist; Gotz, David	[359]
2009	Refining Phylogenetic Trees Given Additional Data: An Algorithm Based on Parsimony	Wu, Taoyang; Moulton, Vincent; Steel, Mike	[360]
2016	Mining Dual Networks: Models, Algorithms, and Applications	WU, Yubao; Zhu, Xiaofeng; Li, Li; Fan, Wei; Jin, Ruoming; Zhang, Xiang	[361]
2010	Mining positive and negative weighted association rules in medical records without user-specified weights based on HITS model	Xie, W.; Wu, J.	[362]
2018	Open Knowledge Accessing Method in IoT-based Hospital Information System for Medical Record Enrichment	Xie, C.; Yang, P.; Yang, Y.	[363]
2017	Graph Clustering System for Text-Based Records in a Clinical Pathway	Yamashita, Takanori; Onimura, Naoya; Soejima, Hidehisa; Nakashima, Naoki; Hirokawa, Sachio	[364]
2013	Automated system of hess screen for diagnosis of paralytic strabismus using computer aided diagnosis	Yamin, A.; Khan, S. A.; Yasin, U. U.	[365]
2015	A graph-based method for analyzing electronic medical records	Yesha, R.; Gangopadhyay, A.; Siegel, E.	[366]
2017	Deep Neural Network Based on Translation Model for Diabetes Knowledge Graph	Yin, S.; Chen, D.; Le J	[367]

2016	Visualization of Data Regarding Infections Using Eye Tracking Techniques	Yoon, Sunmoo; Cohen, Bevin; Cato, Kenrick D.; Liu, Jianfang; Larson, Elaine L.	[368]
2013	A quantitative method for assessment of prescribing patterns using electronic health records	Yoon, Dukyong; Park, Inwhee; Schuemie, Martijn J.; Park, Man Young; Kim, Ju Han; Park, Rae Woong	[369]
2009	Scenario-oriented information extraction from electronic health records	Yousefi, A.; Mastouri, N.; Sartipi, K.	[370]
2015	Characterizing chronic disease and polymedication prescription patterns from electronic health records	Zamora, M.; Baradad, M.; Amado, E.; Cordomí, S.; Limón, E.; Ribera, J.; Arias, M.; Gavaldà, R.	[371]
2006	Information Technology in Health Care Systems: Barriers to Adoption	Zenios, S.	[372]
2017	HCNN: Heterogeneous Convolutional Neural Networks for Comorbid Risk Prediction with Electronic Health Records	Zhang, J.; Gong, J.; Barnes, L.	[373]
2016	MTPGraph: A Data-Driven Approach to Predict Medical Risk Based on Temporal Profile Graph	Zhang, S.; Liu, L.; Li, H.; Xiao, Z.; Cui, L.	[374]
2013	Mining Deviations from Patient Care Pathways via Electronic Medical Record System Audits	Zhang, He; Mehotra, Sanjay; Liebovitz, David; Gunter, Carl A.; Malin, Bradley	[375]
2014	Towards personalized medicine: leveraging patient similarity and drug similarity analytics	Zhang, Ping; Wang, Fei; Hu, Jianying; Sorrentino, Robert	[376]
2015	Cluster-based Epidemic Control Through Smartphone-based Body Area Networks	Zhang, Zhaoyang; Wang, Honggang; Wang, Chonggang; Fang, Hua	[377]
2004	DBMap: a space-conscious data visualization and knowledge discovery framework for biomedical data warehouse	Zhang, Ming; Zhang, Hong; Tjandra, D.; Wong, S. T. C.	[378]
2017	Mining Medical Causality for Diagnosis Assistance	Zhao, Sendong	[379]
2016	Graph-Based Multi-Modality Learning for Clinical Decision Support	Zheng, Ziwei; Wan, Xiaojun	[380]
2006	Approaches to Text Mining for Clinical Medical Records	Zhou, Xiaohua; Han, Hyoil; Chankai, Isaac; Prestrud, Ann; Brooks, Ari	[381]
2006	Genetic Database Optimization: How Data Inspection and Consideration, Provides for Index Compression and Record Access Optimization of Genetic Databases	Zirkind, Givon	[382]
1994	MENELAS - AN ACCESS SYSTEM FOR MEDICAL RECORDS USING NATURAL-LANGUAGE	ZWEIGENBAUM, P.	[383]

References

1. Abbs v. Sullivan. Fed Report. 1992;963:918–29.
2. Adamusiak T, Shimoyama N, Shimoyama M. Next generation phenotyping using the unified medical language system. JMIR Med Inform. 2014;2:e5. doi:10.2196/medinform.3172.
3. Adin CA, Gregory CR, Kyles AE, Cowgill L. Diagnostic predictors of complications and survival after renal transplantation in cats. VETERINARY SURGERY. 2001;30:515–21. doi:10.1053/jvet.2001.28418.
4. Agogo GO, van der Voet H, van't Veer P, Ferrari P, Leenders M, Muller DC, et al. Use of two-part regression calibration model to correct for measurement error in episodically consumed foods in a single-replicate study design: EPIC case study. PLoS One. 2014;9:e113160. doi:10.1371/journal.pone.0113160.
5. Albarakati N, Obradovic Z. Disease-Based Clustering of Hospital Admission: Disease Network of Hospital Networks Approach. In: ; 2017. p. 636–641. doi:10.1109/CBMS.2017.87.
6. Allali J, Sagot M. A New Distance for High Level RNA Secondary Structure Comparison. IEEE/ACM Trans. Comput. Biol. Bioinformatics. 2005;2:3–14. doi:10.1109/TCBB.2005.2.
7. Alvarez JM, Polo L, Jimenez W, Abella P, Labra JE. Application of the Spreading Activation Technique for Recommending Concepts of Well-known Ontologies in Medical Systems. In: New York, NY, USA: ACM; 2011. p. 626–635. doi:10.1145/2147805.2147913.
8. Amit M, Yen TC, Liao CT, Chaturvedi P, Agarwal JP, Kowalski LP, et al. The origin of regional failure in oral cavity squamous cell carcinoma with pathologically negative neck metastases. JAMA Otolaryngol Head Neck Surg. 2014;140:1130–7. doi:10.1001/jamaoto.2014.1539.
9. Andersen A. An implementation of secure multi-party computations to preserve privacy when processing EMR data. In: ; 2013. p. 381–384. doi:10.1109/PST.2013.6596096.
10. Andersen A. SNOOP: Privacy Preserving Middleware for Secure Multi-party Computations. In: New York, NY, USA: ACM; 2014. p. 8:1. doi:10.1145/2677017.2677025.

11. Andersen A, Yigzaw KY, Karlsen R. Privacy preserving health data processing. In: ; 2014. p. 225–230. doi:10.1109/HealthCom.2014.7001845.
12. Andreano A, Rebora P, Valsecchi MG, Russo AG. Adherence to guidelines and breast cancer patients survival: a population-based cohort study analyzed with a causal inference approach. Breast Cancer Res Treat. 2017;164:119–31. doi:10.1007/s10549-017-4210-z.
13. Anindya IC, Roy H, Kantarcioglu M, Malin B. Building a Dossier on the Cheap: Integrating Distributed Personal Data Resources Under Cost Constraints. In: New York, NY, USA: ACM; 2017. p. 1549–1558. doi:10.1145/3132847.3132951.
14. Antani S. Integrating image and text information for biomedical information retrieval. In: ; 2010. p. 3. doi:10.1109/CBMS.2010.6042631.
15. Antonakakis M, Dimitriadis SI, Zervakis M, Micheloyannis S, Rezaie R, Babajani-Feremi A, et al. Altered cross-frequency coupling in resting-state MEG after mild traumatic brain injury. Int J Psychophysiol. 2016;102:1–11. doi:10.1016/j.ijpsycho.2016.02.002.
16. Anwar MN, Oakes MP. Data Mining of Audiology Patient Records: Factors Influencing the Choice of Hearing Aid Type. In: New York, NY, USA: ACM; 2011. p. 11–18. doi:10.1145/2064696.2064701.
17. Anwar MN, Oakes MP. Data mining of audiology patient records: factors influencing the choice of hearing aid type. BMC Med Inform Decis Mak. 2012;12 Suppl 1:S6. doi:10.1186/1472-6947-12-S1-S6.
18. Arantes WMd, Verdier C. Public health alert system for health networks: application to cardiology. In: ; 2005. p. 151–154. doi:10.1109/CIC.2005.1588057.
19. Arcilla MJB, Ang VCT, Ochoa MN, Padua CPL, Payawal RE. Kidnew: The Kidney Transplant Patient Personal Health Buddy. In: New York, NY, USA: ACM; 2015. p. 133–134. doi:10.1145/2750511.2750539.
20. Athreya AP, Ngiam KY, Luo Z, Tai ES, Kalbarczyk Z, Iyer RK. Towards Longitudinal Analysis of a Population's Electronic Health Records Using Factor Graphs. In: ; 2016. p. 79–86.
21. Atif J, Hudelot C, Nempont O, Richard N, Batrancourt B, Angelini E, Bloch I. GRAFIP: A FRAMEWORK FOR THE REPRESENTATION OF HEALTHY AND PATHOLOGICAL CEREBRAL INFORMATION. In: ; 2007. p. 205–208. doi:10.1109/ISBI.2007.356824.
22. Atreja A, Khan S, Rogers JD, Otobo E, Patel NP, Ullman T, et al. Impact of the Mobile HealthPROMISE Platform on the Quality of Care and Quality of Life in Patients With Inflammatory Bowel Disease: Study Protocol of a Pragmatic Randomized Controlled Trial. JMIR Res Protoc. 2015;4:e23. doi:10.2196/resprot.4042.
23. Bakel LA, Wilson K, Tyler A, Tham E, Reese J, Bothner J, Kaplan DW. A quality improvement study to improve inpatient problem list use. Hosp Pediatr. 2014;4:205–10. doi:10.1542/hpeds.2013-0060.
24. Baptista Macaroff WM, Castroman Espasandin P. Analgesic quality in a postoperative pain service: continuous assessment with the cumulative sum (cusum) method. Rev Esp Anestesiol Reanim. 2007;54:11–6.
25. Barsky M, Stege U, Thomo A, Upton C. A Graph Approach to the Threshold All-against-all Substring Matching Problem. J. Exp. Algorithmics. 2008;12:1.10:1. doi:10.1145/1227161.1370601.
26. Bassily MN, Wilson R, Pompei F, Burmistrov D. Cancer survival as a function of age at diagnosis: a study of the Surveillance, Epidemiology and End Results database. Cancer Epidemiol. 2010;34:667–81. doi:10.1016/j.canep.2010.04.013.
27. Bassoe C. Representing health, disorder and their transitions by digraphs. Stud Health Technol Inform. 2008;136:133–8.
28. Baud RH, Am RASSINOUX, SCHERRER, JR. NATURAL-LANGUAGE PROCESSING AND SEMANTICAL REPRESENTATION OF MEDICAL TEXTS. Methods Inf Med. 1992;31:117–25.
29. Baud RH, Am RASSINOUX, WAGNER JC, Lovis C, JUGE C, ALPAY LL, et al. REPRESENTING CLINICAL NARRATIVES USING CONCEPTUAL GRAPHS. Methods Inf Med. 1995;34:176–86.
30. Baud RH, Rassinoux AM, Scherrer JR. Natural language processing and semantical representation of medical texts. Methods Inf Med. 1992;31:117–25.
31. Bean DM, Wu H, Iqbal E, Dzahini O, Ibrahim ZM, Broadbent M, et al. Knowledge graph prediction of unknown adverse drug reactions and validation in electronic health records. Sci Rep. 2017;7:16416. doi:10.1038/s41598-017-16674-x.
32. Bean DM, Wu H, Iqbal E, Dzahini O, Ibrahim ZM, Broadbent M, et al. Author Correction: Knowledge graph prediction of unknown adverse drug reactions and validation in electronic health records. Sci Rep. 2018;8:4284. doi:10.1038/s41598-018-22521-4.
33. Beilin LJ, Bulpitt CJ, Coles EC, Dollery CT, Johnson BF, Mearns C, et al. Computer-based hypertension clinic records: a co-operative study. Br Med J. 1974;2:212–6.
34. Bellin E, Kalkut G. Is time-slice analysis superior to total hospital length of stay in demonstrating the effectiveness of a month-long intensive effort on a medicine service? Qual Manag Health Care. 2004;13:143–9.
35. Benedik P, Rajkovic U, Sustersic O. Toward the design of a nursing ontology system. Comput Inform Nurs. 2014;32:580–8. doi:10.1097/CIN.0000000000000117.
36. Bereg S, Zhang Y. Phylogenetic Networks Based on the Molecular Clock Hypothesis. IEEE/ACM Trans. Comput. Biol. Bioinformatics. 2007;4:661–7. doi:10.1109/tcbb.2007.1043.
37. Bernauer J, Franz M, Schoop D, Schoop M, Pretschner DP. The compositional approach for representing medical concept systems. Medinfo. 1995;8 Pt 1:70–4.
38. Bertrand D, Gascuel O. Topological Rearrangements and Local Search Method for Tandem Duplication Trees. IEEE/ACM Trans. Comput. Biol. Bioinformatics. 2005;2:15–28. doi:10.1109/TCBB.2005.15.

39. Bingham A. Computerized patient records benefit physician offices. Healthc Financ Manage. 1997;51:68–70.
40. Bono B de, Helvensteijn M, Kokash N, Martorelli I, Sarwar D, Islam S, et al. Requirements for the formal representation of pathophysiology mechanisms by clinicians. INTERFACE FOCUS 2016. doi:10.1098/rsfs.2015.0099.
41. Bordewich M, Gascuel O, Huber KT, Moulton V. Consistency of Topological Moves Based on the Balanced Minimum Evolution Principle of Phylogenetic Inference. IEEE/ACM Trans. Comput. Biol. Bioinformatics. 2009;6:110–7. doi:10.1109/TCBB.2008.37.
42. Brewer NT, Gilkey MB, Lillie SE, Hesse BW, Sheridan SL. Tables or bar graphs? Presenting test results in electronic medical records. Med Decis Making. 2012;32:545–53. doi:10.1177/0272989X12441395.
43. Britto MT, Jimison HB, Munafo JK, Wissman J, Rogers ML, Hersh W. Usability testing finds problems for novice users of pediatric portals. J Am Med Inform Assoc. 2009;16:660–9. doi:10.1197/jamia.M3154.
44. Brown AL. Constructing Chromosome Scale Suffix Trees. In: Darlinghurst, Australia, Australia: Australian Computer Society, Inc; 2004. p. 105–112.
45. Brunson JC, Laubenbacher RC. Applications of network analysis to routinely collected health care data: a systematic review. JOURNAL OF THE AMERICAN MEDICAL INFORMATICS ASSOCIATION. 2018;25:210–21. doi:10.1093/jamia/ocx052.
46. Buchtela D, Anger Z, Peleska J, Vesely A, Zvarova J. Presentation of medical guidelines on a computer. Stud Health Technol Inform. 2004;105:166–71.
47. Bui AAT, Taira RK, Churchill B, Kangarloo H. Integrated visualization of problemcentric urologic patient records. Ann N Y Acad Sci. 2002;980:267–77.
48. Burgess HA. Use of the labor graph in Malawi. J Nurse Midwifery. 1986;31:46–52.
49. Butch SH. Practical use of computerized hospital information systems to improve blood transfusion. Am J Clin Pathol. 1997;107:S50-6.
50. CAMPBELL KE, DAS AK, MUSEN MA. A LOGICAL FOUNDATION FOR REPRESENTATION OF CLINICAL-DATA. JOURNAL OF THE AMERICAN MEDICAL INFORMATICS ASSOCIATION. 1994;1:218–32. doi:10.1136/jamia.1994.95236154.
51. Campbell WS, Pedersen J, McClay JC, Rao P, Bastola D, Campbell JR. An alternative database approach for management of SNOMED CT and improved patient data queries. J Biomed Inform. 2015;57:350–7. doi:10.1016/j.jbi.2015.08.016.
52. Cates W, JR. Legal abortion: the public health record. Science. 1982;215:1586–90.
53. Cen S, Han L, Ma J. Ranking Weblogs by Analyzing Reading and Commenting Activities. In: Washington, DC, USA: IEEE Computer Society; 2009. p. 442–449. doi:10.1109/WI-IAT.2009.77.
54. Chan DJ, Furner V, Smith DE, Dronavalli M, Bopage RI, Post JJ, Bhardwaj AK. Non-AIDS complexity amongst patients living with HIV in Sydney: risk factors and health outcomes. AIDS Res Ther. 2018;15:6. doi:10.1186/s12981-018-0193-z.
55. Chazelle B. Technical Perspective: Finding a Good Neighbor, Near and Fast. Commun. ACM. 2008;51:115. doi:10.1145/1327452.1327493.
56. Chen L, Li X, Sheng QZ, Peng WC, Bennett J, Hu HY, Huang N. Mining Health Examination Records #x2014;A Graph-Based Approach. IEEE TRANSACTIONS ON KNOWLEDGE AND DATA ENGINEERING. 2016;28:2423–37. doi:10.1109/TKDE.2016.2561278.
57. Chen J, Poon J, Poon SK, Xu L, Sze DMY. Mining Symptom-Herb Patterns from Patient Records Using Tripartite Graph. Evid Based Complement Alternat Med. 2015;2015:435085. doi:10.1155/2015/435085.
58. Chen P, Zhao T, Feng R, Chai J, Tong G, Wang D. Patterns and trends with cancer incidence and mortality rates reported by the China National Cancer Registry. Asian Pac J Cancer Prev. 2014;15:6327–32.
59. Chennamsetty H, Chalasani S, Riley D. Predictive analytics on Electronic Health Records (EHRs) using Hadoop and Hive. In: ; 2015. p. 1–5. doi:10.1109/ICECCT.2015.7226129.
60. Choi E, Bahadori MT, Le Song, Stewart WF, Sun J. GRAM: Graph-based Attention Model for Healthcare Representation Learning. In: New York, NY, USA: ACM; 2017. p. 787–795. doi:10.1145/3097983.3098126.
61. Christinat Y, Moret BME. A Transcript Perspective on Evolution. IEEE/ACM Trans. Comput. Biol. Bioinformatics. 2013;10:1403–11. doi:10.1109/TCBB.2012.145.
62. Christofidis MJ, Hill A, Horswill MS, Watson MO. Observation charts with overlapping blood pressure and heart rate graphs do not yield the performance advantage that health professionals assume: an experimental study. J Adv Nurs. 2014;70:610–24. doi:10.1111/jan.12223.
63. Chung HH, Kim JW, Kang KW, Park N, Song Y, Chung J, Kang S. Post-treatment (1)(8)FFDG maximum standardized uptake value as a prognostic marker of recurrence in endometrial carcinoma. Eur J Nucl Med Mol Imaging. 2011;38:74–80. doi:10.1007/s00259-010-1614-y.
64. Cimino JJ, Clayton PD, Hripcsak G, Johnson SB. Knowledge-based approaches to the maintenance of a large controlled medical terminology. J Am Med Inform Assoc. 1994;1:35–50.
65. Collier T, Steenkamp R, Tomson C, Caskey F, Ansell D, Roderick P, Nitsch D. Patterns and effects of missing comorbidity data for patients starting renal replacement therapy in England, Wales and Northern Ireland. Nephrol Dial Transplant. 2011;26:3651–8. doi:10.1093/ndt/gfr111.

66. Cota E, Ribeiro L, Bezerra JS, Costa A, da Silva RE, Cota G. Using formal methods for content validation of medical procedure documents. Int J Med Inform. 2017;104:10–25. doi:10.1016/j.ijmedinf.2017.04.012.
67. Cruz JP, Libatique NJ, Tangonan G. Steganography and data hiding in flash video (FLV). In: ; 2012. p. 1–6. doi:10.1109/TENCON.2012.6412279.
68. Cypko MA, Wojdziak J, Stoehr M, Kirchner B, Preim B, Dietz A, et al. Visual Verification of Cancer Staging for Therapy Decision Support. COMPUTER GRAPHICS FORUM. 2017;36:109–20. doi:10.1111/cgf.13172.
69. da Silva Klahr P, Correa Coronel C, Cabral Robinson C, Fonseca JM, Dias Flores C, Della Mea Plentz R. Influence Diagram As a Support Tool for Clinical Decisions In Cardiopulmonary And Metabolic Rehabilitation. Stud Health Technol Inform. 2015;216:290–4.
70. Dabek F, Caban JJ. A grammar-based approach to model the patient's clinical trajectory after a mild traumatic brain injury. In: ; 2015. p. 723–730. doi:10.1109/BIBM.2015.7359775.
71. Dabek F, Chen J, Garbarino A, Caban JJ. Visualization of Longitudinal Clinical Trajectories Using a Graph-based Approach. In: New York, NY, USA: ACM; 2015. p. 5:1. doi:10.1145/2836034.2836039.
72. Dahl MR, Vedsted P. Personal data and confidentiality on the Internet. Ugeskr Laeger. 2008;170:4027–9.
73. Danaei G, Garcia Rodriguez LA, Fernandez Cantero O, Logan R, Hernan MA. Observational data for comparative effectiveness research: An emulation of randomised trials of statins and primary prevention of coronary heart disease. STATISTICAL METHODS IN MEDICAL RESEARCH. 2013;22:70–96. doi:10.1177/0962280211403603.
74. Datla V, Hasan SA, Qadir A, Lee K, Ling Y, Liu J, Farri O. Automated clinical diagnosis: The role of content in various sections of a clinical document. In: ; 2017. p. 1004–1011. doi:10.1109/BIBM.2017.8217794.
75. Davidson S, Natan D, Novikov I, Sokolover N, Erlich A, Shamir R. Body mass index and weight-for-length ratio references for infants born at 33-42 weeks gestation: a new tool for anthropometric assessment. Clin Nutr. 2011;30:634–9. doi:10.1016/j.clnu.2011.03.010.
76. Degnan JH, Rosenberg NA, Stadler T. A Characterization of the Set of Species Trees That Produce Anomalous Ranked Gene Trees. IEEE/ACM Trans. Comput. Biol. Bioinformatics. 2012;9:1558–68. doi:10.1109/TCBB.2012.110.
77. Delman BS. A problem-oriented approach to journal selection for hospital libraries. Bull Med Libr Assoc. 1982;70:397–410.
78. Dent RM, Penwarden RM, Harris N, Hotz SB. Development and evaluation of patient-centered software for a weight-management clinic. OBESITY RESEARCH. 2002;10:651–6. doi:10.1038/oby.2002.88.
79. Dhulekar N, Oztan B, Yener B, Bingol HO, Irim G, Aktekin B, Aykut-Bingöl C. Graph-theoretic Analysis of Epileptic Seizures on Scalp EEG Recordings. In: New York, NY, USA: ACM; 2014. p. 155–163. doi:10.1145/2649387.2649423.
80. Dietz KR, Zhang L, Seidel FG. The transverse diameter of the chest on routine radiographs reliably estimates gestational age and weight in premature infants. Pediatr Radiol. 2015;45:1303–7. doi:10.1007/s00247-015-3332-x.
81. Diprose GK, Evans DH, Levene MI. A microcomputer monitoring and data-acquisition system for intensive care units. J Med Eng Technol. 1985;9:80–4.
82. Edsberg O, Nordbo SJ, Vinnes E, Nytro O. Design and evaluation of a temporal, graph-based language for querying collections of patient histories. Stud Health Technol Inform. 2007;129:402–6.
83. Elliott D, Allen E, McKinley S, Perry L, Duffield C, Fry M, et al. User acceptance of observation and response charts with a track and trigger system: a multisite staff survey. J Clin Nurs. 2016;25:2211–22. doi:10.1111/jocn.13303.
84. Engholm G, Ferlay J, Christensen N, Bray F, Gjerstorff ML, Klint A, et al. NORDCAN--a Nordic tool for cancer information, planning, quality control and research. Acta Oncol. 2010;49:725–36. doi:10.3109/02841861003782017.
85. Erkal S, Gerberich SG, Ryan AD, Renier CM, Alexander BH. Animal-related injuries: a population-based study of a five-state region in the upper Midwest: Regional Rural Injury Study II. J Safety Res. 2008;39:351–63. doi:10.1016/j.jsr.2008.03.002.
86. Esteban C, Schmidt D, Krompaß D, Tresp V. Predicting Sequences of Clinical Events by Using a Personalized Temporal Latent Embedding Model. In: ; 2015. p. 130–139. doi:10.1109/ICHI.2015.23.
87. Evandt J, Oftedal B, Krog NH, Skurtveit S, Nafstad P, Schwarze PE, et al. Road traffic noise and registry based use of sleep medication. Environ Health. 2017;16:110. doi:10.1186/s12940-017-0330-5.
88. Evans JM, MacDonald TM. Misclassification and selection bias in case-control studies using an automated database. Pharmacoepidemiol Drug Saf. 1997;6:313–8. doi:10.1002/(SICI)1099-1557(199709/10)6:5<313::AID-PDS292>3.0.CO;2-K.
89. Faeder JR, Blinov ML, Hlavacek WS. Graphical Rule-based Representation of Signal-transduction Networks. In: New York, NY, USA: ACM; 2005. p. 133–140. doi:10.1145/1066677.1066712.
90. Faloutsos C. Future Directions in Data Mining: Streams, Networks, Self-similarity and Power Laws. In: New York, NY, USA: ACM; 2002. p. 93. doi:10.1145/584792.584794.

91. Faloutsos C, Kamel I. Beyond Uniformity and Independence: Analysis of R-trees Using the Concept of Fractal Dimension. In: New York, NY, USA: ACM; 1994. p. 4–13. doi:10.1145/182591.182593.
92. Fan K, Eisenberg M, Walsh A, Aiello A, Heller K. Hierarchical Graph-Coupled HMMs for Heterogeneous Personalized Health Data. In: New York, NY, USA: ACM; 2015. p. 239–248. doi:10.1145/2783258.2783326.
93. Farfan F, Hristidis V, Ranganathan A, Burke RP. Ontology-Aware Search on XML-based Electronic Medical Records. In: ; 2008. p. 1525–1527. doi:10.1109/ICDE.2008.4497611.
94. Fazeli Dehkordy S, Carlos RC, Hall KS, Dalton VK. Novel data sources for women's health research: mapping breast screening online information seeking through Google trends. Acad Radiol. 2014;21:1172–6. doi:10.1016/j.acra.2014.05.005.
95. Feng J, Zhu D. Faster Algorithms for Sorting by Transpositions and Sorting by Block Interchanges. ACM Trans. Algorithms 2007. doi:10.1145/1273340.1273341.
96. Ferreira TS, Luiz de Souza-Braga A, Soraia Cavalcanti-Valente G, Ferreira de Souza D, Moreira Carvalho-Alves E. Nursing audit: The impact of nursing annotation in the context of hospital gloss. AQUICHAN. 2009;9:38–49.
97. Finlayson SG, LePendu P, Shah NH. Building the graph of medicine from millions of clinical narratives. Sci Data. 2014;1:140032. doi:10.1038/sdata.2014.32.
98. Finney JM, Walker AS, Peto TEA, Wyllie DH. An efficient record linkage scheme using graphical analysis for identifier error detection. BMC Med Inform Decis Mak. 2011;11:7. doi:10.1186/1472-6947-11-7.
99. Flack JR. Seven years experience with a computerized diabetes clinic database. Medinfo. 1995;8 Pt 1:332.
100. Florit AM, Mori AR, Simone M de, d'Annunzio V, Ricci FL, Lalle C. Context trees: representing co-operative healthcare activities in IREP. Comput Methods Programs Biomed. 1995;48:175–81.
101. Folio LR, Machado LB, Dwyer AJ. Multimedia-enhanced Radiology Reports: Concept, Components, and Challenges. Radiographics. 2018;38:462–82. doi:10.1148/rg.2017170047.
102. Forister JFY, Sun A, Weissman BA. Progress report on a post-radial keratotomy patient 20 years after surgery. Eye Contact Lens. 2007;33:334–7. doi:10.1097/ICL.0b013e318030f1b6.
103. Fors UG, Sandberg HC. Computer-aided risk management--a software tool for the Hidep model. Quintessence Int. 2001;32:309–20.
104. Fraccaro P, Vigo M, Balatsoukas P, van der Veer, Sabine N., Hassan L, Williams R, et al. Presentation of laboratory test results in patient portals: influence of interface design on risk interpretation and visual search behaviour. BMC Med Inform Decis Mak 2018. doi:10.1186/s12911-018-0589-7.
105. Frazier GF. An Incremental Algorithm for Building Temporal Quadtrees. In: New York, NY, USA: ACM; 1993. p. 446–452. doi:10.1145/170791.170896.
106. Friesen MR, Hamel C, McLeod RD. A mHealth application for chronic wound care: findings of a user trial. Int J Environ Res Public Health. 2013;10:6199–214. doi:10.3390/ijerph10116199.
107. Gail MH, Costantino JP, Pee D, Bondy M, Newman L, Selvan M, et al. Projecting individualized absolute invasive breast cancer risk in African American women. J Natl Cancer Inst. 2007;99:1782–92. doi:10.1093/jnci/djm223.
108. Galopin A, Bouaud J, Pereira S, Seroussi B. Using an ontological modeling to evaluate the consistency of clinical practice guidelines: application to the comparison of three guidelines on the management of adult hypertension. Stud Health Technol Inform. 2014;205:38–42.
109. Galopin A, Bouaud J, Pereira S, Seroussi B. An Ontology-Based Clinical Decision Support System for the Management of Patients with Multiple Chronic Disorders. Stud Health Technol Inform. 2015;216:275–9.
110. Garla V, Taylor C, Brandt C. Semi-supervised clinical text classification with Laplacian SVMs: an application to cancer case management. J Biomed Inform. 2013;46:869–75. doi:10.1016/j.jbi.2013.06.014.
111. Garrett WF, Fuchs H, Whitton MC, State A. Real-time Incremental Visualization of Dynamic Ultrasound Volumes Using Parallel BSP Trees. In: Los Alamitos, CA, USA: IEEE Computer Society Press; 1996. p. 235-ff.
112. Gerson CD, Gerson M. Technical report: an ePRO patient reported outcome program for the evaluation of patients with irritable bowel syndrome. Neurogastroenterol Motil. 2014;26:290–4. doi:10.1111/nmo.12255.
113. Gevins AS, Yeager CL, Diamond SL. Interactive Analysis and Display of the Electroencephalogram (EEG) in Real Time. In: New York, NY, USA: ACM; 1974. p. 75–76. doi:10.1145/563182.563233.
114. Ghosh P, Kalyanaraman A. A Fast Sketch-based Assembler for Genomes. In: New York, NY, USA: ACM; 2016. p. 241–250. doi:10.1145/2975167.2975192.
115. Giani U, Brascio G, Bruzzese D, Garzillo C, Vigilante S. Emotional and cognitive information processing in web-based medical education. J Biomed Inform. 2007;40:332–42. doi:10.1016/j.jbi.2006.11.004.
116. Gibson TA, Goldberg DS. Evaluating Theoretical Models of Protein Interaction Network Evolution Without Seed Graphs. In: New York, NY, USA: ACM; 2013. p. 724:724. doi:10.1145/2506583.2512388.
117. Glinkowski W. Web-Based Support for Fracture Healing Evaluation and Monitoring. TELEMEDICINE AND E-HEALTH. 2011;17:201–10. doi:10.1089/tmj.2010.0131.
118. Goldstein A, Shahar Y. An automated knowledge-based textual summarization system for longitudinal, multivariate clinical data. J Biomed Inform. 2016;61:159–75. doi:10.1016/j.jbi.2016.03.022.

119. Goodwin T, Harabagiu SM. Automatic Generation of a Qualified Medical Knowledge Graph and Its Usage for Retrieving Patient Cohorts from Electronic Medical Records. In: ; 2013. p. 363–370. doi:10.1109/ICSC.2013.68.
120. Goodwin T, Harabagiu S. Embedding Open-domain Common-sense Knowledge from Text. LREC Int Conf Lang Resour Eval. 2016;2016:4621–8.
121. Goodwin TR, Harabagiu SM. Medical Question Answering for Clinical Decision Support. Proc ACM Int Conf Inf Knowl Manag. 2016;2016:297–306. doi:10.1145/2983323.2983819.
122. Goodwin TR, Harabagiu SM. Knowledge Representations and Inference Techniques for Medical Question Answering. ACM TRANSACTIONS ON INTELLIGENT SYSTEMS AND TECHNOLOGY 2018. doi:10.1145/3106745.
123. Gopakumar S, Tran T, Nguyen TD, Phung D, Venkatesh S. Stabilizing high-dimensional prediction models using feature graphs. IEEE J Biomed Health Inform. 2015;19:1044–52. doi:10.1109/JBHI.2014.2353031.
124. Gortzis LG, Nikiforidis G. Tracing and cataloguing knowledge in an e-health cardiology environment. J Biomed Inform. 2008;41:217–23. doi:10.1016/j.jbi.2007.09.001.
125. Grinspan ZM, Abramson EL, Banerjee S, Kern LM, Kaushal R, Shapiro JS. Potential value of health information exchange for people with epilepsy: crossover patterns and missing clinical data. AMIA Annu Symp Proc. 2013;2013:527–36.
126. Gunter C. Detecting Roles and Anomalies in Hospital Access Audit Logs. In: New York, NY, USA: ACM; 2014. p. 1. doi:10.1145/2665936.2668879.
127. Guzzi PH, Mina M. Towards the Assessment of Semantic Similarity Analysis of Protein Data: Main Approaches and Issues. ACM SIGBioinformatics Rec. 2012;2:17–8. doi:10.1145/2384691.2384694.
128. Hackney DN, Knudtson EJ, Rossi KQ, Krugh D, O'Shaughnessy RW. Management of pregnancies complicated by anti-c isoimmunization. Obstet Gynecol. 2004;103:24–30. doi:10.1097/01.AOG.0000109206.22354.2C.
129. Halachev M, Shiri N, Thamildurai A. Exact Match Search in Sequence Data Using Suffix Trees. In: New York, NY, USA: ACM; 2005. p. 123–130. doi:10.1145/1099554.1099579.
130. Hanzlicek P, Spidlen J, Nagy M. Universal electronic health record MUDR. Stud Health Technol Inform. 2004;105:190–201.
131. Hanzlicek P, Spidlen J, Heroutova H, Nagy M. User interface of MUDR electronic health record. Int J Med Inform. 2005;74:221–7. doi:10.1016/j.ijmedinf.2004.06.003.
132. Harabagiu S. Big mechanisms for processing big data in medical informatics. In: ; 2015. p. 1–2. doi:10.1109/SPMB.2015.7405440.
133. Harris J. Next Generation Linkage Management System. In: Darlinghurst, Australia, Australia: Australian Computer Society, Inc; 2013. p. 7–12.
134. Harsola A, Thale S, Panse MS. Low Cost Digital Stethoscope for Heart Sounds. In: New York, NY, USA: ACM; 2011. p. 1275–1279. doi:10.1145/1980022.1980304.
135. Hartzler AL, Izard JP, Dalkin BL, Mikles SP, Gore JL. Design and feasibility of integrating personalized PRO dashboards into prostate cancer care. JOURNAL OF THE AMERICAN MEDICAL INFORMATICS ASSOCIATION. 2016;23:38–47. doi:10.1093/jamia/ocv101.
136. Hausler R, Washburn D, Rey P, Stefanoff F. Acquiring otoneurological results by computer. Evaluation after 2 years of experience. Ann Otolaryngol Chir Cervicofac. 1982;99:403–8.
137. Heer J, Perer A. Orion: A system for modeling, transformation and visualization of multidimensional heterogeneous networks. INFORMATION VISUALIZATION. 2014;13:111–33. doi:10.1177/1473871612462152.
138. Henry SB, Mead CN. Nursing classification systems: necessary but not sufficient for representing "what nurses do" for inclusion in computer-based patient record systems. J Am Med Inform Assoc. 1997;4:222–32.
139. Henzinger MR, King V, Warnow T. Constructing a Tree from Homeomorphic Subtrees, with Applications to Computational Evolutionary Biology. In: Philadelphia, PA, USA: Society for Industrial and Applied Mathematics; 1996. p. 333–340.
140. Herman T, Pemmaraju SV, Segre AM, Polgreen PM, Curtis DE, Fries J, et al. Wireless Applications for Hospital Epidemiology. In: New York, NY, USA: ACM; 2009. p. 45–50. doi:10.1145/1540373.1540384.
141. Herskovic JR, Subramanian D, Cohen T, Bozzo-Silva PA, Bearden CF, Bernstam EV. Graph-based signal integration for high-throughput phenotyping. BMC Bioinformatics. 2012;13 Suppl 13:S2. doi:10.1186/1471-2105-13-S13-S2.
142. Hettne K, Soiland-Reyes S, Klyne G, Belhajjame K, Gamble M, Bechhofer S, et al. Workflow Forever: Semantic Web Semantic Models and Tools for Preserving and Digitally Publishing Computational Experiments. In: New York, NY, USA: ACM; 2012. p. 36–37. doi:10.1145/2166896.2166909.
143. Hofer C, Kiechl S, Lang W. The Austrian Stroke-Unit-Registry. Wien Med Wochenschr. 2008;158:411–7. doi:10.1007/s10354-008-0563-6.
144. Hoimyr H, Christensen TD, Emmertsen K, Johnsen SP, Riis A, Hansen OK, Hjortdal VE. Surgical repair of coarctation of the aorta: up to 40 years of follow-up. Eur J Cardiothorac Surg. 2006;30:910–6. doi:10.1016/j.ejcts.2006.09.016.
145. Honglan L, Xiaona Q, Bin F. The Symptoms and Pathogenesis Entity Recognition of TCM Medical Records Based on CRF. In: ; 2015. p. 1479–1484. doi:10.1109/UIC-ATC-ScalCom-CBDCom-IoP.2015.267.

146. Hripcsak G, Soulakis ND, Li L, Morrison FP, Lai AM, Friedman C, et al. Syndromic surveillance using ambulatory electronic health records. J Am Med Inform Assoc. 2009;16:354–61. doi:10.1197/jamia.M2922.
147. Huang H, Gong T, Ye N, Wang R, Dou Y. Private and Secured Medical Data Transmission and Analysis for Wireless Sensing Healthcare System. IEEE Transactions on Industrial Informatics. 2017;13:1227–37. doi:10.1109/TII.2017.2687618.
148. Huesch MD, Schetter S, Segel J, Chetlen A. Evaluation of the "Angelina Jolie Effect" on Screening Mammography Utilization in an Academic Center. JOURNAL OF THE AMERICAN COLLEGE OF RADIOLOGY. 2017;14:1020–6. doi:10.1016/j.jacr.2017.03.016.
149. Huet B, Artigou JY, Poirier J, Blain G. Meta-modelling: the appropriate solution for a family of applications. Stud Health Technol Inform. 2001;84:623–7.
150. Huet B, Pourriat JL, Martin J, Cupa M. An automaton computer program for a microcomputerized real-time (thesaurus based) abstract medical record. Comput Programs Biomed. 1982;15:117–23.
151. Islam SKH, Khan MK, Li X. Security Analysis and Improvement of 'a More Secure Anonymous User Authentication Scheme for the Integrated EPR Information System'. PLoS One 2015. doi:10.1371/journal.pone.0131368.
152. Italian cancer figures, report 2012: Cancer in children and adolescents. Epidemiol Prev. 2013;37:1–225.
153. Jacky JP, Kalet IJ. An Object-oriented Programming Discipline for Standard Pascal. Commun. ACM. 1987;30:772–6. doi:10.1145/30401.30403.
154. Jamakovic M, Baljic R. Significance of copper level in serum and routine laboratory parameters in estimation of outspreading of Hodgkin's lymphoma. Med Arch. 2013;67:185–7.
155. Jamil HM, Modica GA, Teran MA. Towards a Visual Query Interface for Phylogenetic Databases. In: New York, NY, USA: ACM; 2001. p. 57–64. doi:10.1145/502585.502596.
156. Jamison RN, Jurcik DC, Edwards RR, Huang C, Ross EL. A Pilot Comparison of a Smartphone App With or Without 2-Way Messaging Among Chronic Pain Patients: Who Benefits From a Pain App? Clin J Pain. 2017;33:676–86. doi:10.1097/AJP.0000000000000455.
157. Jeunemaitre X, Degoulet P, Morice V, Chatellier G, Devries C, Plouin PF, et al. Testing an expert system for hypertension. Arch Mal Coeur Vaiss. 1986;79:808–12.
158. Ji X, Ae Chun S, Geller J. Predicting Comorbid Conditions and Trajectories using Social Health Records. IEEE Trans Nanobioscience 2016. doi:10.1109/TNB.2016.2564299.
159. Jiang J, Li X, Zhao C, Guan Y, Yu Q. Learning and inference in knowledge-based probabilistic model for medical diagnosis. KNOWLEDGE-BASED SYSTEMS. 2017;138:58–68. doi:10.1016/j.knosys.2017.09.030.
160. Jing X, Cimino JJ. A complementary graphical method for reducing and analyzing large data sets. Case studies demonstrating thresholds setting and selection. Methods Inf Med. 2014;53:173–85. doi:10.3414/ME13-01-0075.
161. Jupin J, Shi JY. Identity Tracking in Big Data: Preliminary Research Using In-Memory Data Graph Models for Record Linkage and Probabilistic Signature Hashing for Approximate String Matching in Big Health and Human Services Databases. In: New York, NY, USA: ACM; 2014. p. 20:1. doi:10.1145/2640087.2644170.
162. Kamkar I, Gupta S, Li C, Phung D, Venkatesh S. Stable clinical prediction using graph support vector machines. In: ; 2016. p. 3332–3337. doi:10.1109/ICPR.2016.7900148.
163. Kaur K, Rani R. Managing Data in Healthcare Information Systems: Many Models, One Solution. Computer. 2015;48:52–9. doi:10.1109/MC.2015.77.
164. Kavuluru R, Han S, Harris D. Unsupervised Extraction of Diagnosis Codes from EMRs Using Knowledge-Based and Extractive Text Summarization Techniques. Adv Artif Intell (2013). 2013;7884:77–88. doi:10.1007/978-3-642-38457-8_7.
165. Kawada T, Aoki S, Suzuki S. Management of personal health examination data for a population by use of a portable computer. Nihon Koshu Eisei Zasshi. 1992;39:105–7.
166. Kay M, Choe EK, Shepherd J, Greenstein B, Watson N, Consolvo S, Kientz JA. Lullaby: A Capture & Access System for Understanding the Sleep Environment. In: New York, NY, USA: ACM; 2012. p. 226–234. doi:10.1145/2370216.2370253.
167. Kazmierczak SC, Leen TK, Erdogmus D, Carreira-Perpinan MA. Reduction of multi-dimensional laboratory data to a two-dimensional plot: a novel technique for the identification of laboratory error. Clin Chem Lab Med. 2007;45:749–52. doi:10.1515/CCLM.2007.177.
168. Keenan GM, Lopez KD, Yao Y, Sousa VEC, Stifter J, Febretti A, et al. Toward Meaningful Care Plan Clinical Decision Support: Feasibility and Effects of a Simulated Pilot Study. Nurs Res. 2017;66:388–98. doi:10.1097/NNR.0000000000000234.
169. Kennedy I, Ritter H. Antenatal records: do they help us? A new record for watching fetal growth. Trop Doct. 1984;14:130–2. doi:10.1177/004947558401400310.
170. Kennedy I, Stephens B. A novel antenatal record to help midwives. Practitioner. 1979;223:18–24.
171. Kerschberger B, Hilderbrand K, Boulle AM, Coetzee D, Goemaere E, Azevedo V de, van Cutsem G. The effect of complete integration of HIV and TB services on time to initiation of antiretroviral therapy: a before-after study. PLoS One. 2012;7:e46988. doi:10.1371/journal.pone.0046988.
172. Khan IA. Personalized Electronic Health Record System for Monitoring Patients with Chronic Disease. In: ; 2013. p. 121–126. doi:10.1109/SIEDS.2013.6549505.
173. Khan A, Bhowmick SS, Bonchi F. Summarizing Static and Dynamic Big Graphs. Proc. VLDB Endow. 2017;10:1981–4. doi:10.14778/3137765.3137825.
174. Khan A, Elnikety S. Systems for Big-graphs. Proc. VLDB Endow. 2014;7:1709–10. doi:10.14778/2733004.2733067.

175. Klann JG, McCoy AB, Wright A, Wattanasin N, Sittig DF, Murphy SN. Health care transformation through collaboration on open-source informatics projects: integrating a medical applications platform, research data repository, and patient summarization. Interact J Med Res. 2013;2:e11. doi:10.2196/ijmr.2454.
176. Klann JG, Szolovits P, Downs SM, Schadow G. Decision support from local data: creating adaptive order menus from past clinician behavior. J Biomed Inform. 2014;48:84–93. doi:10.1016/j.jbi.2013.12.005.
177. Kofler J, Pesenhofer R, Landl G, Sommerfeld-Stur I, Peham C. Monitoring of dairy cow claw health status in 15 herds using the computerised documentation program Claw Manager and digital parameters. Tierarztl Prax Ausg G Grosstiere Nutztiere. 2013;41:31–44.
178. Koopman B. Semantic Search As Inference: Applications in Health Informatics. SIGIR Forum. 2014;48:116–7. doi:10.1145/2701583.2701601.
179. Koopman B, Zuccon G, Bruza P, Sitbon L, Lawley M. Graph-based Concept Weighting for Medical Information Retrieval. In: New York, NY, USA: ACM; 2012. p. 80–87. doi:10.1145/2407085.2407096.
180. Kourtis G, Caiaffa M, Forte C, Scarlato MI, Macchia L. Retrospective monitoring in the management of persistent asthma. Respir Care. 2011;56:633–43. doi:10.4187/respcare.00796.
181. Kraus S, Castellanos I, Albermann M, Schuettler C, Prokosch H, Staudigel M, Toddenroth D. Using Arden Syntax for the Generation of Intelligent Intensive Care Discharge Letters. Stud Health Technol Inform. 2016;228:471–5.
182. Kushniruk A, Borycki E, Anderson JG, Anderson MM. Combining Two Forms of Simulation to Predict the Potential Impact of Interface Design on Technology-induced Error in Healthcare. In: San Diego, CA, USA: Society for Computer Simulation International; 2008. p. 497–504.
183. Kwon Y, Kang K, Bae C, Chung H, Kim JH. Lifelog agent for human activity pattern analysis on health avatar platform. Healthc Inform Res. 2014;20:69–75. doi:10.4258/hir.2014.20.1.69.
184. Labarre A, Verwer S. Merging Partially Labelled Trees: Hardness and a Declarative Programming Solution. IEEE/ACM Trans. Comput. Biol. Bioinformatics. 2014;11:389–97. doi:10.1109/TCBB.2014.2307200.
185. Lancia G, Carr R, Walenz B, Istrail S. 101 Optimal PDB Structure Alignments: A Branch-and-cut Algorithm for the Maximum Contact Map Overlap Problem. In: New York, NY, USA: ACM; 2001. p. 193–202. doi:10.1145/369133.369199.
186. Landes SJ, Carlson EB, Ruzek JI, Wang D, Hugo E, DeGaetano N, et al. Provider-Driven Development of a Measurement Feedback System to Enhance Measurement-Based Care in VA Mental Health. COGNITIVE AND BEHAVIORAL PRACTICE. 2015;22:87–100. doi:10.1016/j.cbpra.2014.06.004.
187. Lee HJ, Kim JY, Kim SK, Lee JR, Suh CS, Kim SH. Learning Curve Analysis and Surgical Outcomes of Single-port Laparoscopic Myomectomy. J Minim Invasive Gynecol. 2015;22:607–11. doi:10.1016/j.jmig.2015.01.009.
188. Levin RJ. Incidence of thyroid cancer in residents surrounding the Three Mile Island nuclear facility. Laryngoscope. 2008;118:618–28. doi:10.1097/MLG.0b013e3181613ad2.
189. Lezcano L, Sanchez-Alonso S, Sicilia M. Associating clinical archetypes through UMLS Metathesaurus term clusters. J Med Syst. 2012;36:1249–58. doi:10.1007/s10916-010-9586-9.
190. Liabsuetrakul T, Prappre T, Pairot P, Oumudee N, Islam M. Development of a web-based epidemiological surveillance system with health system response for improving maternal and newborn health: Field-testing in Thailand. Health Informatics J. 2017;23:109–23. doi:10.1177/1460458216628312.
191. Lieberman MD, Taheri S, Guo w, Mirrashed F, Yahav I, Aris A, Shneiderman B. Visual Exploration Across Biomedical Databases. IEEE/ACM Trans. Comput. Biol. Bioinformatics. 2011;8:536–50. doi:10.1109/TCBB.2010.1.
192. Lin YL, Guerguerian A, Tomasi J, Laussen P, Trbovich P. Usability of data integration and visualization software for multidisciplinary pediatric intensive care: a human factors approach to assessing technology. BMC Med Inform Decis Mak 2017. doi:10.1186/s12911-017-0520-7.
193. Lin H, Wang Y, Jing L, Chang P. Mockup design of personal health diary app for patients with chronic kidney disease. Stud Health Technol Inform. 2014;201:124–32.
194. Liu W, Chung BC, Wang R, Ng J, Morlet N. A genetic algorithm enabled ensemble for unsupervised medical term extraction from clinical letters. Health Inf Sci Syst. 2015;3:5. doi:10.1186/s13755-015-0013-y.
195. Liu H, Dou D, Jin R, Lependu P, Shah N. Mining Biomedical Ontologies and Data Using RDF Hypergraphs. In: ; 2013. p. 141–146. doi:10.1109/ICMLA.2013.31.
196. Liu Y, Guo Z, Ke X, Za\"ıane OR. Protein Subcellular Localization Prediction with Associative Classification and Multi-class SVM. In: New York, NY, USA: ACM; 2011. p. 493–495. doi:10.1145/2147805.2147880.
197. Liu C, Wang F, Hu J, Xiong H. Temporal Phenotyping from Longitudinal Electronic Health Records: A Graph Based Framework. In: New York, NY, USA: ACM; 2015. p. 705–714. doi:10.1145/2783258.2783352.
198. Liu JM, Wu HW, Chen WS, Lin WC, Chao Y, Lui WY, Whang-Peng J. Integration of computer-assembled digital images and text data as evidence for the oncological record. J Digit Imaging. 2000;13:55–9.
199. Loorak MH, Perin C, Kamal N, Hill M, Carpendale S. TimeSpan: Using Visualization to Explore Temporal Multi-dimensional Data of Stroke Patients. IEEE Trans Vis Comput Graph. 2016;22:409–18. doi:10.1109/TVCG.2015.2467325.

200. Lopez KD, Wilkie DJ, Yao Y, Sousa V, Febretti A, Stifter J, et al. Nurses' Numeracy and Graphical Literacy: Informing Studies of Clinical Decision Support Interfaces. J Nurs Care Qual. 2016;31:124–30. doi:10.1097/NCQ.0000000000000149.
201. Lovis C, Baud RH, Revillard C, Pult L, Borst F, Geissbuhler A. Paragraph-oriented structure for narratives in medical documentation. Stud Health Technol Inform. 2001;84:638–42.
202. Luo Y, Xin Y, Hochberg E, Joshi R, Uzuner O, Szolovits P. Subgraph augmented non-negative tensor factorization (SANTF) for modeling clinical narrative text. J Am Med Inform Assoc. 2015;22:1009–19. doi:10.1093/jamia/ocv016.
203. Luque-Fernandez MA, Zoega H, Valdimarsdottir U, Williams MA. Deconstructing the smoking-preeclampsia paradox through a counterfactual framework. Eur J Epidemiol. 2016;31:613–23. doi:10.1007/s10654-016-0139-5.
204. Luz S, Kane B. Classification of Patient Case Discussions Through Analysis of Vocalisation Graphs. In: New York, NY, USA: ACM; 2009. p. 107–114. doi:10.1145/1647314.1647334.
205. Madkour M, Song H, Du Jingcheng, Tao C. A representational analysis of a temporal indeterminancy display in clinical events. In: ; 2016. p. 1089–1095. doi:10.1109/BIBM.2016.7822673.
206. Mandal AK, Sarkar A. Formal representation of service interactions for SaaS based applications. In: ; 2014. p. 54–59. doi:10.1109/ICADIWT.2014.6814700.
207. Manzi de Arantes, Wilmondes Jr, Verdier C. Defining quality-measurable medical alerts from incomplete data through fuzzy linguistic variables and modifiers. IEEE Trans Inf Technol Biomed. 2010;14:916–22. doi:10.1109/TITB.2009.2020063.
208. Margusino-Framinan L, Cid-Silva P, Mena-de-Cea A, Sanclaudio-Luhia AI, Castro-Castro JA, Vazquez-Gonzalez G, Martin-Herranz I. Intelligent MONitoring System for antiviral pharmacotherapy in patients with chronic hepatitis C (SiMON-VC). Farm Hosp. 2017;41:68–88. doi:10.7399/fh.2017.41.1.10590.
209. Markazi-Moghaddam N, Arab M, Ravaghi H, Rashidian A, Khatibi T, Zargar Balaye Jame S. A Knowledge Map for Hospital Performance Concept: Extraction and Analysis: A Narrative Review Article. Iran J Public Health. 2016;45:843–54.
210. Martin GS, Tapsell LC, Batterham MJ, Russell KG. Relative bias in diet history measurements: a quality control technique for dietary intervention trials. Public Health Nutr. 2002;5:537–45. doi:10.1079/PHN2002329.
211. Martinez D, Otegi A, Soroa A, Agirre E. Improving search over Electronic Health Records using UMLS-based query expansion through random walks. J Biomed Inform. 2014;51:100–6. doi:10.1016/j.jbi.2014.04.013.
212. Martinez-Costa C, Schulz S. Validating EHR clinical models using ontology patterns. J Biomed Inform. 2017;76:124–37. doi:10.1016/j.jbi.2017.11.001.
213. Matthews SJ. Heterogeneous Compression of Large Collections of Evolutionary Trees. IEEE/ACM Trans. Comput. Biol. Bioinformatics. 2015;12:807–14. doi:10.1109/TCBB.2014.2366756.
214. Mattson DC, Yang J. The Child Therapy Tracking System (CTTS): A model for an expressive therapy electronic health record (EHR). ARTS IN PSYCHOTHERAPY. 2013;40:509–18. doi:10.1016/j.aip.2013.08.002.
215. Meamarzadeh H, Khayyambashi MR, Saraee MH. Extracting Temporal Rules from Medical Data. In: ; 2009. p. 327–331. doi:10.1109/ICCTD.2009.72.
216. Mechkour M, Mulhem P, Fourel F, Berrut EFC. PRIME-GC. A medical information retrieval prototype on the Web. In: ; 1997. p. 2–9. doi:10.1109/RIDE.1997.583681.
217. Mendonça EA, Johnson SB, Seol Y, Cimino JJ. Analyzing the Semantics of Patient Data to Rank Records of Literature Retrieval. In: Stroudsburg, PA, USA: Association for Computational Linguistics; 2002. p. 69–76. doi:10.3115/1118149.1118159.
218. Meystre SM, Lee S, Jung CY, Chevrier RD. Common data model for natural language processing based on two existing standard information models: CDA+GrAF. J Biomed Inform. 2012;45:703–10. doi:10.1016/j.jbi.2011.11.018.
219. Milicchio F, Tradigo G, Veltri P, Prosperi M. High-performance Data Structures for De Novo Assembly of Genomes: Cache Oblivious Generic Programming. In: New York, NY, USA: ACM; 2016. p. 657–662. doi:10.1145/2975167.2985691.
220. Mirshahi A, Kohnen T. Scientific evaluation and quality assurance in refractive surgical interventions. Evaluation of the Datagraph med computer program. Ophthalmologe. 2002;99:629–35.
221. Mirzaei M, Truswell AS, Arnett K, Page A, Taylor R, Leeder SR. Cerebrovascular disease in 48 countries: secular trends in mortality 1950-2005. J Neurol Neurosurg Psychiatry. 2012;83:138–45. doi:10.1136/jnnp-2011-300408.
222. Mondal S, Mukherjee N. Mobile-assisted remote healthcare delivery. In: ; 2016. p. 630–635. doi:10.1109/PDGC.2016.7913199.
223. Monsen KA, Banerjee A, Das P. Discovering client and intervention patterns in home visiting data. West J Nurs Res. 2010;32:1031–54. doi:10.1177/0193945910370970.
224. Morin P, Herrmann F, Ammann P, Uebelhart B, Rizzoli R. A rapid self-administered food frequency questionnaire for the evaluation of dietary protein intake. Clin Nutr. 2005;24:768–74. doi:10.1016/j.clnu.2005.03.002.
225. Moruzzi M. The new culture of dematerialized health. Recenti Prog Med. 2014;105:407–9. doi:10.1701/1680.18398.
226. Mossel E, Roch S, Steel M. Shrinkage Effect in Ancestral Maximum Likelihood. IEEE/ACM Trans. Comput. Biol. Bioinformatics. 2009;6:126–33. doi:10.1109/TCBB.2008.107.
227. Mozhayskiy V, Miller B, Ma K, Tagkopoulos I. A Scalable Multi-scale Framework for Parallel Simulation and Visualization of Microbial Evolution. In: New York, NY, USA: ACM; 2011. p. 7:1. doi:10.1145/2016741.2016749.

228. Muller R. The CliniCon framework for context representation in electronic patient records. Proc AMIA Annu Fall Symp. 1997:178–82.
229. Muller R. The CliniCon framework for context representation in electronic patient records. JOURNAL OF THE AMERICAN MEDICAL INFORMATICS ASSOCIATION. 1997:178–82.
230. Muller R, Sergl M, Nauerth U, Schoppe D, Pommerening K, Dittrich HM. THEMPO: a knowledge-based system for therapy planning in pediatric oncology. Comput Biol Med. 1997;27:177–200.
231. Muller R, Thews O, Rohrbach C, Sergl M, Pommerening K. A graph-grammar approach to represent causal, temporal and other contexts in an oncological patient record. Methods Inf Med. 1996;35:127–41.
232. Murphy CA, Carstens K, Villamayor P. Electronic growth charts: watching our patients grow. AMIA Annu Symp Proc. 2005:1058.
233. Mwaniki P, Ayieko P, Todd J, English M. Assessment of paediatric inpatient care during a multifaceted quality improvement intervention in Kenyan district hospitals--use of prospectively collected case record data. BMC Health Serv Res. 2014;14:312. doi:10.1186/1472-6963-14-312.
234. Myleus A, Stenlund H, Hernell O, Gothefors L, Hammarstrom M, Persson L, Ivarsson A. Early vaccinations are not risk factors for celiac disease. Pediatrics. 2012;130:e63-70. doi:10.1542/peds.2011-2806.
235. Nagar A, Al-Mubaid H, Bettayeb S. Computing Gene Functional Similarity Using Combined Graphs. In: New York, NY, USA: ACM; 2012. p. 1381–1386. doi:10.1145/2245276.2231995.
236. Newell SD, JR, Englert J, Box-Taylor A, Davis KM, Koch KE. Clinical efficiency tools improve stroke management in a rural southern health system. Stroke. 1998;29:1092–8.
237. Ng K, Ghoting A, Steinhubl SR, Stewart WF, Malin B, Sun J. PARAMO: a PARAllel predictive MOdeling platform for healthcare analytic research using electronic health records. J Biomed Inform. 2014;48:160–70. doi:10.1016/j.jbi.2013.12.012.
238. Ni J, Fei H, Fan W, Zhang X. Automated Medical Diagnosis by Ranking Clusters Across the Symptom-Disease Network. In: ; 2017. p. 1009–1014. doi:10.1109/ICDM.2017.130.
239. Nielsen F\. A Fielded Wiki for Personality Genetics. In: New York, NY, USA: ACM; 2010. p. 16:1. doi:10.1145/1832772.1832795.
240. Nikfarjam A, Emadzadeh E, Gonzalez G. Towards generating a patient's timeline: extracting temporal relationships from clinical notes. J Biomed Inform. 2013;46 Suppl:S40-7. doi:10.1016/j.jbi.2013.11.001.
241. Norén GN, Bate A, Hopstadius J, Star K, Edwards IR. Temporal Pattern Discovery for Trends and Transient Effects: Its Application to Patient Records. In: New York, NY, USA: ACM; 2008. p. 963–971. doi:10.1145/1401890.1402005.
242. Noren GN, Hopstadius J, Bate A, Star K, Edwards IR. Temporal pattern discovery in longitudinal electronic patient records. DATA MINING AND KNOWLEDGE DISCOVERY. 2010;20:361–87. doi:10.1007/s10618-009-0152-3.
243. Nori N, Kashima H, Yamashita K, Ikai H, Imanaka Y. Simultaneous Modeling of Multiple Diseases for Mortality Prediction in Acute Hospital Care. In: New York, NY, USA: ACM; 2015. p. 855–864. doi:10.1145/2783258.2783308.
244. Nose Y, Akazawa K, Watanabe Y, Yokota M, Okamura S, Maehara Y, Sugimachi K. Cancer registration using case history database in hospital information system. Jpn Hosp. 1988;7:21–8.
245. Ogushi Y, Haruki Y, Okada Y, Takahashi M, Shimizu M, Izumi Y, et al. Development and evaluation of regional health database systems. Stud Health Technol Inform. 1998;52 Pt 2:1297–300.
246. Okamoto K, Tanaka H, Takemura T, Kume N, Kuroda T, Yoshihara H. A hypothesis-generating support system using medical records for clinical knowledge acquisition. In: ; 2012. p. 1130–1133. doi:10.1109/SCIS-ISIS.2012.6505123.
247. Oladimeji EA, Chung L, Jung HT, Kim J. Managing Security and Privacy in Ubiquitous eHealth Information Interchange. In: New York, NY, USA: ACM; 2011. p. 26:1. doi:10.1145/1968613.1968645.
248. O'Neil M, Payne C, Read J. Read Codes Version 3: a user led terminology. Methods Inf Med. 1995;34:187–92.
249. Onimura N, Yamashita T, Nakayama N, Soejima H, Hirokawa S. Generation of Sentence Template Graph from SOAP Format Medical Documents. In: ; 2016. p. 159–162. doi:10.1109/CSCI.2016.0037.
250. Orban K, Edberg A, Erlandsson L. Using a time-geographical diary method in order to facilitate reflections on changes in patterns of daily occupations. Scand J Occup Ther. 2012;19:249–59. doi:10.3109/11038128.2011.620981.
251. Oshiro T, Oshiro H, Tanimizu M. Effective methods of organizing complex information in advanced cancer patients. Gan To Kagaku Ryoho. 2014;41 Suppl 1:26–9.
252. Osler M, Prescott E, Wium-Andersen IK, Ibfelt EH, Jorgensen MB, Andersen PK, et al. The Impact of Comorbid Depression on Educational Inequality in Survival after Acute Coronary Syndrome in a Cohort of 83 062 Patients and a Matched Reference Population. PLoS One. 2015;10:e0141598. doi:10.1371/journal.pone.0141598.
253. Osop H, Sahama T. Electronic health records: Improvement to healthcare decision-making. In: ; 2016. p. 1–6. doi:10.1109/HealthCom.2016.7749474.
254. O'Sullivan EM. International variation in the incidence of oral and pharyngeal cancer. Community Dent Health. 2008;25:148–53.
255. Pabón MC, Montoya GA, Millán M. Mediation and graph data models for medical data integration. In: ; 2013. p. 1–9. doi:10.1109/CLEI.2013.6670647.
256. Parkin DM, Ferlay J, Curado M, Bray F, Edwards B, Shin H, Forman D. Fifty years of cancer incidence: CI5 I-IX. Int J Cancer. 2010;127:2918–27. doi:10.1002/ijc.25517.

257. Pathak J, Kiefer RC, Chute CG. Mining drug-drug interaction patterns from linked data: A case study for Warfarin, Clopidogrel, and Simvastatin. In: ; 2013. p. 23–30. doi:10.1109/BIBM.2013.6732595.
258. Patil R, Karandikar RG. Digital signal preservation approaches of archived biomedical paper records #x2014; A review. In: ; 2016. p. 1–4. doi:10.1109/WECON.2016.7993456.
259. Paulson EH, Gerberich SG, Alexander BH, Ryan A, Renier CM, Zhang X, et al. Fall-related injuries among agricultural household members: Regional Rural Injury Study II (RRIS-II). J Occup Environ Med. 2006;48:959–68. doi:10.1097/01.jom.0000225110.89501.a3.
260. Pawloski P, Cusick D, Amborn L. Development of clinical pharmacy productivity metrics. AMERICAN JOURNAL OF HEALTH-SYSTEM PHARMACY. 2012;69:49–54. doi:10.2146/ajhp110126.
261. Payne TH, Andrews RD, Breeling J, Ben Davoren J, Smith RM, Volpp B. Graphing clinical events in the VA Computerized Patient Record System. JOURNAL OF THE AMERICAN MEDICAL INFORMATICS ASSOCIATION. 1999:1137.
262. Pedreschi D. Social Network Analytics, Data Science Ethics & Privacy-preserving Analytics. In: New York, NY, USA: ACM; 2017. doi:10.1145/3168836.3168841.
263. Penfield W. Physicians take to the field. IDRC Rep. 1992;20:28–30.
264. Pfeiffer KP. Actual state and perspectives of e-health in Austria and international--an overview. Wien Med Wochenschr. 2011;161:334–40. doi:10.1007/s10354-011-0008-5.
265. Pfundner A, Schnoeberg T, Horn J, Boyce RD, Samwald M. Utilizing the Wikidata System to Improve the Quality of Medical Content in Wikipedia in Diverse Languages: A Pilot Study. JOURNAL OF MEDICAL INTERNET RESEARCH 2015. doi:10.2196/jmir.4163.
266. Pietrzyk PM. Free text analysis. Int J Biomed Comput. 1995;39:139–44.
267. Poon-Chue A, Menendez L, Gerstner MM, Colletti P, Terk M. MRI evaluation of post-operative seromas in extremity soft tissue sarcomas. SKELETAL RADIOLOGY. 1999;28:279–82. doi:10.1007/s002560050516.
268. Poscia R, Ghio S, D'Alto M, Vitulo P, Mule M, Albera C, et al. 'Real-life' information on pulmonary arterial hypertension: the iPHnet Project. Curr Med Res Opin. 2014;30:2409–14. doi:10.1185/03007995.2014.960514.
269. Pourriat JL, Huet B, Gabry AL, Rolland C, Cupa M. Case summaries in real-time. Accomplishments with a microcomputer. Ann Fr Anesth Reanim. 1982;1:161–5.
270. Poursadegh M, Poursadegh F, Esmaeili M, Bakhshaee M. Epidemiological Survey of Sinonasal Malignancy in North-East Iran. Iran J Otorhinolaryngol. 2015;27:225–9.
271. Powsner SM, Tufte ER. Summarizing clinical psychiatric data. Psychiatr Serv. 1997;48:1458–61. doi:10.1176/ps.48.11.1458.
272. Preece MHW, Hill A, Horswill MS, Karamatic R, Watson MO. Designing observation charts to optimize the detection of patient deterioriation: reliance on the subjective preferences of healthcare professionals is not enough. Aust Crit Care. 2012;25:238–52. doi:10.1016/j.aucc.2012.01.003.
273. Pryor J. A snapshot of rehabilitation referrals in rural New South Wales. Aust Health Rev. 2010;34:204–9. doi:10.1071/AH08713.
274. Puentes J, Batrancourt B, Atif J, Angelini E, Lecornu L, Zemirline A, et al. Integrated multimedia electronic patient record and graph-based image information for cerebral tumors. Comput Biol Med. 2008;38:425–37. doi:10.1016/j.compbiomed.2008.01.009.
275. Rajagopalan MR, Vellaipandiyan S. Big data framework for national E-governance plan. In: ; 2013. p. 1–5. doi:10.1109/ICTKE.2013.6756283.
276. Rakic N. Acute sensorineural hearing loss at the Otorhinolaryngology Department of the General Hospital in Subotica 1991-1996. Med Pregl. 1999;52:44–52.
277. RAMAYYA GP. AXAUDIT - ANESTHETIC AUDIT SYSTEM. INTERNATIONAL JOURNAL OF CLINICAL MONITORING AND COMPUTING. 1992;9:149–58. doi:10.1007/BF01145167.
278. Rassinoux AM, Michel PA, Wagner J, Baud R. Current trends with natural language processing. Medinfo. 1995;8 Pt 2:1657.
279. Rathe JC, Elliott PF. A cancer registry for the community radiation center. Cancer. 1979;43:198–204.
280. Ratib O. From multimodality digital imaging to multimedia patient record. Comput Med Imaging Graph. 1994;18:59–65.
281. Reiz B, Csato L. Bayesian Network Classifier for Medical Data Analysis. INTERNATIONAL JOURNAL OF COMPUTERS COMMUNICATIONS & CONTROL. 2009;4:65–72. doi:10.15837/ijccc.2009.1.2414.
282. Rhea S, Weber DJ, Poole C, Cairns C. Risk factors for hospitalization after dog bite injury: a case-cohort study of emergency department visits. Acad Emerg Med. 2014;21:196–203. doi:10.1111/acem.12312.
283. Rizvi SZR, Fong PW. Interoperability of Relationship- and Role-Based Access Control. In: New York, NY, USA: ACM; 2016. p. 231–242. doi:10.1145/2857705.2857706.
284. Rizvi SZR, Fong PW, Crampton J, Sellwood J. Relationship-Based Access Control for an Open-Source Medical Records System. In: New York, NY, USA: ACM; 2015. p. 113–124. doi:10.1145/2752952.2752962.
285. Rocha J. Graph Comparison by Log-Odds Score Matrices with Application to Protein Topology Analysis. IEEE/ACM Trans. Comput. Biol. Bioinformatics. 2011;8:564–9. doi:10.1109/TCBB.2010.59.
286. Rodnick JE. The use of automated ambulatory medical records. J Fam Pract. 1977;5:253–64.
287. Rothwell DJ, Cote RA, Cordeau JP, Boisvert MA. Developing a standard data structure for medical language--the SNOMED proposal. Proc Annu Symp Comput Appl Med Care. 1993:695–9.

288. Rotmensch M, Halpern Y, Tlimat A, Horng S, Sontag D. Learning a Health Knowledge Graph from Electronic Medical Records. Sci Rep. 2017;7:5994. doi:10.1038/s41598-017-05778-z.
289. Ruiz JG, Andrade AD, Hogue C, Karanam C, Akkineni S, Cevallos D, et al. The Association of Graph Literacy With Use of and Skills Using an Online Personal Health Record in Outpatient Veterans. J Health Commun. 2016;21:83–90. doi:10.1080/10810730.2016.1193915.
290. Ryu I, Siio I. TongueDx: A Tongue Diagnosis for Health Care on Smartphones. In: New York, NY, USA: ACM; 2014. p. 25:1. doi:10.1145/2582051.2582076.
291. Săcărea C, Şotropa D, Troancă D. Symptoms investigation by means of formal concept analysis for enhancing medical diagnoses. In: ; 2017. p. 1–5. doi:10.23919/SOFTCOM.2017.8115588.
292. Salinas O, Cosio G de, Clavel-Arcas C, Montoya J, Serpas M, Moran de Garcia S, Concha-Eastman A. An information system for injuries from external causes (SILEX): a successful project in El Salvador. Rev Panam Salud Publica. 2008;24:390–9.
293. Sato D, Freitas A. Understanding quality in data from hospital diagnosis and procedures: A statistical characterization. In: ; 2010. p. 1–4.
294. Sauleau EA, Paumier J, Buemi A. Medical record linkage in health information systems by approximate string matching and clustering. BMC Med Inform Decis Mak. 2005;5:32. doi:10.1186/1472-6947-5-32.
295. Schenk RJ, JR, Schenk J. Integration of remote blood glucose meter upload technology into a clinical pharmacist medication therapy management service. J Diabetes Sci Technol. 2011;5:188–91. doi:10.1177/193229681100500126.
296. Schultz SE, Rothwell DM, Chen Z, Tu K. Identifying cases of congestive heart failure from administrative data: a validation study using primary care patient records. Chronic Dis Inj Can. 2013;33:160–6.
297. Schultz SE, Rothwell DM, Chen Z, Tu K. Identifying cases of congestive heart failure from administrative data: a validation study using primary care patient records. Chronic Dis Inj Can. 2013;33:160–6.
298. Sevick MA, Zickmund S, Korytkowski M, Piraino B, Sereika S, Mihalko S, et al. Design, feasibility, and acceptability of an intervention using personal digital assistant-based self-monitoring in managing type 2 diabetes. Contemp Clin Trials. 2008;29:396–409. doi:10.1016/j.cct.2007.09.004.
299. Shahn Z, Ryan P, Madigan D. Predicting Health Outcomes from High-Dimensional Longitudinal Health Histories Using Relational Random Forests. STATISTICAL ANALYSIS AND DATA MINING. 2015;8:128–36. doi:10.1002/sam.11268.
300. Sharit J, Lisigurski M, Andrade AD, Karanam C, Nazi KM, Lewis JR, Ruiz JG. The Roles of Health Literacy, Numeracy, and Graph Literacy on the Usability of the VA's Personal Health Record by Veterans. J. Usability Studies. 2014;9:173–93.
301. Shaverdian AA, Zhou H, Michailidis G, Jagadish HV. Algebraic Visual Analysis: The Catalano Phone Call Data Set Case Study. In: New York, NY, USA: ACM; 2009. p. 74–82. doi:10.1145/1562849.1562858.
302. Shaw JLV, Cohen A, Konforte D, Binesh-Marvasti T, Colantonio DA, Adeli K. Validity of establishing pediatric reference intervals based on hospital patient data: a comparison of the modified Hoffmann approach to CALIPER reference intervals obtained in healthy children. Clin Biochem. 2014;47:166–72. doi:10.1016/j.clinbiochem.2013.11.008.
303. Sheridan J, Chamberlain K, Dupuis A. Timelining: visualizing experience. QUALITATIVE RESEARCH. 2011;11:552–69. doi:10.1177/1468794111413235.
304. Shibuya T. Geometric Suffix Tree: Indexing Protein 3-D Structures. J. ACM. 2010;57:15:1. doi:10.1145/1706591.1706595.
305. Shigli H, Tejas MH, Narayan L, Desai SD. User Intervention Based Segmentation of Myocardium In Cardiac Cine MRI Images. In: New York, NY, USA: ACM; 2015. p. 684–689. doi:10.1145/2791405.2791545.
306. Shim S, Kim D, Lee D, Lee S, Park J, Lee JJ, et al. Metabolic tumour volume and total lesion glycolysis, measured using preoperative 18F-FDG PET/CT, predict the recurrence of endometrial cancer. BJOG. 2014;121:1097-106; discussion 1106. doi:10.1111/1471-0528.12543.
307. Sills MR, Kwan BM, Yawn BP, Sauer BC, Fairclough DL, Federico MJ, et al. Medical home characteristics and asthma control: a prospective, observational cohort study protocol. EGEMS (Wash DC). 2013;1:1032. doi:10.13063/2327-9214.1032.
308. Sim LLW, Ban KHK, Tan TW, Sethi SK, Loh TP. Development of a clinical decision support system for diabetes care: A pilot study. PLoS One. 2017;12:e0173021. doi:10.1371/journal.pone.0173021.
309. Sittig DF, Murphy DR, Smith MW, Russo E, Wright A, Singh H. Graphical display of diagnostic test results in electronic health records: a comparison of 8 systems. J Am Med Inform Assoc. 2015;22:900–4. doi:10.1093/jamia/ocv013.
310. SMART JF, ROUX M. A MODEL FOR MEDICAL KNOWLEDGE REPRESENTATION APPLICATION TO THE ANALYSIS OF DESCRIPTIVE PATHOLOGY REPORTS. Methods Inf Med. 1995;34:352–60.
311. Sobo EJ, Andriese S, Stroup C, Morgan D, Kurtin P. Developing indicators for emergency medical services (EMS) system evaluation and quality improvement: a statewide demonstration and planning project. Jt Comm J Qual Improv. 2001;27:138–54.
312. Soldes OS, Younger JG, Hirschl RB. Predictors of malignancy in childhood peripheral lymphadenopathy. J Pediatr Surg. 1999;34:1447–52.

313. Sondhi P, Sun J, Tong H, Zhai C. SympGraph: A Framework for Mining Clinical Notes Through Symptom Relation Graphs. In: New York, NY, USA: ACM; 2012. p. 1167–1175. doi:10.1145/2339530.2339712.
314. Soulakis ND, Carson MB, Lee YJ, Schneider DH, Skeehan CT, Scholtens DM. Visualizing collaborative electronic health record usage for hospitalized patients with heart failure. J Am Med Inform Assoc. 2015;22:299–311. doi:10.1093/jamia/ocu017.
315. Sovik S, Skaga NO, Hanoa R, Eken T. Sudden survival improvement in critical neurotrauma: An exploratory analysis using a stratified statistical process control technique. Injury. 2014;45:1722–30. doi:10.1016/j.injury.2014.05.038.
316. Spidlen J, Hanzlicek P, Riha A, Zvarova J. Flexible information storage in MUDR(II) EHR. Int J Med Inform. 2006;75:201–8. doi:10.1016/j.ijmedinf.2005.07.044.
317. Sridhar S, Dhamdhere K, Blelloch G, Halperin E, Ravi R, Schwartz R. Algorithms for Efficient Near-Perfect Phylogenetic Tree Reconstruction in Theory and Practice. IEEE/ACM Trans. Comput. Biol. Bioinformatics. 2007;4:561–71. doi:10.1109/TCBB.2007.1070.
318. Staubert S, Schaaf M, Jahn F, Brandner R, Winter A. Modeling Interoperable Information Systems with 3LGM(2) and IHE. Methods Inf Med. 2015;54:398–405. doi:10.3414/ME14-02-0027.
319. Stefansson CG. Map analyses of psychiatric services. The application of a computerized psychiatric case register to geographical analysis. Acta Psychiatr Scand. 1984;70:515–22.
320. Stevens VJ, Rossner J, Greenlick M, Stevens N, Frankel HM, Craddick S. Freedom from fat: a contemporary multi-component weight loss program for the general population of obese adults. J Am Diet Assoc. 1989;89:1254–8.
321. Sumner W2, Truszczynski M, Marek VW. A formal model of family medicine. J Am Board Fam Pract. 1996;9:41–52.
322. Sun K, Goncalves JP, Larminie C, Przulj N. Predicting disease associations via biological network analysis. BMC Bioinformatics 2014. doi:10.1186/1471-2105-15-304.
323. Swartz SH, Cowan TM, DePue J, Goldstein MG. Academic profiling of tobacco-related performance measures in primary care. Nicotine Tob Res. 2002;4 Suppl 1:S38-44. doi:10.1080/14622200210128018.
324. Syalim A, Nishide T, Sakurai K. Securing Provenance of Distributed Processes in an Untrusted Environment. IEICE TRANSACTIONS ON INFORMATION AND SYSTEMS. 2012;E95D:1894–907. doi:10.1587/transinf.E95.D.1894.
325. Szeto LK, Liew AW, Yan H, Tang S. Gene Expression Data Clustering and Visualization Based on a Binary Hierarchical Clustering Framework. In: Darlinghurst, Australia, Australia: Australian Computer Society, Inc; 2003. p. 145–152.
326. Taffe J, Dennerstein L. Menstrual diary data and menopausal transition: methodologic issues. Acta Obstet Gynecol Scand. 2002;81:588–94.
327. Techentin R, Foti D, Li P, Daniel E, Gilbert B, Holmes D, Al-Saffar S. Development of a Semi-synthetic Dataset as a Testbed for Big-Data Semantic Analytics. In: ; 2014. p. 252–253. doi:10.1109/ICSC.2014.45.
328. Techentin R, Sauver JS, Huddleston J, Gilbert B, Holmes D. Lessons learned from the semantic translation of healthcare data. In: ; 2014. p. 513–518. doi:10.1109/HealthCom.2014.7001895.
329. Tezcan B, Khazaezadeh N, Ash A, Oteng-Ntim E. Social disparity and intrauterine death: from politics to policies. J Obstet Gynaecol. 2011;31:507–8. doi:10.3109/01443615.2011.587910.
330. Tiikkaja S, Sandin S, Hultman CM, Modin B, Malki N, Sparen P. Psychiatric disorder and work life: A longitudinal study of intra-generational social mobility. Int J Soc Psychiatry. 2016;62:156–66. doi:10.1177/0020764015614594.
331. Toner JP, Coddington CC, Doody K, van Voorhis B, Seifer DB, Ball GD, et al. Society for Assisted Reproductive Technology and assisted reproductive technology in the United States: a 2016 update. Fertil Steril. 2016;106:541–6. doi:10.1016/j.fertnstert.2016.05.026.
332. Torkar S, Benedik P, Rajkovic U, Sustersic O, Rajkovic V. Design of a Recommendation System for Adding Support in the Treatment of Chronic Patients. Stud Health Technol Inform. 2016;225:879–80.
333. Troszynski M, Niemiec T, Wilczynska A. Assessment of three-level selective perinatal care based on the analysis of early perinatal death rates and cesarean sections in Poland in 2008. Ginekol Pol. 2009;80:670–7.
334. Truyen Tran, Dinh Phung, Luo W, Venkatesh S. Stabilized sparse ordinal regression for medical risk stratification. KNOWLEDGE AND INFORMATION SYSTEMS. 2015;43:555–82. doi:10.1007/s10115-014-0740-4.
335. Tukiendorf A. Cervix uteri cancer incidence in relation to ethnic situation in Opole province, Poland. Cent Eur J Public Health. 2002;10:88–92.
336. Ugon A, Philippe C, Pietrasz S, Ganascia J, Levy PP. OPTISAS a new method to analyse patients with Sleep Apnea Syndrome. Stud Health Technol Inform. 2008;136:547–52.
337. Valko M, Kveton B, Valizadegan H, Cooper GF, Hauskrecht M. Conditional Anomaly Detection with Soft Harmonic Functions. Proc IEEE Int Conf Data Min. 2011;2011:735–43. doi:10.1109/ICDM.2011.40.
338. Vallejo IL, Herrero HH, Sanz JJ, Martin JJ, Azarola EN. Diagnostic variability in a cohort of patients with multiple admissions in the last two decades. ACTAS ESPANOLAS DE PSIQUIATRIA. 2003;31:18–23.
339. Vani SN. Appropriate technologies for mother and child health care in developing countries. Indian Pediatr. 1989;26:1124–30.

340. Vilares M, Ribadas FJ, Graña J. Approximately Common Patterns in Shared-forests. In: New York, NY, USA: ACM; 2001. p. 73–80. doi:10.1145/502585.502599.
341. Voskoboinik A, Gutman MJ, Croagh D, Bell R, Saunder A, Gribbin J, Kanellis J. Implementation and learning of laproscopic donor nephrectomy by a non-transplant general surgeon with advanced laparoscopic skills. Asian J Endosc Surg. 2011;4:127–32. doi:10.1111/j.1758-5910.2011.00092.x.
342. Vossler DG, Am Haltiner, Schepp SK, Friel PA, Caylor LM, Morgan JD, Doherty MJ. Ictal stuttering - A sign suggestive of psychogenic nonepileptic seizures. Neurology. 2004;63:516–9. doi:10.1212/01.WNL.0000133208.57562.CB.
343. Vossler DG, Haltiner AM, Schepp SK, Friel PA, Caylor LM, Morgan JD, Doherty MJ. Ictal stuttering: a sign suggestive of psychogenic nonepileptic seizures. Neurology. 2004;63:516–9.
344. Vranken R, Coulombier D, Kenyon T, Koosimile B, Mavunga T, Coggin W, Binkin N. Use of a computerized tuberculosis register for automated generation of case finding, sputum conversion, and treatment outcome reports. Int J Tuberc Lung Dis. 2002;6:111–20.
345. Walley JD, McDonald M. Integration of mother and child health services in Ethiopia. Trop Doct. 1991;21:32–5. doi:10.1177/004947559102100113.
346. Wang Z, Chakraborty P, Mekaru SR, Brownstein JS, Ye J, Ramakrishnan N. Dynamic Poisson Autoregression for Influenza-Like-Illness Case Count Prediction. In: New York, NY, USA: ACM; 2015. p. 1285–1294. doi:10.1145/2783258.2783291.
347. Wang S, Chang X, Li X, Long G, Yao L, Sheng QZ. Diagnosis Code Assignment Using Sparsity-Based Disease Correlation Embedding. IEEE TRANSACTIONS ON KNOWLEDGE AND DATA ENGINEERING. 2016;28:3191–202. doi:10.1109/TKDE.2016.2605687.
348. Wang JT, Chirn G, Marr TG, Shapiro B, Shasha D, Zhang K. Combinatorial Pattern Discovery for Scientific Data: Some Preliminary Results. In: New York, NY, USA: ACM; 1994. p. 115–125. doi:10.1145/191839.191863.
349. Wang JT, Chirn G, Marr TG, Shapiro B, Shasha D, Zhang K. Combinatorial Pattern Discovery for Scientific Data: Some Preliminary Results. SIGMOD Rec. 1994;23:115–25. doi:10.1145/191843.191863.
350. Wang B, Lin C, Yang I. Constructing a Gene Team Tree in Almost O (N Lg N) Time. IEEE/ACM Trans. Comput. Biol. Bioinformatics. 2014;11:142–53. doi:10.1109/TCBB.2013.150.
351. Wang F, Liu C, Wang Y, Hu J, Yu G. A Graph Based Methodology for Temporal Signature Identification from HER: RETRACTED ARTICLE - NICHT BENUTZEN, ABGELEHNTER ARTIKEL. AMIA Annu Symp Proc. 2015;2015:1269–78.
352. Wang X, Wang F, Wang J, Qian B, Hu J. Exploring Patient Risk Groups with Incomplete Knowledge. In: ; 2013. p. 1223–1228. doi:10.1109/ICDM.2013.129.
353. Welch JL, Siek KA, Connelly KH, Astroth KS, McManus MS, Scott L, et al. Merging health literacy with computer technology: self-managing diet and fluid intake among adult hemodialysis patients. Patient Educ Couns. 2010;79:192–8. doi:10.1016/j.pec.2009.08.016.
354. White D, Choi H, Peloquin C, Zhu Y, Zhang Y. Secular trend of adhesive capsulitis. Arthritis Care Res (Hoboken). 2011;63:1571–5. doi:10.1002/acr.20590.
355. Whyatt CP, Torres EB. The Social-dance: Decomposing Naturalistic Dyadic Interaction Dynamics to the 'Micro-level'. In: New York, NY, USA: ACM; 2017. p. 24:1. doi:10.1145/3077981.3078055.
356. Wiesner M, Pfeifer D. Adapting Recommender Systems to the Requirements of Personal Health Record Systems. In: New York, NY, USA: ACM; 2010. p. 410–414. doi:10.1145/1882992.1883053.
357. Wilhelms J, van Gelder A. Octrees for Faster Isosurface Generation. ACM Trans. Graph. 1992;11:201–27. doi:10.1145/130881.130882.
358. Winter A, Brigl B, Funkat G, Haeber A, Heller O, Wendt T. 3LGM(2)-Modeling to support management of health information systems. Int J Med Inform. 2007;76:145–50. doi:10.1016/j.ijmedinf.2006.07.007.
359. Wongsuphasawat K, Gotz D. Exploring Flow, Factors, and Outcomes of Temporal Event Sequences with the Outflow Visualization. IEEE Trans Vis Comput Graph. 2012;18:2659–68. doi:10.1109/TVCG.2012.225.
360. Wu T, Moulton V, Steel M. Refining Phylogenetic Trees Given Additional Data: An Algorithm Based on Parsimony. IEEE/ACM Trans. Comput. Biol. Bioinformatics. 2009;6:118–25. doi:10.1109/TCBB.2008.100.
361. WU Y, Zhu X, Li L, Fan W, Jin R, Zhang X. Mining Dual Networks: Models, Algorithms, and Applications. ACM Trans. Knowl. Discov. Data. 2016;10:40:1. doi:10.1145/2785970.
362. Xie W, Wu J. Mining positive and negative weighted association rules in medical records without user-specified weights based on HITS model. In: ; 2010. p. 2325–2329. doi:10.1109/BMEI.2010.5639578.
363. Xie C, Yang P, Yang Y. Open Knowledge Accessing Method in IoT-based Hospital Information System for Medical Record Enrichment. IEEE Access. 2018;PP:1. doi:10.1109/ACCESS.2018.2810837.
364. Yamashita T, Onimura N, Soejima H, Nakashima N, Hirokawa S. Graph Clustering System for Text-Based Records in a Clinical Pathway. Stud Health Technol Inform. 2017;245:649–52.
365. Yamin A, Khan SA, Yasin UU. Automated system of hess screen for diagnosis of paralytic strabismus using computer aided diagnosis. In: ; 2013. p. 300–305. doi:10.1109/IST.2013.6729710.
366. Yesha R, Gangopadhyay A, Siegel E. A graph-based method for analyzing electronic medical records. In: ; 2015. p. 1036–1041. doi:10.1145/2808797.2808806.
367. Yin S, Chen D, Le J. Deep Neural Network Based on Translation Model for Diabetes Knowledge Graph. In: ; 2017. p. 318–323. doi:10.1109/CBD.2017.62.

368. Yoon S, Cohen B, Cato KD, Liu J, Larson EL. Visualization of Data Regarding Infections Using Eye Tracking Techniques. J Nurs Scholarsh. 2016;48:244–53. doi:10.1111/jnu.12204.
369. Yoon D, Park I, Schuemie MJ, Park MY, Kim JH, Park RW. A quantitative method for assessment of prescribing patterns using electronic health records. PLoS One. 2013;8:e75214. doi:10.1371/journal.pone.0075214.
370. Yousefi A, Mastouri N, Sartipi K. Scenario-oriented information extraction from electronic health records. In: ; 2009. p. 1–5. doi:10.1109/CBMS.2009.5255451.
371. Zamora M, Baradad M, Amado E, Cordomí S, Limón E, Ribera J, et al. Characterizing chronic disease and polymedication prescription patterns from electronic health records. In: ; 2015. p. 1–9. doi:10.1109/DSAA.2015.7344870.
372. Zenios S. Information Technology in Health Care Systems: Barriers to Adoption. In: ; 2006. p. 52. doi:10.1109/ICCGI.2006.35.
373. Zhang J, Gong J, Barnes L. HCNN: Heterogeneous Convolutional Neural Networks for Comorbid Risk Prediction with Electronic Health Records. In: ; 2017. p. 214–221. doi:10.1109/CHASE.2017.80.
374. Zhang S, Liu L, Li H, Xiao Z, Cui L. MTPGraph: A Data-Driven Approach to Predict Medical Risk Based on Temporal Profile Graph. In: ; 2016. p. 1174–1181. doi:10.1109/TrustCom.2016.0191.
375. Zhang H, Mehotra S, Liebovitz D, Gunter CA, Malin B. Mining Deviations from Patient Care Pathways via Electronic Medical Record System Audits. ACM Trans. Manage. Inf. Syst. 2013;4:17:1. doi:10.1145/2544102.
376. Zhang P, Wang F, Hu J, Sorrentino R. Towards personalized medicine: leveraging patient similarity and drug similarity analytics. AMIA Jt Summits Transl Sci Proc. 2014;2014:132–6.
377. Zhang Z, Wang H, Wang C, Fang H. Cluster-based Epidemic Control Through Smartphone-based Body Area Networks. IEEE Trans Parallel Distrib Syst. 2015;26:681–90. doi:10.1109/TPDS.2014.2313331.
378. Zhang M, Zhang H, Tjandra D, Wong STC. DBMap: a space-conscious data visualization and knowledge discovery framework for biomedical data warehouse. IEEE Transactions on Information Technology in Biomedicine. 2004;8:343–53. doi:10.1109/TITB.2004.832550.
379. Zhao S. Mining Medical Causality for Diagnosis Assistance. In: New York, NY, USA: ACM; 2017. p. 841. doi:10.1145/3018661.3022752.
380. Zheng Z, Wan X. Graph-Based Multi-Modality Learning for Clinical Decision Support. In: New York, NY, USA: ACM; 2016. p. 1945–1948. doi:10.1145/2983323.2983880.
381. Zhou X, Han H, Chankai I, Prestrud A, Brooks A. Approaches to Text Mining for Clinical Medical Records. In: New York, NY, USA: ACM; 2006. p. 235–239. doi:10.1145/1141277.1141330.
382. Zirkind G. Genetic Database Optimization: How Data Inspection and Consideration, Provides for Index Compression and Record Access Optimization of Genetic Databases. In: New York, NY, USA: ACM; 2006. p. 68–76. doi:10.1145/1125170.1125194.
383. ZWEIGENBAUM P. MENELAS - AN ACCESS SYSTEM FOR MEDICAL RECORDS USING NATURAL-LANGUAGE. Comput Methods Programs Biomed. 1994;45:117–20. doi:10.1016/0169-2607(94)90029-9.
